# A metabolic profile of xenon and metabolite associations with 6-month mortality after out-of-hospital cardiac arrest: A post-hoc study of the randomised Xe-Hypotheca trial

**DOI:** 10.1371/journal.pone.0304966

**Published:** 2024-06-04

**Authors:** Aleksi J. Nummela, Harry Scheinin, Markus Perola, Anni Joensuu, Ruut Laitio, Olli Arola, Juha Grönlund, Risto O. Roine, Minna Bäcklund, Tero J. Vahlberg, Timo Laitio, for the Xe-Hypotheca Collaboration Group

**Affiliations:** 1 Department of Internal Medicine, Turku University Hospital, University of Turku, Turku, Finland; 2 Department of Perioperative Services, Intensive Care and Pain Management, Turku University Hospital, University of Turku, Turku, Finland; 3 Integrative Physiology and Pharmacology, Institute of Biomedicine, University of Turku, Turku, Finland; 4 Finnish Institute for Health and Welfare, Helsinki, Finland; 5 Faculty of Medicine, Research Program for Clinical and Molecular Metabolism, University of Helsinki, Helsinki, Finland; 6 Division of Clinical Neurosciences, University of Turku, Turku University Hospital, Turku, Finland; 7 Department of Anesthesiology, Intensive Care and Pain Medicine, Division of Intensive Care Medicine, University of Helsinki and HUS Helsinki University Hospital, Helsinki, Finland; 8 Department of Biostatistics, University of Turku and Turku University Hospital, Turku, Finland; Brigham and Women’s Hospital, UNITED STATES

## Abstract

**Purpose:**

Out-of-hospital cardiac arrest (OHCA) carries a relatively poor prognosis and requires multimodal prognostication to guide clinical decisions. Identification of previously unrecognized metabolic routes associated with patient outcome may contribute to future biomarker discovery. In OHCA, inhaled xenon elicits neuro- and cardioprotection. However, the metabolic effects remain unknown.

**Materials and methods:**

In this post-hoc study of the randomised, 2-group, single-blind, phase 2 Xe-Hypotheca trial, 110 OHCA survivors were randomised 1:1 to receive targeted temperature management (TTM) at 33°C with or without inhaled xenon during 24 h. Blood samples for nuclear magnetic resonance spectroscopy metabolic profiling were drawn upon admission, at 24 and 72 h.

**Results:**

At 24 h, increased lactate, adjusted hazard-ratio 2.25, 95% CI [1.53; 3.30], p<0.001, and decreased branched-chain amino acids (BCAA) leucine 0.64 [0.5; 0.82], p = 0.007, and valine 0.37 [0.22; 0.63], p = 0.003, associated with 6-month mortality. At 72 h, increased lactate 2.77 [1.76; 4.36], p<0.001, and alanine 2.43 [1.56; 3.78], p = 0.001, and decreased small HDL cholesterol ester content (S-HDL-CE) 0.36 [0.19; 0.68], p = 0.021, associated with mortality. No difference was observed between xenon and control groups.

**Conclusions:**

In OHCA patients receiving TTM with or without xenon, high lactate and alanine and decreased BCAAs and S-HDL-CE associated with increased mortality. It remains to be established whether current observations on BCAAs, and possibly alanine and lactate, could reflect neural damage via their roles in the metabolism of the neurotransmitter glutamate. Xenon did not significantly alter the measured metabolic profile, a potentially beneficial attribute in the context of compromised ICU patients.

**Trial registration:**

**Trial Registry number:** ClinicalTrials.gov Identifier: NCT00879892.

## Introduction

Out-of-hospital cardiac arrest (OHCA) carries a relatively poor overall prognosis, regardless of the advances in short term survival from the time of OHCA to hospital admission and improving trends in outcome after admission into the intensive care unit (ICU) [1,2]. Targeted temperature management (TTM) has become the standard-of-care following successful resuscitation after OHCA with the benefits on survival and functional neurological outcome having been demonstrated in previous landmark trials [3,4]. The current post-resuscitation care recommendations reflect the recent data demonstrating no clinical advantage of using the earlier mild hypothermic target of 33°C instead of TTM with a normothermic target of 36–37.5°C [5,6].

The noble gas xenon is a near-perfect anaesthetic as it is not metabolised, it is remarkably safe and efficient, has a minimal effect on hemodynamics and possess very rapid induction and recovery characteristics due to its extremely low solubility [7–9]. In the context of OHCA and TTM, inhaled xenon has been shown to attenuate myocardial damage and reduce cerebral white matter damage in comatose OHCA survivors treated with TTM at 33°C [10,11]. Concerning the possible future utilisation of xenon in compromised ICU patients, the metabolic effects of the anaesthetic should be investigated.

Since the prediction of outcome in post-resuscitation OHCA is a challenge, it is recommended that a reliable prognostic estimation can best be achieved by adopting multimodal approaches combining neurological examination, electroencephalography, MRI imaging and circulating biomarkers of hypoxic-ischaemic cerebral damage. Nonetheless, the optimal multimodal model remains to be established [12] and the search for viable biomarkers is ongoing [13–15].

In metabolomics, large-scale data capturing methods drive biomarker discovery with the goal of providing insights in early diagnostics and eventually enhancing early prediction of treatment outcomes [16–18]. The identification of previously unrecognised metabolic routes associated with patient outcome might make a valuable contribution to future biomarker discovery and, ultimately to clinical decision-making.

In this explorative post-hoc study of the Xe-Hypotheca trial, by conducting nuclear magnetic resonance spectroscopy (NMR) metabolic profiling from repeated blood samples, we aimed to discover previously unrecognised associations between the metabolic profile in the systemic circulation and 6-month mortality after OHCA, in patients undergoing TTM at a target temperature of 33°C with or without inhaled xenon. Furthermore, to our knowledge, this is the first study assessing the metabolic profile of inhaled xenon in the context of OHCA or in any other patient group.

## Material and methods

### Ethics

Ethical approval for this study (Xe-Hypotheca, EUDRA CT: 2009-009505-25) was provided by the Ethics Committee of Hospital District of Southwest Finland, Turku, Finland (Chairperson Janne Aso) on 17.03.2009 and the institutional review boards of the Helsinki University Hospital and the Finnish Medicines Agency. Furthermore, the data was reviewed after enrollment of every 4 patients and after each 6-month interval by an independent data and safety monitoring committee.

Written informed assent was obtained within 4 h after hospital arrival from the next of kin or from a legal representative of the patient in accordance with the Declaration of Helsinki. The right to withdraw and the use of data collected prior to possible withdrawal, as predefined in the trial protocol, was informed to each patient’s family. If patients regained consciousness, they were informed accordingly.

### Trial design and participants

Xe-Hypotheca trial (ClinicalTrials.gov NCT00879892) was a randomised, 2-group, single-blind, phase 2 clinical drug trial conducted at 2 multipurpose ICUs in Finland. The complete trial protocol has been published earlier in an article assessing cerebral white matter damage in comatose survivors of OHCA [10].

Comatose survivors of OHCA consecutively admitted to Turku and Helsinki University hospitals were assessed for eligibility (n = 224). The main inclusion criteria included witnessed cardiac arrest from a shockable initial rhythm (ventricular fibrillation or non-perfusing ventricular tachycardia) and a return of spontaneous circulation within 45 minutes, as reported previously [10]. A total of 110 patients were recruited between 5^th^ of August 2009 and 9^th^ of September 2014.

Patients were randomised 1:1 to receive TTM at 33°C alone or in combination with inhaled xenon (LENOXe, Air Liquide Medical GmbH) during the first 24 h after hospital admission. The groups are subsequently defined as the control and xenon group. Randomisation was done using permuted blocks with block sizes of 4, 6 and 8.

### Treatment protocol

The cooling of patients and administration of xenon treatment followed a detailed treatment protocol. Other procedures (e.g. coronary angiographic interventions) were conducted when clinically indicated and integrated care after cardiac arrest and patient monitoring adhered to recommendations [10].

After randomisation, inhaled xenon treatment was immediately initiated with an end-tidal xenon concentration of at least 40% delivered by a closed-system ventilator (PhysioFlex, Dräger) with the end-tidal concentration being continuously monitored [10,11].

### Blood sampling and metabolomic analysis

A post-hoc explorative metabolomic analysis was conducted upon admission, at 24 hours and at 72 hours post-cardiac arrest. A 10 ml blood sample was drawn and stored at -70 C. Sampling, storage and transfer of the metabolomic samples were carried out as specified by the company responsible for metabolite quantification. A targeted high-throughput nuclear magnetic resonance spectroscopy (NMR) platform was used for biomarker quantification (Nightingale Health Ltd, Helsinki, Finland). A detailed description of the current NMR methodology has been reported previously [19,20].

In all, 146 metabolites and 9 calculated ratios were analysed: 101 lipoprotein and 37 lipid-related (including 16 fatty acid, 9 cholesterol, 9 glycerides and phospholipids and 3 apolipoprotein) metabolite markers, concentrations of 8 amino-acids, 3 glycolysis related metabolites, 3 ketone bodies, creatinine, albumin as well as an inflammatory marker, glycoprotein acetylation.

### Statistical analysis

Two-sample t-test and Mann-Whitney U-test were used to compare continuous characteristic variables between xenon and control groups. Categorical characteristic variables were compared with chi-square test or Fisher exact test. Logarithmic transformation was performed for metabolites due to their skewness >1 (46.5% of all metabolites in the 6-month mortality analysis), S1 Table. Metabolites were scaled to baseline standard deviation (SD) to enable comparisons across metabolites with different units and comparison of metabolite changes between timepoints.

Associations of metabolites with 6-month mortality were analyzed with Cox regression after adjusting for age, sex, center and group. The age, sex and center adjusted mean differences in metabolites between the xenon and control groups were analyzed using linear mixed model with random intercept for patient. Model included the main effects for group and time and group × time interaction effect. From this model, the group difference of metabolite change from baseline to 24 hours and from baseline to 72 hours was estimated using contrasts.

There was a high correlation between several of the measured metabolite values. Thus, principal component analysis was carried out for each time point, and over 95% of variation in metabolomic data was explained by 14 components in all three time points. Thus, all reported p-values have been adjusted by a factor of 14 to account for multiple testing with an alpha threshold of 0.05 being used [21]. The results are reported as adjusted hazard ratios [95% confidence interval] and adjusted p-values, if not otherwise stated. Statistical analyses were carried out with SAS software (version 9.4; SAS Institute Inc., Cary, NC).

Data visualisation was created using R (Version 1.1.383, https://www.R-project.org/) gglpot2 function (Version 3.2.1, https://ggplot2.tidyverse.org).

## Results

A total of 224 patients were screened for eligibility, 110 patients were randomised 1:1 to control and xenon groups. Of these, one patient in the xenon group was withdrawn from the study by the next of kin; as predefined in the study protocol, the data obtained prior to withdrawal was included in the current analysis. Patient flow from enrolment to analysis is shown in supporting information S1 Fig. Patient demographics are shown in Table 1. The metabolic profiles of 105 patients were available for analysis; within this group, 6-month mortality was 30.5% (32/105). The mean ± SD age of survivors and non-survivors was 57.8 ± 11.9 and 64.6 ± 7.8 years, 67.1% of survivors were males as were 84.4% of non-survivors. As we have previously reported, the Kaplan-Meier survival estimate of the intention to treat population of 110 patients after 6-months was 27.3% and 34.5% in xenon *vs*. control groups, respectively, adjusted hazard ratio [95% CI], 0.49 [0.23, 1.01], p = 0.053 [10].

**Table 1:** Demographics.

	Control (n = 53)	Xenon (n = 52)	p-value
**Age, median (IQR), y**	60 (55–67)	63 (56–69)	0.52^b^
**Male sex**	38 (71.7)	38 (73.1)	0.87^c^
**Coronary artery disease**	39(73.6)	36 (69.2)	0.62^c^
**Hypertension**	25 (47.2)	20 (38.5)	0.37^c^
**Congestive heart failure**	3 (5.7)	6 (11.5)	0.32^d^
**Diabetes**	7 (13.2)	8(15.4)	0.75^c^
**Asthma or chronic obstructive pulmonary disease**	8 (15.1)	6(11.5)	0.59^c^
**Dyslipidemia**	21 (39.6)	15(28.9)	0.24^c^
**Smoker** *	21 (40.4)	15(30)	0.27^c^
**ST elevation myocardial infarction**	19 (35.9)	16 (30.8)	0.58^c^
**Previous stroke^**	13 (26.0)	11 (22.9)	0.72^c^
**Resuscitation details**			
**Bystander resuscitation**	39 (73.6)	36 (69.2)	0.62^c^
**EMS delay, mean (SD), min**	8.8 (3.4)	8.3 (3.4)	0.58^a^
**ROSC, mean (SD), min**	22.0 (7.0)	22.0 (7.6)	0.99^a^
**No flow, median (IQR), min**	0 (0–0)	0 (0–6)	0.47^b^
**Treatment and sampling**			
**Core temperature before cooling, median (IQR), °C**	35.4 (34.0–36.3)	34.9 (34.3–35.8)	0.22^b^
**Time from OHCA to target temperature, median (IQR), h**	5.6 (4.35–6.58)	4.9 (4.24–5.67)	0.09^b^
**Cooling rate, median (IQR), °C/h**	0.43 (0.22–0.50)	0.42 (0.28–0.50)	0.79^b^
**Time from OHCA to initiation of xenon, median (IQR), min**		251 (210–282)	
**Time from OHCA to baseline metabolic sampling, median (IQR), h**	3.0 (1.8–4.3)	3.3 (1.8–4.3)	0.84^b^

Data are expressed as No. (%) unless otherwise stated. Abbreviations: IQR, interquartile range; SD, standard deviation; EMS, emergency medical services; ROSC, return of spontaneous circulation; OHCA, out-of-hospital cardiac arrest.

^a^Two sample t-test.

^b^Mann-Whitney U-test.

^c^Chi-square.

^d^Fisher’s exact test.

*data missing: 1 patient in control, 2 in xenon.

^data missing: 3 patients in control, 5 in xenon.

**Fig 1:**
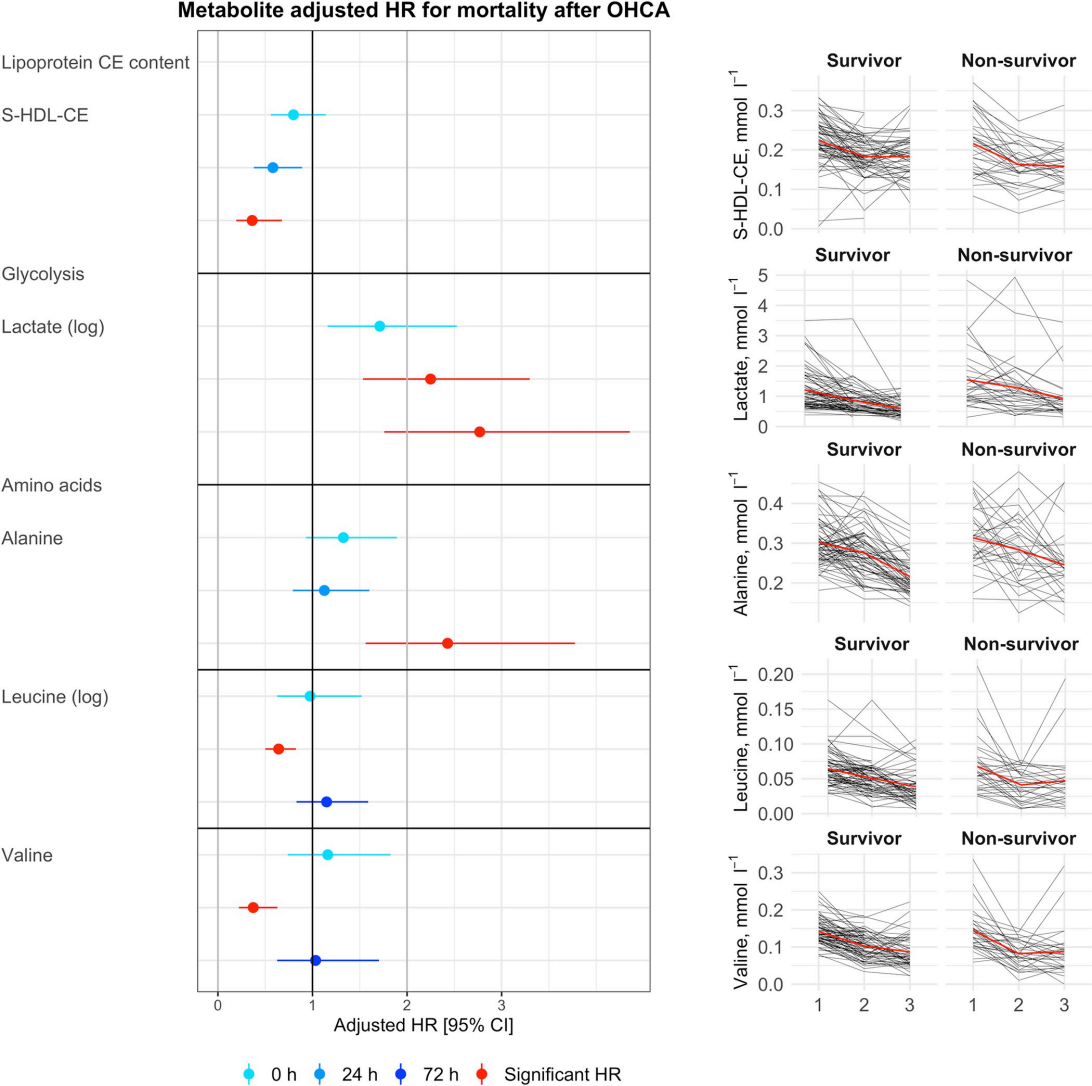
Summary of metabolites with statistically significant association with mortality at 6 months. Forest plots summarising all metabolites with statistically significant adjusted hazard ratios for mortality at 6 months, at one or more time points (left). Line graphs depict individual metabolite concentrations within each patient grouped by survival (right). In forest plots (left), data are reported as an adjusted hazard ratio [95% CI]. Statistically significant results after correction for multiple testing are highlighted in red. Logarithmic transformation was carried out for skewed metabolites, these metabolites are marked with (log). In the line graphs (right), data are reported as measured metabolite concentration in mmol l^-1^, time point means are depicted as red lines. Abbreviations: Ala, alanine; HR, hazard ratio; Lac, lactate; Leu, leucine: OHCA, out-of-hospital cardiac arrest; S-HDL-CE, small high-density lipoprotein cholesteryl ester content; Val, valine.

At baseline upon admission, no metabolites displayed statistically significant associations with 6-month mortality after correction for multiple testing. At 24 h, with respect to the 6-month mortality, it was observed that increased levels of lactate showed a significant adjusted hazard ratio [95% CI], 2.25, 95% CI [1.53; 3.30], p<0.001, as did decreased amounts of the BCAAs leucine 0.64 [0.5; 0.82], p = 0.007 and valine 0.37 [0.22; 0.63], p = 0.003. At 72 h, elevated lactate levels were associated with 6-month mortality i.e. increasing the adjusted hazard ratio to 2.77 [1.76; 4.36], p<0.001. Similarly, at 72 h, high alanine concentrations showed a significant adjusted hazard ratio of 2.43 [1.56; 3.78], p = 0.001, as did decreased S-HDL-CE 0.36 [0.19; 0.68], p = 0.021 (Fig 1). The values of all measured metabolites are shown in S2 Table.

**Fig 2:**
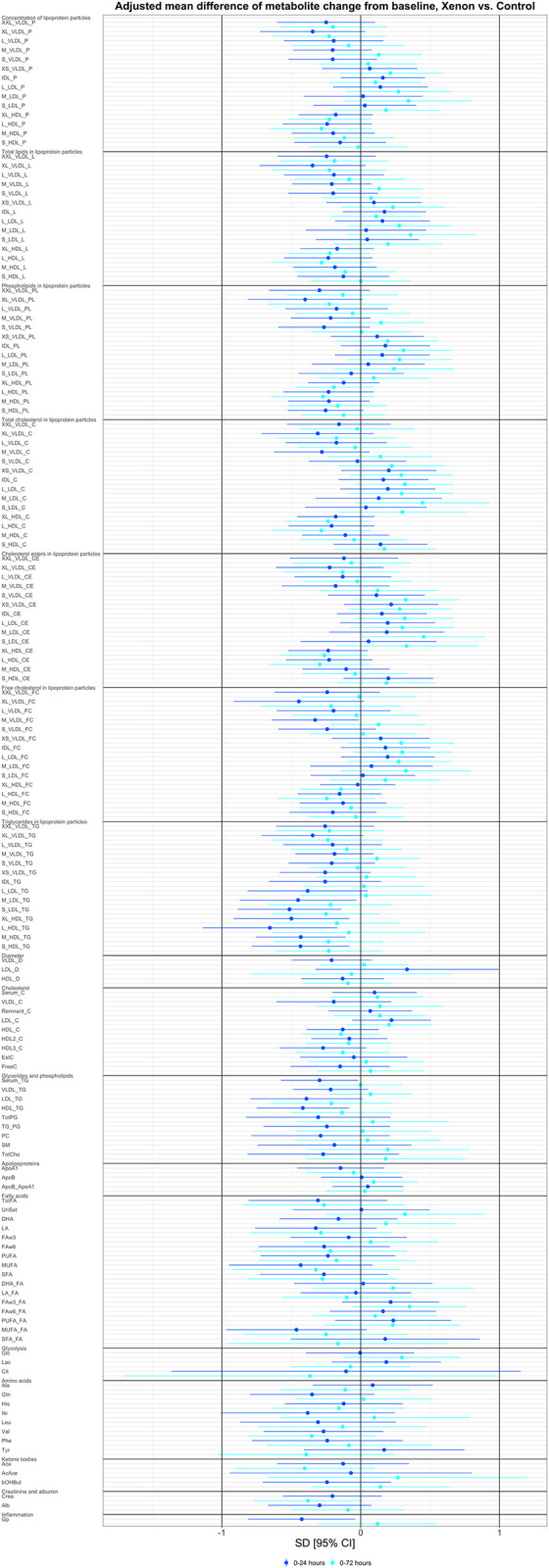
Comparison of all measured metabolites, xenon *vs*. control. A forest plot depicting the intergroup comparison of xenon *vs*. control. There was no statistically significant difference between groups. Data reported as adjusted mean difference of metabolite change from baseline to 24 and 72 hours (SD), 95% confidence interval. Negative values indicate, that the change in metabolite concentration from baseline was lower in xenon treated patients *vs*. the change observed in control group. Abbreviations can be found at S1 Table.

After correction for multiple testing, there were no significant mean differences in metabolites between the xenon and control groups (Fig 2). For the aforementioned metabolites for which a statistically significant association was detected with mortality, xenon *vs*. control adjusted mean difference of metabolite change from baseline are shown in Table 2. For all measured metabolites, these changes can be found in S3 Table.

**Table 2:** Xenon *vs*. Control metabolite difference in selected metabolites.

Metabolite	Hours from admission	Adjusted mean difference of metabolite change from baseline (SD)	95%-CI	Unadjusted p-value	Adjusted p-value
**Cholesterol esters in lipoprotein particles**					
S-HDL-CE	0	-0.10	[-0.43, 0.23]	0.544	1
	24	0.20	[-0.12, 0.52]	0.225	1
	72	0.19	[-0.16, 0.54]	0.297	1
**Glycolysis related**					
Lactate	0	0.17	[-0.20, 0.55]	0.369	1
	24	0.18	[-0.21, 0.58]	0.356	1
	72	-0.07	[-0.50, 0.35]	0.736	1
**Amino acids**					
Alanine	0	0.03	[-0.35, 0.42]	0.863	1
	24	0.09	[-0.34, 0.52]	0.692	1
	72	-0.11	[-0.58, 0.36]	0.639	1
Leucine	0	0.34	[-0.13, 0.82]	0.154	1
	24	-0.31	[-0.87, 0.25]	0.281	1
	72	-0.13	[-0.73, 0.47]	0.671	1
Valine	0	0.49	[0.16, 0.83]	0.004	0.058
	24	-0.27	[-0.70, 0.16]	0.222	1
	72	-0.35	[-0.82, 0.11]	0.137	1

No statistical significance was observed between xenon vs. control. The metabolites with statistically significant association with mortality were selected here as exemplars. Data reported as adjusted mean difference of metabolite change from baseline (SD), 95% confidence interval (95%-CI), unadjusted p-value and adjusted p-value. Negative values indicate smaller means in xenon *vs*. control group. Based on principal component analysis (over 95% of variation in metabolomic data was explained by 14 components in all three time points), the p-values have been adjusted by a factor of 14 for multiple testing, an alpha threshold of 0.05 was used. S-HDL-CE, small high-density lipoprotein cholesteryl ester content.

## Discussion

In this study, the NMR spectroscopy–defined increased lactate at 24 and 72 h and alanine at 72 h, decreased BCAAs leucine and valine at 24 h and S-HDL-CE at 72 h associated with increased 6-month mortality in OHCA patients treated either with TTM in combination with inhaled xenon, or TTM alone. However, no associations were observed in metabolic variables measured upon admission. No significant differences between the xenon and control groups were observed.

In a recent targeted metabolomics analysis of OHCA patients, Beske et al. observed that within 2–3 hours after OHCA, most of the variation between survivors and non-survivors was explained by metabolites of the tricarboxylic acid (TCA)–cycle i.e. malate, fumarate and succinate. This was thought to reflect the initial OHCA induced global ischaemia. There were also other changes noted; the concentrations of lactate, amino acids (e.g. alanine, tyrosine, phenylalanine) and acyl carnitines were elevated in non-survivors, while linoleic acid markers were decreased. The concentrations of isoleucine and leucine were significantly lower in OHCA survivors *vs*. non-survivors, change in valine did not reach statistical significance. However, metabolites were only analysed upon hospital admission [22]. It is worth highlighting that the metabolic profile in the current study was not focused on TCA cycle intermediates as the only TCA cycle intermediate quantified was citrate. Similarly, in contrast with the previous literature neither creatinine nor albumin showed an association with mortality upon hospital admission [23,24]. In the present study, likely due to the smaller sample size, no significant changes upon hospital admission were observed.

In the current study, the increased alanine level was associated with increased mortality. This is likely a consequence of changes in lactate metabolism as alanine levels are metabolically connected to lactate. However, in rat liver perfused with high levels of lactate and pyruvate, a reduction in hepatic uptake of alanine was observed while that of leucine and valine were relatively unaffected [25–27].

The current observation on lactate is in line with prior findings as lactate clearance has been associated with the outcome of patients with ROSC after OHCA [13,28]. Notably, in the current study, the lactate level upon admission showed no association, while the relatively modest elevation of lactate persisting at 24 and 72 h was associated with mortality. Current findings suggest that, rather than the initial OHCA-induced transient global hypoperfusion and ischaemia, the subsequent post-cardiac arrest syndrome related pathophysiological processes may contribute to the persistent lactatemia and the observed association with mortality.

Branched chain amino acids (BCAAs) leucine, valine and isoleucine, are essential amino acids and have a central supporting role on cellular bioenergetics via the tricarboxylic acid cycle (TCA). Leucine is a precursor for acetyl-coenzyme A (CoA) fueling the TCA cycle at citrate, whereas valine has the potential to replenish the TCA cycle intermediate succinyl-CoA, a precursor of succinate. As discussed earlier, higher amounts of the TCA metabolites citrate, 2-oxoglutaric acid, succinate and the subsequent TCA metabolites, fumarate and malate were associated with mortality in OHCA patients upon hospital admission [22]. Moreover, the BCAAs can cross the blood-brain barrier (BBB) and influence brain neurotransmitter synthesis [29].

In the present material, decreased levels of two BCAAs valine and leucine at 24 h, were associated with an increased risk of mortality at 6 months in OHCA patients. The current finding on valine is in line with previous literature in OHCA patients undergoing TTM, as higher valine levels at 48 hours after OHCA were associated with a favourable outcome [30]. However, these results are in contrast with a previous finding in patients with STEMI treated with PCI, where high levels of circulating BCAAs were correlated with in-hospital adverse events: cardiovascular mortality and acute heart failure. This observation was thought to originate from an abnormal catabolism of the BCAAs in the pathologically stressed myocardium evoking mitochondrial dysfunction, superoxide accumulation and cardiomyocyte death [31]. In the present material, STEMI was diagnosed in 30.8% of patients in the xenon group and 35.9% in the control group. The incidence of cardiovascular disease was high (Table 1). Therefore, the underlying metabolic processes reflected by alterations in BCAAs in STEMI and TTM treated OHCA patients may be quite different. It should be noted that BCAA metabolism is intertwined with two metabolic processes of particular interest in the context of OHCA and TTM.

First, BCAA metabolism is connected to thermogenesis via brown adipose tissue (BAT). This may be of interest, as subjects did receive TTM at a target temperature of 33°C. Although the triglyceride content of BAT serves as the primary fuel for BAT thermogenesis, this is depleted rapidly upon cold exposure both in humans and rodents [32,33]. Indeed, it is known that cold-exposure increased thermogenic utilisation of BCAAs in BAT, reducing circulating serum levels of BCAAs in human subjects [34]. Moreover, intravenous amino acid supplementation prior to anaesthesia was able to reduce anaesthesia-induced hypothermia and shivering, supporting the role of BCAA in thermoregulation [35,36]. Previous research in 146 OHCA patients randomised to receive TTM at either 33°C or 36°C revealed that the levels of valine and isoleucine decreased more in response to TTM at 33°C [30]. In contrast, a previous study reported no significant difference in mortality in TTM at 33°C *vs*. TTM with a normothermic target [5]. However, the connection of BCAAs to thermogenesis does not offer a clear explanation to the observed association with reduced mortality suggested by our data.

Second, the metabolic connections of alanine, valine and leucine in the CNS with the metabolism of the neurotransmitter glutamate have demonstrated in multiple ^15^N labelling studies [37–41]. After successful resuscitation, post-cardiac arrest brain injury is the most likely cause for mortality in OHCA patients [42]. One of the underlying mechanisms is thought to be glutamate excitotoxicity, where a bioenergetic failure leads to rapid and extensive vesicular release and inhibited reuptake of the excitatory amino acid glutamate, triggering a destructive cascade of events ultimately leading to cell death [43]. The metabolic fates of glutamate include recycling via the glutamate-glutamine cycle and oxidation in the TCA cycle. Even though both routes were observed to be active in response to the rise in glutamate concentration *in vitro*, the latter route was favoured [44]. Furthermore, it was reported previously that a significant proportion of the ^13^C label from glutamate was detected as lactate *in vitro*, suggesting the existence of TCA mediated metabolism of glutamate via α-ketoglutarate to pyruvate and eventually to lactate, possibly to support the neuronal energy demand [45–47]. Under physiological conditions, it has been hypothesised that this lactate may be exported outside the CNS [48].

Indeed, there seems to be a connection with the circulating BCAA concentration and the magnitude of neuronal damage. Metabolic profiling has identified reduced plasma levels of the BCAAs valine, isoleucine and leucine not only in animal models but also in patients suffering a cardioembolic stroke. Furthermore, the lower BCAA levels correlated with poor neurological outcome in these patients as measured by the modified Rankin Scale [49]. Similar findings have been reported in patients after a traumatic brain injury [50]. Importantly, even though this link between the BCAAs and neuronal damage seems interesting, previous research in animal models under normal physiological conditions has shown that approximately 86% of BCAA catabolism occurs in skeletal muscle, brown fat or liver with the contributions of brain, lung and white fat being relatively modest [51]. However, this may not be the case in the context of neuronal damage.

In the light of the previous literature, it seems tempting to consider the possibility that these current preliminary observations on the concentrations of the BCAAs, alanine as well as lactate, might reflect OHCA-induced neuronal damage. However, further research will be needed to answer this question.

Xenon has already been recognised to be close to the “ideal anaesthetic” because it is not metabolised, it possesses very rapid induction and recovery characteristics, and is remarkably safe and efficient in a variety of clinical settings [7]. In earlier clinical trials with cardiovascular surgical patients, xenon anaesthesia has been characterised as conferring cardiovascular stability accompanied by an inotrope-sparing effect [11,52–57]. Similarly, cardiovascular performance was not compromised by xenon anaesthesia in patients with impaired left ventricular function. In this respect, xenon appeared superior to propofol as an ICU sedative in this high-risk group [58]. Current results further support this because xenon had no significant effect on the investigated metabolic profile. This is an important new characteristic for xenon and could be beneficial in prolonged sedation of compromised cardiovascular and neurological ICU patients, and in patients with multiorgan failure. Therefore, the potential of xenon as an ideal sedative for ICU patients warrants further investigations.

Lastly, increased S-HDL-CE at 72 h was associated with decreased mortality at 6-months. In a process called reverse cholesterol transport (RCT), the initial uptake of cell cholesterol by HDL lipoproteins is followed by esterification by lecithin-cholesterol acyltransferase (LCAT), producing HDL cholesteryl esters. RCT on its own seems an insufficient mechanism to explain the observed association with mortality within 6 months. It has been reported that the levels of lipoprotein metabolites including HDL decline in the days following myocardial injury, and this correlates with the infarct size [59]. This might be one of the contributing factors to the observed association between S-HDL-CE and mortality.

A few limitations need to be discussed. First, there was no control group without TTM. This limits our ability to assess whether the observed association with BCAAs and mortality is connected to TTM. Second, as discussed previously, the metabolic markers sharing metabolic routes are to some extent correlated [60]. Third, the circulating NMR metabolic profile is an indicator of the metabolic state of the whole body, direct conclusions on the origin of the observed metabolic signals. Lastly, possibly due to smaller sample size, we did not detect any changes in metabolite levels upon admission, a finding in contrast with previous research [22–24].

## Conclusion

In conclusion, in OHCA patients randomised to TTM at 33°C alone or in combination with xenon, NMR metabolic profiling at 24 and 72 h demonstrated that lactate, alanine, BCAAs leucine and valine, and S-HDL-CE associate with 6-month mortality. Xenon did not significantly alter the measured metabolic profile, which may be a beneficial attribute considering sedation of compromised ICU patients.

## Supporting information

S1 Data (XLSX)

S1 File (PDF)

S1 Fig CONSORT flow diagram. (DOCX)

S1 Table Metabolite abbreviations. (DOCX)

S2 Table Metabolite associations with 6-month mortality, all metabolites. (DOCX)

S3 Table Xenon *vs*. control, all metabolite differences pages. (DOCX)

## Abbreviations

α-KG: α-ketoglutarate
AAT: aspartate aminotransferase
AcCoA: acetyl coenzyme-A
ADP: adenosine diphosphate
Ala: alanine
ALAT: alanine aminotransferase
Asp: aspartate
ATP: adenosine triphosphate
BAT: brown adipose tissue
BBB: blood brain barrier
BCAAs: branched chain amino acids
BCAT: branched chain amino acid amino transferase
BCKA: branched chain α-ketoacids
BCKDH: branched-chain α-ketoacid dehydrogenase complex
CoA: coenzyme A; EMS, emergency medical service
GDH: glutamate dehydrogenase
Gln: glutamine
Glu: glutamate
GS: glutamine synthase
hBCATc: human branched chain amino acid aminotransferase, cytocolic
hBCATm: human branched chain amino acid aminotransferase, mitochondrial
HR: hazard ratio
ICU: intensive care unit
Lac: lactate
LCAT: lecithin-cholesterol acyltransferase
LDH: lactate dehydrogenase
Leu: leucine
NAD(P)+: dihydronicotinamide-adenine dinucleotide phosphate
NAD(P)H: reduced dihydronicotinamide-adenine dinucleotide phosphate
NH4+: ammonium ion
NMR: nuclear magnetic resonance spectroscopy
MRI: magnetic resonance imaging
OAA: oxaloacetate
OHCA: Out-of-hospital cardiac arrest
PAG: phosphate activated glutaminase
PDH: pyruvate dehydrogenase
PEA: pulseless electrical activity
Pyr: pyruvate
RCT: reverse cholesterol transport
ROSC: return of spontaneous circulation
SAH: subarachnoid haemorrhage
S-HDL-CE: small high-density lipoprotein cholesterol ester content
SuCoA: succinyl coenzyme-A
TCA: tricarboxylic acid cycle
TTM: Targeted temperature management; Val, valine
