# Supplementary material for: A metabolic profile of xenon and metabolite associations with 6-month mortality after out-of-hospital cardiac arrest: A post-hoc study of the randomised Xe-Hypotheca trial
Source: PLoS One. 2024 Jun 4;19(6):e0304966. doi: 10.1371/journal.pone.0304966 (PMC11149864; doi:10.1371/journal.pone.0304966)
Supplement: S1 File — (PDF) [file pone.0304966.s002.pdf]

Supplementary Online Content

Laitio R, Hynninen M, Arola O, et al. Effect of inhaled xenon on cerebral white matter damage in comatose survivors of out-of-hospital cardiac arrest: a randomized clinical trial. *JAMA*. doi:10.1001/jama.2016.1933

Trial protocol

This supplementary material has been provided by the authors to give readers additional information about their work.

CLINICAL STUDY PROTOCOL

**Study title:**	Effect of Xenon, in Combination with Therapeutic Hypothermia, on the Brain and on Neurological Outcome following Brain Ischemia in Cardiac Arrest Patients (EudraCT number 2009-009505-25)
**Date:**	24/02/2014
**Version:**	8
**Study Code:**	Xe-Hypotheca
**Study drug:**	Xenon
**Phase:**	II

This study will be conducted in accordance with GCP.

Principal Investigator: Timo Laitio, M.D. Ph.D
Contact address: Perioperative Services, Intensive Care Medicine and Pain Management, Turku University Hospital, University of Turku, P.O.X. 52, FIN-20521, Turku, Finland

Institutes: Perioperative Services, Intensive Care Medicine and Pain Management, Turku University Hospital, University of Turku, P.O.X. 52, FIN-20521, Turku, Finland

Sponsor: An investigator-initiated clinical drug study

SIGNATURES

Approved by:

Principal investigator: ____________________________ Date: ___/___/___
Timo Laitio

Biostatistician: ____________________________ Date: ___/___/___
TeroVahlberg

LIST OF INVESTIGATORS AND CONTACT INFORMATION

Turku University Hospital (Amendment 6.1 see Summary)

Juhani Airaksinen	Professor, Chief physician, Cardiological Unit, Department of Internal Medicine, Turku University Hospital, POB 52, 20521 Turku; e-mail: juhani.airaksinen@tyks.fi; tel. 02-3130005
Olli Arola	Staff physician, EDIC,Specialist for internal medicine Department of Anesthesiology, Intensive Care, Emergency Care and Pain Medicine, Turku University Hospital, POB 52, 20521 Turku; e-mail: olli.arola@tyks.fi; 02-3130499
Olli Eskola	Chemist, Turku PET Centre Turku PET centre, Turku University Hospital, POB 52, 20521; e-mail; olli.eskola@abo.fi; tel. 02-3335042 and 040-5689757
Juha Grönlund	Staff Intensivist, M.D., PhD, EDIC, Department of Anesthesiology, Intensive Care, Emergency Care and Pain Medicine, Turku University Hospital, POB 52, 20521 Turku; e-mail: juha.gronlund@tyks.fi
Sarita Forsback	Chemist, Turku PET Centre Turku PET centre, Turku University Hospital, POB 52, 20521; e-mail; sarita.forsback@abo.fi; tel. 3133552 and 050-5444807,
Outi Inkinen	Staff physician, Intensive Care Unit, Deprtment of Internal Medicine, Turku University Hospital, POB 52, 20521 Turku; e-mail: outi.inkinen@tyks.fi; tel. 02-3130510
Timo Laitio	M.D, PhD, Staff cardiac anaesthesiologist, Department of Anesthesiology, Intensive Care, Emergency Care and Pain Medicine, Turku University Hospital, POB 52, 20521 Turku; e-mail: timo.laitio@tyks.fi and timo.laitio@elisanet.fi; tel. 02-3130979
Ruut Laitio	M.D, PhD, Staff anaesthesiologist Department of Anesthesiology, Intensive Care, Emergency Care and Pain Medicine, Turku University Hospital, POB 52, 20521 Turku; e-mail: ruut.laitio@tyks.fi; tel. 02-3130606
Jyrki Liesivuori	Professor, Finnish Institute of occupational health and Department of Pharmacy, Drug Development and Therapeutics, University of Turku, Lemminkäisenkatu 14-18B, FI-20520, Turku, Finland; e-mail:jyrki.liesivuori@ttl.fi; tel:
Mervyn Maze	Professor, Department of Anesthesia and Perioperative Care, University of California San Francisco, USA
Heikki Minn	Acting Professor in PET Centre and Associate Professor in Clinical Oncology, Turku PET centre, Turku University Hospital, POB 52, 20521 Turku; e-mail: heikki.minn@tyks.fi; tel. 02-3130149

Klaus Olkkola	Professor and Chairman Department of Anesthesiology, Intensive Care, Emergency Care and Pain Medicine, POB 52, 20521 Turku University Hospital, Turku; e-mail: klaus.olkkola@tyks.fi and Klaus.olkkola@utu.fi; tel. 02-3131960
Riitta Parkkola	Associate Professor, Neuroradiologist, Department of Radiology, Turku University Hospital, POB 52, 20521 Turku; e-mail: riitta.parkkola@tyks.fi; tel. 02-3130148
Sami Virtanen	Neuroradiologist. PhD student, IRad Oy, Turku. **(Amendment 3.3 see Summary)**
Mikko Pietilä	Staff cardiologist, M.D, PhD, Cardiological Unit, Department of Internal Medicine, Turku University Hospital, POB 52, 20521 Turku; e-mail: mikko.pietila@tyks.fi; tel. 02-3130511
Juha Rinne	Professor of neurotransmission, Vice-director of Turku PET Centre Turku PET centre, Turku University Hospital, POB 52, 20521 Turku; e-mail: juha.rinne@tyks.fi; tel. 02-3131866
Eero Rissanen	M.D., PhD, PET Centre Turku PET centre, Turku University Hospital, POB 52, 20521 Turku; e-mail: eero.rissanen@tyks.fi; tel.02-3131879
Risto O. Roine	Professor, Chief Physician, Department of Neurology, Turku University Hospital, POB 52, 20521 Turku; e-mail: risto.roine@tyks.fi and risto.roine@luukku.com; tel. 02-3130758
Timo Roine	iMinds-Vision Lab, Department of Physics, University of Antwerp, Belgium; University of Antwerp (CDE) Universiteitsplein 1, N.1.16 B-2610 Wilrijk, Belgium; timo.roine@uantwerpen.be
Antti Saraste	M.D., PhD, Cardiologist, Turku PET Centre and Cardiology, Turku University Hospital; Turku University Hospital, POB 52, 20521 Turku; e-mail: antti.saraste@tyks.fi; tel. 040-7022756.
Jani Saunavaara	PhD, MRI Physicist, Department of Radiology, Turku University Hospital, POB 52, 20521 Turku; e-mail: riitta.parkkola@tyks.fi; tel. 02-3130148 **(Amendment 2.7 see Summary)**
Harry Scheinin	Associate Professor, Department of Pharmacology, University of Turku, Kiinanmyllynkatu 10, 20014 Turku University; harry.scheinin@utu.fi; tel. +358400825599
Olof Solin	Professor of Radiochemistry, University of Turku, Turku PET centre, Turku University Hospital, POB 52, 20521 Turku; e-mail: olof.solin@abo.fi; tel. 040-5081485

Emmi Ylikoski	M.D. PhD, Staff anaesthesiologist, Department of Anesthesiology, Intensive Care, Emergency Care and Pain Medicine, Turku University Hospital, POB 52, 20521 Turku; e-mail: emmi.ylikoski@tyks.fi;
Tero Vahlberg	Head of the Department of Biostatistics, University of Turku, Lemminkäisenkatu 1, 20014 Turku University; e-mail: tero.wahlberg@utu.fi; tel. 02-3338443
Research sites	Intensive Care Unit, Department of Anesthesiology, Intensive Care, Emergency Care and Pain Medicine, Turku University Hospital, Turku; tel. 02-3130950
	Turku PET centre, Turku University Hospital, POB 52, 20521 Turku
	Department of Radiology, Turku University Hospital, POB 52, 20521 Turku
Laboratory	TYKSLAB, POB 52, 20521 Turku
Monitoring	Lena Nybond, Director, Turku CRC, PL 52, 20521 Turku; Tel. (02) 313 3588, 050 438 3707, e-mail: turkucrc@tyks.fi and lena.nybond@tyks.fi
Safety Committee	Organized by Turku CRC

Helsinki University Hospital (Amendment 5.1 see Summary)

Marja Hynninen	Director of the Intensive Care Unit (Teho 20), Meilahti, PL 340, 00029 HUS, associate professor, M.D., PhD, marja.hynninen@hus.fi, tel.0504271731
Johanna Wennervirta	M.D., PhD, Staff intensivist, Teho20 Meilahti, PL340, 00029 HUS johanna.wennervirta@hus.fi; tel. 050 4271724
Minna Bäcklund	M.D., PhD, Staff intensivist, Teho20 Meilahti, PL340, 00029 HUS minna.backlund@hus.fi, tel. 050 4271740
Päivi Silvasti	M.D., Staff intensivist, Jorvin Sairaalan Teho, PL 800, 00029 HUS paivi.silvasti@hus.fi; tel. 050 4286274
Eija Nukarinen	M.D., Staff intensivist, Teho20 Meilahti, PL 340, 00029 HUS eija.nukarinen@hus.fi; tel.
Marjaana Tiainen	M.D., PhD, Staff neurologist, Department of Neurology, PL340, 00029 HUS, tel. 09-47172662 marjaana.tiainen@hus.fi
Leena Valanne	Associate professor, PhD, M.D. HUS Röntgen, Department of Neuroradiology; leena.valanne@hus.fi
Juha Martola	PhD, M.D., Staff neuroradiologist, HUS Röntgen, Department of Neuroradiology; tel.
Heli Silvennoinen	PhD, M.D., Staff neuroradiologist, HUS Röntgen, Department of Neuroradiology; tel.
Veli-Pekka Harjola	Department of Cardiology, PL 800, 00029 HUS; veli-pekka.harjola@hus.fi
Jussi Niiranen	Department of Cardiology, PL 800, 00029 HUS; jussi.niiranen@hus.fi

Kirsi Korpi Department of Cardiology, PL 800, 00029 HUS; kirsi.korpi@hus.fi
Marjut Varpula Department of Cardiology, PL 800, 00029 HUS; marjut.varpula@hus.fi
Laboratory HUSLAB PL340, 00029 HUS
Monitoring Satu Nikander, Study Coordinator, tel.+358 40 847 0008; e-mail: ext-satu.nikander@hus.fi

Safety Board Chair Päivi Rautava, Turku CRC

TABLE OF CONTENTS

LIST OF INVESTIGATORS AND CONTACT INFORMATION 3

TABLE OF CONTENTS 7

SYNOPSIS 9

1 **INTRODUCTION 12**

- 1.1 Hypothermia treatment – State-of-the-Art 12
- 1.2 Xenon and neuroprotection 12
- 1.3 Xenon and hypothermia 13
- 1.4 Xenon's clinical applicability 14
- 1.5 Rationale of the study 14

2 **OBJECTIVES AND PURPOSE 15**

- 2.1 Detailed objectives 15
- 2.2 Hypotheses 15

3 **STUDY DESIGN 16**

- 3.1 Type and design of the study 16
- 3.2 Randomisation and blinding 16
- 3.3 General study outline and study schedule 16

4 **SUBJECT SELECTION 20**

- 4.1 Source population 20
- 4.2 Number of patients 20
- 4.3 Inclusion criteria 20
- 4.4 Exclusion criteria 20
- 4.5 Subject withdrawal/replacement 21

5 **STUDY TREATMENTS 21**

- 5.1 Control and study treatment 21
- 5.2 Administration of study treatment 22
- 5.3 Handling of study products 22
- 5.4 Prior and concomitant treatments 23
  - 5.4.1 Sedation and analgesia 23
  - 5.4.2 Mechanical ventilation 23
  - 5.4.3 Hemodynamics and other 23
- 5.5 Treatment compliance 24
- 5.6 Criteria for premature study termination 24

6 **ASSESSMENTS 25**

- 6.1 Assessment of efficacy 25
  - 6.1.1 Echocardiography 25
  - 6.1.2 Magnetic resonance imaging of the brain 26
  - 6.1.3 PET imaging of the brain (not to be performed in Helsinki) 28
  - 6.1.4 Neurological evaluation 30
  - 6.1.5 Long-term outcome 30
  - 6.1.6 Biochemical assessments 30
  - 6.1.7 Transcranial Doppler (not to be performed in Helsinki) 31
  - 6.1.8 Cerebral carbon dioxide reactivity test (not to be performed in Helsinki) 31
  - 6.1.9 Heart rate variability 32
  - 6.1.10 Esophagus pressure measurements 32
- 6.2 Assessment of safety 33
- 6.3 Other assessment 34
- 6.4 Primary and secondary variables 34

7 **ADVERSE EVENTS** 35
7.1 DEFINITIONS 35
7.2 REPORTING OF ADVERSE EVENTS (AES) 36
7.3 SAE REPORTING 36
7.4 TREATMENT OF EMERGENCIES 37
8 **STATISTICS** 37
8.1 STATISTICAL HYPOTHESIS 37
8.2 SAMPLE SIZE 37
8.3 STATISTICAL PLAN AND ANALYSIS 37
8.4 INTERIM ANALYSES AND STOPPING RULES 37
9 **QUALITY CONROL AND QUALITY ASSURANCE** 38
9.1 INFORMATION OF STUDY PERSONNEL AND TRAINING 38
9.2 MONITORING 38
9.3 PROTOCOL AMENDMENTS 38
10 **DATA HANDLING AND RECORD KEEPING** 38
10.1 CASE REPORT FORMS 39
10.2 ELECTRONIC DATA COLLECTION AND CAPTURE 39
10.3 DATA MANAGEMENT 39
10.4 STUDY SUBJECT REGISTER 40
11 **ETHICS** 40
11.1 ETHICAL REVIEW 40
11.2 ETHICAL CONDUCT OF THE STUDY 40
11.3 SUBJECT INFORMATION AND INFORMED CONSENT 41
12 **FINANCING AND INSURANCE** 41
13 **STUDY REPORT AND PUBLICATION(S)** 41
14 **ARCHIVING** 41
15 **STUDY AGREEMENT** 41
16 **REFERENCES** 41
17 **ABBREVIATIONS AND DEFINITION OF TERMS** 43
18 **SUMMARY OF THE AMENDMENTS** 44

SYNOPSIS

**Name of the Sponsor:** An investigator-initiated clinical drug study		
**Name of the finished product:** LENOXe™		
**Name of the active ingredient:** Xenon (Xe)		

**Study title:** Effect of Xenon, in Combination with Therapeutic Hypothermia, on the Brain and on Neurological Outcome following Brain Ischemia in Cardiac Arrest Patients
**Study code:** Xe-Hypotheca
**Investigators and study centres:** Timo Laitio (PI), Juhani Airaksinen, Olli Arola, Olli Eskola, Juha Grönlund, Sarita Forsback, Outi Inkinen, Ruut Laitio, Jyrki Liesivuori, Mervyn Maze, Heikki Minn, Klaus Olkkola, Riitta Parkkola, Jani Saunavaara, Sami Virtanen, Antti Saraste, Mikko Pietilä, Juha Rinne, Risto Roine, Harry Scheinin, Olof Solin, Tero Vahlberg, Emmi Ylikoslki,; Department of Anaesthesiology, Intensive Care, Emergency Care and Pain Medicine, Turku PET Centre, Department of Radiology, POB 52, 20521 Turku Turku University Hospital, Turku, Finland<br>**Helsinki:** Marja Hynninen (PI), Johanna Wennervirta, Minna Bäcklund, Päivi Silvasti, Eija Nukarinen, Marjaana Tiainen, Leena Valanne, Juha Murtola, Heli Silvennoinen, Veli-Pekka Harjola, Jussi Niiranen, Marjut Varpula, Kirsi Korpi; Department of Anesthesiology and Intensive Care, Dept. of Neurology, HUS Rontgen, Dept. of Cardiology, Meilahden sairaala, Helsinki University Hospital
**Development phase:** II
**Objectives:**<br>1) To show a significant reduction in the degree of severity of the ischemic brain injury in the study treatment group as compared with the control group;<br>2) To explore the underlying mechanisms for the synergistic neuroprotective interaction of xenon and hypothermia.<br>3) To correlate these findings with neurological outcome to determine surrogate markers of favourable clinical outcome at six months.
**Methodology:** Study design is an open randomized two armed parallel follow-up study. The study treatment or the control treatment will be administered to patients who have suffered a global ischemic brain injury after an out-of-hospital cardiac arrest. Various magnetic resonance and PET imaging techniques of the brain will be undertaken to evaluate the effects of the treatments on cerebral hypoxia, neuronal loss and mitochondrial dysfunction. Neurological outcome will be evaluated at three and at six months after cardiac arrest.
**Sample size:** 110 out-of-hospital cardiac arrest patients; based on a power analysis

Main criteria for inclusion:

Inclusion criteria:

1. Ventricular fibrillation or non-perfusive ventricular tachycardia as initial cardiac rhythm.
2. The 1st attempt at resuscitation by emergency medical personnel must appear within 15 minutes after the collapse.
3. The cause for collapse should be considered primary as cardiogenic and the return of spontaneous circulation (ROSC) should have been gained in 45 minutes after the collapse
4. Patient should be still unconscious in the emergency room.
5. Age: 18 – 80 years
6. Obtained consent within 4 hours after arrival to the hospital

Exclusion criteria:

1. Hypothermia (< 30°C core temperature)
2. Unconsciousness before cardiac arrest (cerebral trauma, spontaneous cerebral haemorrhrages, intoxications etc.)
3. Response to verbal commands after the return of spontaneous circulation and before randomization
4. Pregnancy
5. Coagulopathy
6. Terminal phase of a chronic disease
7. Systolic arterial pressure < 80 mmHg or mean arterial pressure < 60 mmHg for over 30 min period after ROSC
8. Evidence of hypoxemia (arterial oxygen saturation < 85%) for > 15 minutes after ROSC and before randomization.
9. Factors making participation in follow-up unlikely
10. Enrolment in another study

Investigational drug/treatment, dose and mode of administration: 40% concentration of inhaled xenon in oxygen/air combined with mild hypothermia treatment (MHT) with target core temperature of 33-34 ºC.

Comparative drug(s)/placebo/treatment, dose and mode of administration: MHT with target core temperature of 33-34 ºC. MHT is defined as the time of maintaining target core temperature.

Duration of treatment: MHT duration is 24 hours. Xenon treatment may start before or after the target core temperature has been achieved, but it must start within 4 hours after arrival to the hospital. Xenon treatment will be continued until completion of MHT. MHT is followed by a controlled rewarming (0.5 ºC/hour).

Assessments:

Assessments of efficacy:

1. *First magnetic resonance imaging (MRI) of the brain will be performed within 16 hours after completion of rewarming, the 2nd MRI 10±2 days and the 3rd 3-3.5 years after OHCA*: **(Amendment 6.2 and 6.3 see Summary)**
   a) Brain tissue anatomy: T2, FLAIR and 3DT1 images
   b) Depth and extent of the ischemia: Mean diffucivity value of diffusion tensor proton magnetic resonance imaging (DTI)
   c) White matter tract degeneration of the corresponding tracts of the infarcted area: Fractional Anisotrophy value of DTI
   d) Extent of hypoperfusion of the brain tissue: Perfusion MRI Data

e) Cerebral metabolic dysfunction: Proton magnetic resonance spectroscopy
f) Volumetric changes of the gray matter, white matter, and cerebro-spinal fluid spaces: Advanced post-processing method
g) Estimation of hemosiderin and local neurodegeneration: Susceptibility-weighted imaging (SWI), will be used only in the 3rd MRI **(Amendment 6.4 see Summary)**

2. Brain *PET will be performed 5±2 days after OHCA to image microglia cell activation* by using C11-PK11195 PET positron emission tomography (PET).*(not to be performed in Helsinki)*

3. Autonomic nervous system: Heart rate variability and baroreceptor function;
4. Cardiac function: Transthoracic echocardiography will be performed before, during and after treatments in all feasible patients.
5. *Biochemical assessment*: cardiac enzyme release (P-TnT, P-Ck-Mb), neuron specific enolase (P-NSE), D-lactate and plasma catecholamines before treatment and at 24h, at 48h and at 72h after OHCA; urine catecholamines during MHT.
6. *Neurological evaluation*: at 3 and 6 months after cardiac arrest
7. Long-term outcome: 3-3.5 years after cardiac arrest **(Amendment 6.5 see Summary)**
Assessment of safety: An independent safety committee and a monitor will be involved throughout the trial. A brain CT-scan is performed for all patients before initiation of any treatments to exclude a possible cerebral origin of the cardiac arrest. The subjects will be continuously monitored according to standard care of patients in ICU (i.e. continuous invasive blood pressure, continuous 2-channel ECG, pulse oximetry, central venous pressure, body temperature, blood gas assessment at least once per hour, continuous end-tidal CO_2 %, ventilatory frequency, tidal volyme, minute volyme, inspiratory O_2 %, end-tidal O_2 % and xenon %). Also, the patients will be treated in accordance with highest standards of intensive care including a routine battery of daily laboratory tests.

Statistical methods: 1) Basic statistical tests (t-tests, Mann-Whitney, Chi square, etc); 2) Survival analysis methods; 3) An analysis of variance for repeated measurements; 4) Linear mixed models
Significance level of 0.05 and an estimation of 95 % confidence intervals will be used in the statistical analyses.

1 INTRODUCTION

1.1 Hypothermia treatment – State-of-the-Art

In the case of sudden cardiac arrest, patients are confronted with a global ischemia to the brain. At a rate of sudden cardiac arrest of approximately 1/1,000 population/year (1), the annual incidence across Europe is approximately 460,000 per year. Several studies using historical controls (2) as well as two randomized controlled trials (3,4) have demonstrated that the use of therapeutic hypothermia following cardiac arrest improves neurological outcome in patients with witnessed arrests. The robustness of these findings is evidenced by the fact that the time to achieve mild hypothermia varied between 2 (4) and 8 (3) hours. The Hypothermia After Cardiac Arrest (HACA) trial reported an absolute increase in rates of favourable neurological outcome of 16% (relative increase 41%) while an absolute increase of 23% (relative increase 88%) was reported in the second study. A meta-analysis concluded that the number needed to treat to achieve one additional patient with good neurological outcome was 6 (5).

In 2005 European Resuscitation Council published Advance Cardiac Life Support guidelines that included the recommendation for the use of hypothermia after out-of-hospital cardiac arrest (OHCA). If cardiac resuscitation is successful, the state-of-the-art is to actively cool these patients into a state of moderate hypothermia for 24 hours in an intensive care unit (ICU). Currently, mortality at six months after cardiac arrest in hypothermia treated patients is 41 % and 55 % in the normothermia treated patients. At six months, 55 % of the hypothermia treated patients and 39 % of the patients treated with normothermia have a favorable neurologic outcome.

Although therapeutic hypothermia does provide a statistically significant improvement in OHCA patients, the benefit is only moderate (5). Even if the hypothermia treatment would be universally adopted within Europe at least 120,000 patients each year would still have an unfavourable neurological outcome associated with unbearable suffering at an individual level and major economical consequences. Thus, strategies designed to increase the efficacy of therapeutic hypothermia are needed.

1.2 Xenon and neuroprotection

A noble gas, xenon (Xe), exists as a monoatomic gas. Although it is referred to as “inert,” xenon’s low ionization potential permits its electron shell to be polarized by surrounding molecules, thereby inducing a dipole that facilitates biologic interactions including binding to proteins (6). Xenon has been shown to be a non-competitive antagonist of the N-methyl-D-aspartate (NMDA) subtype of the glutamate receptor (7). Since Mervyn Maze and his study group discovered the neuroprotective effect of xenon in in vitro studies (8), numerous additional studies have demonstrated the neuroprotective property of xenon in both in vitro and in vivo (i.e. at least in 3 species) models of acute neuronal injury (8-16). Their more recent preclinical studies showed neuroprotective effects of xenon in various in vivo models of acute neuronal injury involving administration of excitotoxins to rats (9), cardiopulmonary bypass (CPB) in rats (9), middle cerebral artery occlusion in mice (10), cardiac arrest in pigs (15), and hypoxic-ischemia in neonatal rats (13,16). In the latest study by Fries et al., cardiac arrest was induced for 24 pigs followed by an 8 minute period without mechanical ventilation and resuscitation. Thereafter, the pigs were resuscitated, and 1 hour after successful resuscitation, they were treated with 30/70 % oxygen/air or 70 % xenon for 1 or 5 hours. In this study with clinically relevant setup, xenon conferred neurohistopathologic protection (e.g.

significantly less perivascular inflammation in the putamen and caudate nucleus and less necrotic neurons in the putamen) and transient improvement in functional outcome.

Recent studies have begun to identify plausible sites of action for xenon's neuroprotective effect. Xenon is a potent activator of the two-pore domain K^+ channel, which has been recently shown to play an important role in neuroprotection (17). Xenon has little effect on the α-amino-3-hydroxy-5-methyl-4-isoxazolepoprionate (AMPA) receptor at physiological concentrations of glutamate (18). At higher glutamate concentrations (as occurs during ischemic injury) xenon blocks both kainate as well as AMPA receptors thereby preventing the depolarization phase of the pathogenic mechanism (19). Xenon also induces the expression of hypoxia inducible factor 1α (HIF 1a) and its downstream effectors erythropoeitin (EPO) and vascular endothelial growth factor (VEGF), both of which can interrupt the apoptotic pathway (20). Trophic factors including brain derived neurotrophic factor (21) and activity dependent neuroprotective protein (ADNP) that are induced by xenon are capable of inhibiting apoptosis and allowing cytoskeletal repair to maintain cellular morphology. Recently, it has been shown that xenon is able to decrease global and regional cerebral metabolic rate of glucose (MRGlu) using [18F]-labeled fluorodeoxyglucose ([18F]FDG) as a tracer for positron emission tomography (PET) in healthy human volunteers (22-24). This distinguishes xenon from other general anesthetics that block the NMDA subtype of the glutamate receptor such as ketamine (25) in which an increase in MRGlu was noted, and N2O (26).

1.3 Xenon and hypothermia

Multiple mechanisms have been identified by which hypothermia protects neurons after ischemia (27). Pathological mechanisms initiated by ishemia and reperfusion such as neuroexitatory cascade (Ca influx, accumulation of glutamate, and a release of its co-agonist glycine), apoptosis, inflammation, metabolic effects, loss of ion gradients, increased free radicals are mitigated by hypothermia. A remarkable efficacy of hypothermia to be able to improve neurological outcome after global ischemia may be a result of its ability to affect multiple systems simultaneously.

Recently, Mervyn Maze with his study group demonstrated in an in vitro study that a combination of xenon and hypothermia causes a synergistic neuroprotective interaction which was confirmed by isobolographic analysis (16). They also showed in vivo that a combination of xenon (20%; an ineffective dose alone) and hypothermia (35 ºC; also ineffective on its own) administered 4 h *after* hypoxic-ischemic injury in neonatal rats provided synergistic neuroprotection assessed by hemispheric weight, by morphological criteria, and by functional neurological studies 30 days after the injury (16). In a more recent in vivo study, injured neonatal rats were exposed to 20% xenon for two hours, starting 3 hours after 90 min of post-injury treatment with hypothermia at 35ºC; this combination of interventions, even when applied asynchronously, still significantly decreased infarct size after hypoxemic-ischemic injury (28). A very recent in vivo study with a model of hypoxic/ischemic brain injury in neonatal rats further confirms the additive neuroprotective effect of the combination (Xe 50%+Hypothermia 32ºC) by showing that the functional improvement was almost complete, was sustained long-term, and was accompanied by greatly improved histopathology (29). The protective mechanism of the combination, in both *in vitro* and *in vivo* models, appears to converge on the apoptotic pathway (16). The prosurvival (anti-apoptotic) protein Bcl-xL is upregulated by the synergistic combination while expression of Bax (the pro-apoptotic factor) is decreased by the combination. It is notable that expression of neither protein is changed by either xenon 20% or hypothermia at 35ºC, i.e. non-protective interventions when administered alone (16). While it appears that the synergistic neuroprotective action of xenon and hypothermia involves the apoptotic pathway proximal to mitochondrial injury, there are other possible targets that can be modulated by the synergistic interaction of xenon and hypothermia, including excitotoxicity, inflammation, and metabolic rate.

1.4 *Xenon's clinical applicability*

Xenon is anesthetic at high concentrations in man (> 60 %), although its neuroprotective concentration is estimated to be about 30% of the concentration required for anesthesia in humans when combined with modest hypothermia. Marketing authorization for the use of xenon as an anesthetic has been obtained in Russia in 2002, in Germany in 2005, and extended through Europe in March 2007. Xenon's anesthetic effect has been known for more than fifty years; its remarkable safety and efficacy in a variety of clinical settings bring it close to the "ideal anesthetic" (30). With a minimal alveolar concentration (MAC) of less than 70% xenon is more potent than nitrous oxide. It has minimal hemodynamic effects and has the lowest solubility (blood/gas partition coefficient of 0.115) of any known anesthetic agent. Thus it possesses very rapid induction and recovery characteristics. The manufacturing costs have been high but research on fully closed delivery systems and novel scavenger devices are making the use of xenon economically feasible. As an elementary substance xenon possesses no environmental disadvantages. Retrieving a drug directly from the atmosphere for use and then allowing it to return back unchanged represents a unique possibility in modern medicine.

In the clinical arena xenon has been used in several thousand anesthesias in Europe and Russia (31) and is associated with remarkable cardiovascular stability (31). Xenon does not affect myocardial contractility (33,34); recent result show that cardiovascular performance is not compromised by xenon anesthesia in patients with impaired left ventricular (LV) function, and in this respect appears superior to propofol for anaesthesia in this high-risk group (35). The benefit of these clinical features may be even more important to patients who have suffered myocardial injury as usually occurs during out-of-hospital cardiac arrest because xenon has been shown to exert myocardial protective properties in a preclinical model of myocardial ischemic injury (36). Prolonged xenon exposure in the critical care setting has been shown to be safe (37). Therefore, xenon has potential to become the sedative of choice in the management of compromised patients in critical care settings (37). Xenon has also been used safely in patients who have suffered an acute stroke (38). A recent report shows that xenon (up to 50% for up to 3h) is safe in patients undergoing cardiac surgery (39). Furthermore, recent studies by Laitio R et al. (23,40) demonstrated that xenon can be delivered safely for several hours at high concentrations (i.e. 65%) to humans; they delivered xenon as a single-agent anesthetic to achieve deep anesthesia in healthy human volunteers (40).

1.5 *Rationale of the study*

Although, therapeutic hypothermia clearly provides a statistically significant improvement in OHCA patients, the benefit is clinically quite modest. Thus, strategies designed to increase the efficacy of therapeutic hypothermia are needed. The following recommendations for standards regarding preclinical neuroprotective and restorative drug development by Stroke Therapy Academic Industry Roundtable (STAIR) have been fulfilled for xenon (Stroke Therapy Academic Industry Roundtable (STAIR). Recommendations for standards regarding preclinical neuroprotective and restorative drug development. *Stroke*. 1999;30:2752-2758).

1. demonstration of efficacy in at least two species, in at least two laboratories that use different models
2. effective in both permanent and transient focal ischaemia
3. improvement in short-term and long-term histological and functional outcomes
4. effective when administered several hours after the onset of ischaemia
5. achieves brain concentrations that rapidly equilibrate with plasma
6. consistent minimum neuroprotective concentration across different species, allowing prediction of the putative neuroprotective concentration in humans

7. sigmoid rather than bell-shaped dose-response curve
8. data should be published or submitted for review in a peer-reviewed journal

Furthermore, current evidence of four separate preclinical and several other studies show xenon's remarkable neuroprotective interaction with hypothermia. In addition, xenon's remarkable safety and efficacy in a variety of clinical settings has been well demonstrated bringing it close to the "ideal anesthetic". Therefore, current proposed clinical trial designed to find out whether the efficacy of hypothermia treatment on neuroprotection can be improved with a combination of xenon inhalation should be justified.

This study will be conducted in accordance with this protocol, GCP, GSP and pertinent laws in Finland relating to clinical trials.

2 OBJECTIVES AND PURPOSE

The main purpose of this study is to explore the underlying mechanisms for the synergistic neuroprotective interaction of xenon and hypothermia in patients suffering cerebral ischemia post cardiac arrest, by undertaking brain imaging to evaluate their effects on cerebral hypoxia, neuronal loss and mitochondrial dysfunction. In addition we aim to correlate these findings with neurological outcome to determine surrogate markers of favourable clinical outcome at six months.

2.1 Detailed objectives

1. To explore whether Xe in combination with MHT has better neuroprotective effect than the MHT alone in OHCA patients by showing a significant reduction in the degree of severity of the ischemic brain injury in the MHT+Xe group compared with the MHT group using diffusion tensor magnetic resonance imaging (DTI). To investigate the presence, pattern and long-term progression of white matter and gray matter degeneration and long-term volumetric changes of the gray matter, white matter, and cerebro-spinal fluid spaces. To define an association of these findings with neurological outcome and mortality**.(Amendment 6.6 see Summary)**
2. To explore the mechanism for neuroprotective interaction of xenon and hypothermia in these patients by using C11-PK11195 PET positron emission tomography (PET) and proton magnetic resonance spectroscopy (^{1}H-MRSI) in the thalamus and striatum; to identify neuroprotective properties of xenon and a possible predictive imaging marker for neurological outcome.
3. To characterize the behaviour of the neuron specific enolase (NSE) in the different treatment groups; to explore whether there is correlation between NSE and brain imaging showing neuronal injury
4. To define the possible cardioprotective property of the combined treatment of xenon and moderate hypothermia in patients after cardiac arrest.

2.2 Hypotheses

1. The values of fractional anisotrophy (FA) and mean diffucivity (MD) of the DTI will be significantly higher in the MTH+Xe group (see details in sections 5.7.1 and 5.12).
2. The imaging results with 11C-PK-11195 PET and ^{1}H-MRSI will differ in the groups of patients treated with xenon and hypothermia *vs* the group of patients treated with hypothermia alone. Neuroprotective properties of xenon and favourable neurological outcome will be demonstrated by showing less microglia cell activation in the combined therapy (xenon + hypothermia group) than in the reference therapy (hypothermia) group.

Poor neurological outcome will be associated with greater reduction in N-acetylaspartate (NAA) levels demonstrated by ^{1}H-MRSI at 2 and 10 days post cardiac arrest.

3. The serum biochemical marker NSE will be lower in patients treated with MTH+Xe, reflecting less severe hypoxic-ischemic injury and more efficient neuroprotection.
4. Cardiac enzyme release (Tn-T and CK-Mb) is less in patients treated with the combined therapy indicating a possible cardioprotective effect. The myocardial function imaged with transthoracic echocardiography is better preserved in the treatment group.

3 STUDY DESIGN

3.1 Type and design of the study

This is a two-centre **(Amendment 5.3 see Summary)** phase II, randomized, controlled two armed parallel follow-up study.

3.2 Randomisation and blinding

Randomisation will be performed in random blocks of several sizes. The randomisation will be performed independently and separately at each centre. **(Amendment 5.4 see Summary)** A sealed envelope containing the treatment assignment will be provided by the department of biostatistics in Turku University.
This trial will have two patient groups. The 1st group will be randomised to receive only MHT treatment. The 2nd group will be randomised to have MHT and xenon exposure. This is an open study but the physicians responsible for assessing the neurologic outcome within the first six months after the OHCA, and the neuroradiologist responsible for analysing the magnetic resonance images (MRI) are unaware of the treatment assignment.

3.3 General study outline and study schedule

The study consists of the following periods (see table of events, next page):

1. Screening
    a. Before hospital arrival
    b. Hospital arrival
2. ICU period
    a. Treatment induction,
    b. Treatment maintenance
    c. After treatment period
3. After ICU period before discharging from hospital
4. Follow-up period
   Long-term outcome assessed 3-3.5 years after cardiac arrest **(Amendment 6.8 see Summary)**

Schedule of events	Screening		ICU period			After ICU	Follow-up period		
			Treatment period		Intensive care period after treatment	Hospital stay before discharge	3 months after OHCA	6 months after OHCA	3-3.5 years after OHCA
Protocol activities	Before hospital arrival	Hospital arrival and ED	Treatment induction	Treatment maintenance					
OHCA	X								
Arrival to hospital =T 0h		X							
Brain CT		X							
Possible coronary intervention		X							
Decision to start MHT		X							
Inclusion and exclusion		X	X						
Informed consent		X	X						
Demography	X	X	X						
Medical history	X	X	X						
ED lab routines		X							
Arterial cannulation	X or	X or	X						
Central vein cannulation			X						
TCT measurement		X	X	X	X				
Blood gas analysis incl. Lactate and B-Gluc		X	X (every hour)	X (every hour)	X (every 2-4 h)				
Randomisation		X [1]	X [1]						
Active cooling starts for MHT			X						
MHT start time = TCT 33-34 °C achieved			X						
MHT period = TCT maintained				X (maintain 24 h)					
Xenon treatment (Xe 40 % in air/O_2)			X (must start within 4 h after T0h)	X (Maintain until stop MHT)					
Stop MHT and start rewarming (0.5°C/h to 36.5 °C)					X (MHT stop after 24h treatment)				
Maintain 36.5-37.0 °C until extubation or tracheostomy					X				
GCS in every 24 h			X (At T0)	X (at T24h)	X (at T48h ,T 72h, T96h)				
Neurological evaluation (CPC and mRS)							X	X	
1. MRI					X [2]				
2. MRI					X [3] or	X [3]			

3. MRI									X
PET					X [4]				
24 hour urine collection for determination of diurnal catecholamine*			X	X					
Tn-T, Ck-Mb, NSE, D-Lactate*, plasma catecholamine*, ECG		X or	X	X (at T24 h)	X (at T48,72h)				
TTE (all feasible study treatment patients)			X (prior MHT and Xe+during MHT prior Xe if feasible)	X (20 ± 4h after MHT start)	X (24 ± 4h after completed rewarming)	X (at discharge or 7d after Xe+MHT)			
TTE (all feasible control patients)			X (prior MHT)						
Continuous ECG, invasive BP, pulse oximetry			X [5]	X [5]	X (monitored only)				
ET Xenon concentration			X [6]	X [6]					
ET Oxygen concentration			X [6]	X [6]					
Electronic data collection and capture			X [7]	X [7]	X [7]				
Adverse events			X	X	X	X	X	X	
Concomitant treatment	X	X	X	X	X	X			
Long-term outcome									X
Recording on study specific CRF		X	X	X	X	X	X	X	X

X [1] Control (MHT) or study treatment (MHT+xenon)
X [2] performed within 16 hours after completion of rewarming the patient; see section 6.1.2. for details of MRI
X [3] performed 10 days after OHCA; similar MRI as the 1st MRI
X [4] performed within 24 hours after completion of rewarming the patient; only part of the patients will go through PET imaging
X [5] recorded and stored continuously
X [6] recorded and stored continuously
X [7] See details in section 10.2.
* Blood and urine samples will be collected, stored in -70 C and determined later by CRST
Abbreviations: BP = blood pressure; CPC = cerebral-performance category; h = hour; CT = computerized tomography; d = day; ED = emergency department; ET = end tidal; ICU = intensive care unit; GCS = Glasgow coma scale; h = hour; MHT = Moderate hypothermia treatment, mRS = modified ranking scale; MRI = Magnetic resonance imaging; NSE = neuron specific enolase; OHCA = out-of-hospital cardiac arrest; PET = Positron emission tomography; TCT = target core temperature; TTE = transthoracic echocardiography; Xe = Xenon;

An OHCA patient will receive the MHT as a standard care in the ICU of Turku University Hospital if cardiac resuscitation has been successful and the patient meets routinely used inclusion criteria. The inclusion criteria of this trial and the criteria in routine use are the same with one exception: patients with an asystole as a primary rhythm are excluded from this study. A patient will be recruited to this trial after the decision for MHT has already been made by an intensivist or other physician on duty. There will be a nominated group of investigators, i.e. recruiters, who will have a permission to recruit patients. The recruiter will be called upon by the physician after the decision for MHT has been made. A written informed consent will be obtained from the next of kin or the patient's legal representative. The patient can not be included if the written consent has not been obtained within 4 hours after arrival to the hospital. Patients recruited are randomized according to separately presented scheme to receive either standard hypothermia treatment (control group) (details in section 5.2) or hypothermia and additional xenon (details in section 5.3).

On arrival at an emergency department, the patients will undergo routine initial assessments and treatment including mechanical ventilation and correction of cardiovascular instability (details in section 5.4.). A brain CT-scan is performed for all patients before initiation of any treatments to exclude a possible cerebral origin of the cardiac arrest. The co-morbidity data including cardiovascular diseases and risk factors in addition to other chronic diseases are collected. Trombolytic therapy and possible invasive cardiac procedures must be performed before initiation of active cooling or xenon treatment. The pre-arrest co-morbidity and functional status, cause of death, patient's quality of life, in-hospital system factors, investigations and treatment, and physiological data at various time points during the first 72 hours are recorded on CRF (Table 1).

All patients require intubation and mechanical ventilation, and insertion of an arterial catheter, and insertion of a central venous catheter via jugular vein, subclavian vein or femoral vein. Central venous pressure can be measured also via endovascular cooling catheter if a separate central venous catheter can not be inserted.

The active cooling of the patient is initiated at arrival to the ICU. The patient will be cooled into a state of mild hypothermia with target core temperature of 33-34 ºC, which will be maintained for 24 hours. Possible xenon treatment will be initiated in the ICU immediately after the written informed consent has been obtained. The xenon treatment is continued until the MHT is completed (i.e. core temperature of 34 ºC is achieved). Otherwise, patient care will follow usual ICU protocols, which follow national and international recommendation. Determination and certification of brain death and withdrawal of active life support can be made according to legally and ethically accepted methods.

The neuroprotective efficacy of hypothermia in combination with xenon will be analysed with following brain imaging methods: Proton magnetic resonance spectroscopy will be performed for all patients; the 1st within 16 hours after completion of rewarming, the 2nd on day 10±2 and the 3rd 3-3.5 years after cardiac arrest (see details in 6.1.2.). Whenever feasible, positron emission tomography imaging will also be performed 5±2 days after OHCA (see details in 6.1.3.).

Evaluation of cardiac function is based on cardiac transthoracic echocardiography, analysis of cardiac enzyme release during three days after study or control treatments.

Neurological outcome is evaluated by a specialist in neurology 3 and 6 months after OHCA.

A long-term outcome will be evaluated 3-3.5 years after the OHCA by the investigators.

The primary and secondary endpoints are listed in section 6.4. The study patient may participate in another study after the follow-up of six months has been completed. This study will be performed during 2009-2013.

4 SUBJECT SELECTION

4.1 Source population

A male or female OHCA patient admitted to Turku University Hospital can be considered for enrolment to this trial only if the decision for MHT has already been made by an intensivist or other physician on duty. Thereafter, the patient meeting the inclusion criteria will be considered eligible for the study. Screening activities may be temporarily suspended in the event of inadequate medical staff to manage the study subject or excessive workload of study staff. During the temporary suspension, all eligible patients will be recorded in the subject screening log with a reason for exclusion, e.g. a temporary suspension.

4.2 Number of patients

Fifty-five patients will be recruited to each group, i.e. the MHT and MHT+Xe groups. See power analysis in section 8.2.

4.3 Inclusion criteria

The current criteria in use as a standard practise in Turku university hospital will be the same as the inclusion and exclusion criteria of this study with one exception: a patient with an asystole as a primary rhythm are excluded from this study.

1. Ventricular fibrillation or non-perfusive ventricular tachycardia as initial cardiac rhythm.
2. The 1st attempt at resuscitation by emergency medical personnel must appear within 15 minutes after the collapse.
3. The cause for collapse should be considered primary as cardiogenic and the return of spontaneous circulation (ROSC) should have been gained in 45 minutes after the collapse
4. Patient should be still unconscious in the emergency room.
5. Age: 18 – 80 years **(Amendment 2.9 see Summary)**
6. Obtained consent and possible xenon treatment initiated within 4 hours after arrival to the hospital

4.4 Exclusion criteria

(Amendments 2.1 and 2.2 see Summary)

1. Hypothermia (< 30°C core temperature)
2. Unconsciousness before cardiac arrest (cerebral trauma, spontaneous cerebral haemorrhages, intoxications etc.)
3. Response to verbal commands after the return of spontaneous circulation and before randomization
4. Pregnancy
5. Coagulopathy
6. Terminal phase of a chronic disease

7. Systolic arterial pressure < 80 mmHg or mean arterial pressure < 60 mmHg for over 30 min period after ROSC
8. Evidence of hypoxemia (arterial oxygen saturation < 85%) for > 15 minutes after ROSC and before randomization.
9. Factors making participation in follow-up unlikely
10. Enrolment in another study

4.5 Subject withdrawal/replacement

Study subjects are free to permanently discontinue their participation in the scheduled study assessments at any time without providing a reason or the study subjects can be permanently discontinued by their legal representatives as well. The study subjects can also permanently discontinue their participation in the study if it is recommended for any medical reasons by the investigator.

The study subjects may also discontinue their study treatment at any time, or it can be done by their legal representative. In this case, the study subjects will not be removed from the study and all scheduled study assessments will be completed. The data which have been collected before withdrawal will be used for study purposes in all cases of withdrawals or permanent discontinuations. After withdrawal the patient will be treated according to normal clinical practise.

All reasons of withdrawal will be recorded on the CRF. The withdrawn patients will not be replaced.

It must be noticed that the MHT will continue as a routine treatment for OHCA patients and it can be terminated only by attending physician on medical reasons. In the case of premature termination of MHT, the xenon treatment will be also terminated. In such case, a deviation from the protocol will be recorded on the CRF.

The study monitor will be notified about all discontinuations and withdrawals by email, phone or fax within 24 hours.

5 STUDY TREATMENTS

5.1 Control and study treatment

The control treatment is MHT. The target core temperature is 33-34 °C. MHT is defined as the time of maintaining target core temperature (TCT), which in this study is 24 hours. MHT of this study is conducted in accordance with national and international recommendations. The current routine MHT protocol of the ICU of Turku University hospital follows the recommendations. Hypothermia is induced with cold intravenous fluids and with endovascular cooling device. Usually, the core temperature has been achieved within 3 hours after arrival to the ICU. Also, most patients are already hypothermic at arrival to the hospital. However, in rare cases, the core temperature has been achieved 8 hours after arrival to the ICU. The target core temperature will be maintained for 24 hours. Thereafter, a maximum rate of 0.5 °C / hour rewarming **(Amendment 5.5 see Summary)**of the patients is allowed to a temperature of 36.5-37.0 °C, which will be maintained until the time of extubation or successful weaning from the respirator. The endovascular catheter is inserted via femoral vein by an experienced anaesthesiologist or intensivist. In endovascular cooling system (e.g. CoolGard 3000^{TM}, Alsius Co), temperature controlled saline circulates within a balloons in a closed loop; the saline never comes in contact with the patient. The cooling of the blood takes place

by contact with the balloon membrane (Microtherm™ , Alsius Co). The CoolCard 3000 can cool at a rate between 0.5 – 1.5 °C per hour depending on the endovascular catheter used (Icy™ or Cool Line™ catheter). Core temperature is measured with probes placed in oesophagus and in urinary bladder. After the actual rewarming procedure has been completed, the endovascular catheter will be hold in place until stabilization of the temperature can be assured.

The study treatment is inhaled xenon (LENOXe™) with subanaesthetic target concentration of 40% in oxygen/air combined with MHT. Details of the MHT are discussed above. The supplier is Air Liquide Deutschland GmbH (Germany). The Finnish National Agency for Medicines (Lääkelaitos) will be provided with the necessary details (including the supplier) of the investigational product before study execution.

5.2 Administration of study treatment

A patient can be recruited to this trial if the inclusion criteria, but none of the exclusion criteria, for standard MHT have been fulfilled. A patient will be recruited to this trial after the decision for MHT has already been made by an intensivist or other attending physician. Xenon treatment is initiated in the ICU as soon as possible after the written informed consent has been obtained. Xenon treatment may start before or after target core temperature has been achieved, but it must start within 4 hours after arrival to hospital, or otherwise the patient will be excluded. The xenon treatment will be continued until completion of MHT. The expected duration of xenon inhalation will be approximately 24 hours. Thus, in extreme cases, the duration of xenon inhalation can vary between 18-34 hours depending on the time needed to achieve the core temperature, and on the other hand, to obtain the consent. Xenon will be administered through closed-system ventilator, which is designed for delivery of xenon (PhysioFlex, Physio Dräger, Harlem, The Netherlands).

Target concentration for end-tidal xenon is 40 %. The concentration is adjusted by flushing the ventilation circuit with extra xenon flushes or nitrogen/air flushes/oxygen. The administration of xenon and the MHT are terminated at the same time (i.e. core temperature ≥ 34 °C). The xenon treatment will be terminated if the core temperature has to be elevated above 34 °C e.g. to treat bradycardia.

A failure to maintain 40 % concentration of xenon in oxygen during the treatment is not a reason to remove the study patient from the study. In such case, the treatment will be continued with a possible concentration which can be administered. The end tidal xenon concentration of 65 % may not be exceeded nor can it be less than 20% (i.e. the safety limits of xenon concentration).

The end-tidal xenon concentration is electronically monitored, recorded and stored continuously, and it is also recorded on specifically designed Case Report Forms (CRF) in every 30 minutes during the xenon administration.

5.3 Handling of study products

The Hospital Pharmacy of Turku University Hospital will import xenon. The investigator responsible for the study shall attend to that safety precautions and given instructions are carefully followed.

5.4 Prior and concomitant treatments

The patients will be treated according to a normal clinical practise prior and after the study treatment. All concomitant treatments must be recorded on the CRF.

5.4.1 Sedation and analgesia

Propofol-infusion (1-5 mg/kg/h) is used as sedative regimen and fentanyl-infusion (50-100 μg/h) and 25- 50 μg i.v. boluses will be administered as an analgesic regimen for all patients while intubated and mechanically ventilated. The depth of sedation will be adjusted according to the RASS scale with a target level of -5. The dosage of propofol and fentanyl can be increased if needed. Also, remifentanil or oxycodone can be used for analgesia as rescue treatment. Midazolam (bolus of 1-3 mg i.v.) is a first-line rescue treatment for sedative purposes. To prevent shivering and to maintain hypothermia, muscle-relaxant (e.g. cisatracurium) is given as bolus doses and/or by continuous infusion to all patients.

5.4.2 Mechanical ventilation

Adjustments of the ventilation parameters i.e. the ventilation mode, tidal volume and frequency are made according to normal treatment of patients in mechanical ventilation. The inspiratory oxygen concentration is adjusted to maintain the partial pressure for arterial oxygen in range of 10-18 kPa. The minute ventilation is adjusted to maintain the partial pressure for arterial carbon dioxide in range of 4.5 – 5.5 kPa. Repeated blood-gas analysis is performed at every hour during hypothermia and xenon treatment, and later whenever clinically indicated but at least in every 4 hours.

5.4.3 Hemodynamics and other

Systemic hypotension (mean arterial pressure < 60 mmHg) is primarily treated with crystalloid fluids or colloids.

Target mean arterial pressure	60-90 mmHg
Systolic arterial pressure	≥ 100 mmHg
Target central venous pressure (PEEP corrected)	6-10 mmHg

(PEEP= positive end expiratory pressure)

Vasopressors (e.g. epinephrine, norepinephrine, dopamine, dobutamine) and antiarrhytmic medication are administered as needed. Maintenance of fluid balance is monitored either by central venous catheter or by fluid intake and output.

Levosimendan (Simdax) can be used.

Hypertension is treated with vasodilators as needed.

Bradycardia treatment during MHT includes boluses of atropine (0.01 mg/kg) and/or a raise of body temperature (0.5 °C/h) to a degree that is considered necessary. Often in clinical practise, this means a completion of MHT. The final decision to increase body temperature during MHT can be made only by the attending physician. Xenon will be terminated if the core temperature is increased ≥ 34 °C. A decision to treat bradycardia is always based on clinical grounds, e.g. if general status of the

hemodynamics is considered to be inadequate. The final decision can be made by the attending physician only.

Serum glucose will be kept between 5.0 – 8.0 mmol/L (Amendment 5.6 see Summary) and hematocrit between 0.3 and 0.45. Potassium is supplemented if the serum potassium level is < 4 mmol/L. Parenteral nutrition or enteric feeding is initiated as soon as possible. An optimal head position of 30º is provided.

5.5 Treatment compliance

Any deviation from the defined protocol of the study treatment must be documented on the CRF. The end tidal concentrations of xenon will be monitored continuously and recorded electronically on a separate file during the treatment (source data). The concentrations will also be recorded manually on the study treatment CRF. Also, all air or xenon flushes used must be recorded. The absolute consumption of xenon by each patient will be measured afterwards by weighting the xenon cylinders.

5.6 Criteria for premature study termination

The independent safety committee and the PI will assemble for a meeting in every 6 months. The committee reserves the right to prematurely terminate the study prior to entry of the intended number of study subjects for valid scientific, ethical or administrative reasons. After such a decision, the investigator shall not enroll more subjects and he must inform the local ethical committee and the National Agency of Medicines of his decision.

The study of individual patient will be terminated by investigator or by the attending physician at any moment during the treatment in the ICU if safety of the patient can not be assured otherwise. Thereafter, the patient will be treated by the most appropriate means according to the judgment of the attending physician. Each individual case will be considered by the attending physician according to clinical evidence of intensive care medicine. It must be noted that various adverse events occurring during intensive care may not be related to the study treatment and will be considered individually by the attending physician.

The study subjects may be withdrawn from their study treatment prematurely by the investigator or by the physician for one or more of the following reasons:

1. A failure to maintain xenon concentration ≥ 20 %
2. A failure of ventilation and/or oxygenation of the patient with the xenon delivery device (Physioflex)
3. If MHT is terminated prematurely; a decision which can be done only by attending physician
4. AE/serious adverse event (SAE)
5. Protocol violation
6. If for any reason the investigator or the attending physician believes that continued participation in the study is not in the best interest of the patient.

A premature termination of the trial will be considered in the case clinically adverse outcome can be shown in the study treatment group after interim analysis (see 10.2). An interim analysis will be performed with 60 patients after they have undergone a 6-months follow-up.

6 ASSESSMENTS

All data obtained in Helsinki University Hospital will be transported to Turku University Hospital **(Amendment 5.7 see Summary)** (Address: Timo Laitio Department of Anaesthesiology, Intensive Care, Emergency Care and Pain Medicine, Turku University Hospital, Turku, Finland, P.O.X. 52, FIN-20521, Turku, Finland). Final analyses of all assessments will be carried out by investigators in Turku University Hospital.

6.1 Assessment of efficacy

6.1.1 Echocardiography

An experienced cardiologist will perform transthoracic echocardiography using Vivid 7 or Vivid Q devices (GE Healthcare) for all feasible patients as follows:

<u>Treatment group:</u>
Before initiating MHT or xenon
During MHT prior xenon administration if feasible
20 ± 4 hours after initiation of MHT
24 ± 4 hours after completing rewarming procedure
At discharge from hospital or 7 days after treatment

<u>Control group:</u>
Before initiating MHT
20 ± 4 hours after initiation of MHT
24 ± 4 hours after completing rewarming procedure
At discharge from hospital or 7 days after treatment

Measurements will be marked on data collection sheets (EXCEL file) and all image data will be stored in CDs/DVDs allowing off-line analysis with Echopack software.

Parameters:
Global left ventricular (LV) ejection fraction (LVEF) is calculated from the end-diastolic and end-systolic volumes (modified Simpson's rule) obtained from the apical four-chamber and two-chamber views according to the recommendations of European Association of Echocardiography.

Regional LV function/contractility will be evaluated using a standard 16 segment model. Each segment will be individually scored based on its motion and systolic thickening by visual analysis according to the recommendations of European Association of Echocardiography.

For quantitative analysis 2D longitudinal (and radial) Strain and Strain rates will be measured using speckle tracking imaging (automatic functional imaging, AFI) in apical four-chamber, two-chamber and long-axis views.

Parameters of diastolic function and left ventricular filling pressure (LVFP) will be measured from the transmitral and transpulmonary flow velocities and mitral annulus motion. Measurements from the transmitral flow will be early diastolic wave velocity (E), late diastolic wave velocity (A), late diastolic wave duration (Adur), deceleration time of early diastolic wave (Edec), propagation velocity of early diastolic flow (Epropag) and isovolumic relaxation time (IVRT). Derived measurement will be the ratio between early diastolic and late diastolic wave velocity (E/A). Parameters measured from the pulmonary venous flow will be systolic forward flow wave velocity

(S) and diastolic forward flow wave velocity (D). Derived measurement will be the ratio between systolic forward flow wave velocity and diastolic forward flow wave velocity (S/D). Tissue Doppler measurements from the septal mitral annulus will be velocity of early mitral annulus motion (E´) and late mitral annulus motion (A´). Derived measurement was the ratio between velocity of early mitral annulus motion and late mitral annulus motion (E´/A´) Derived measurement for the assessment of LVFP will be the ratio between early diastolic wave velocity and early diastolic velocity of mitral annulus (E/E'). According to these parameters, the patients will be assigned to four groups for having either normal diastolic function or mild, moderate or severe diastolic dysfunction. Patients whose parameters are borderline and suggestive but not definitive for diastolic dysfunction will be classified as indeterminate.

6.1.2 Magnetic resonance imaging of the brain

Diffusion Tensor Imaging (DTI) and diffusion-weighted MRI identify early ischemia-related changes of the brain and offers great potential for prognostic value after cardiac arrest or stroke. Brain gray matter is more vulnerable to ischemia and hypoxia than white matter. The primary sites that are acutely affected in hypoxia or ischemia are the basal ganglia, thalamus, cerebral cortex (in particular the sensorimotor and visual cortices), cerebellum and hippocampus. The grey matter is preferentially affected in acute phase because it is metabolically more active than white matter as it contains a large number of synapses, which are vulnerable to ischemia. In the early phase after cardiac arrest brain damage is characterized by brain swelling and cortical necrosis. Late manifestations are white matter degeneration, volumetric changes, atrophy of the both white and gray matter and dilatation of the cortical cerebro-spinal fluid spaces and ventricles. Also an amount of hemosiderin found in the later phase reflects the severity of the brain damage. Hemosiderin is a degradation product of haemorrhages in the brain tissue and can be specifically imaged by a susceptibility-weighted imaging (SWI) sequence. SWI can also be used to estimate local neurodegeneration of basal ganglia and various brain cortical areas

DTI is a proton magnetic resonance method, which can find subtle changes in brain white matter structure. Mean Diffucivity (MD) in DTI method that reflects the free water movement in a tissue, whereas Fractional Anisotrophy (FA) reflects the directionality of diffusion and can be used in estimating white matter integrity (42). Restricted diffusion and low MD is typical finding in acute ischemic brain insult inside the lesion and reflects the depth and extent of the ischemia. Later on, pseudo-normalization of MD in the infarcted tissue occurs, followed by high free water diffusion values reflecting severe brain tissue damage. In late brain ischemia, white matter tract degeneration of the corresponding tracts of the infarcted area can be evaluated with lowering of the FA values and visually on 3D tractography based on DTI (43).

Brain perfusion imaging with contrast-enhanced MR perfusion can be used in evaluation of the extent of hypoperfusion of the brain tissue. The hypoperfusion of the tissue can be evaluated by blood volume and flow maps and also the tissue at risk, the so called ischemic penumbra can be evaluated on the miss-match maps based on blood volume and flow maps. The volume of both infarcted tissue and tissue at risk can be measured based on perfusion MR data.

Proton magnetic resonance spectroscopy (1H-MRSI) has been used to explore cerebral metabolic dysfunction, and permits sensitive, non-invasive assessment of neurochemical alterations in selected brain regions after cerebral injury and ischemia. This technique may be used to identify “injured” tissue that is not readily picked up by conventional imaging techniques and this information can aid in determining clinical neurologic prognosis (44). One of the metabolites measured by 1H-MRSI is NAA. NAA is ubiquitous in the central nervous system, and is a marker of mature neurons;

persistent reductions in NAA have been widely used as a marker of neuronal loss (45,46) Conversely, transient reductions in NAA are thought to be the hallmark of mitochondrial dysfunction (47), as NAA is synthesized in neuronal mitochondria (48), and production of NAA and mitochondrial metabolism are inextricably linked (49,50). Several studies have demonstrated that high levels of NAA are associated with better outcomes (51,52). Furthermore, the demonstration of substantial correlations between cerebral metabolite alterations and functional outcome in a controlled experimental setting provides further evidence for the validity of clinical studies suggesting that 1H-MR spectroscopy may play an increasing role in predicting outcome in patients with ischemic brain injury; neurological outcome prediction following stroke and OHCA is a clinical imperative. 1H-MRSI can also be used to measure several other brain metabolites, including glutamate/glutamine, choline, total creatine and lactate. Increased lactate also suggests deranged energy metabolism and is consistent with cerebral ischemia (53). A new method of Tract Based Spatial Statistics (TBSS) with diffusion tensor magnetic resonance imaging will be applied. **(Amendment 2.5 see Summary)**TBSS is an automated observer independent method of aligning fractional anisotrophy (FA) images from multiple subjects to make non-biased assessments of localized ischemic changes in the major white matter tracts. TBSS allows statistically powerful assessments of white matter microstructure and the separation of small groups of subjects, thus reducing the sample size significantly compared to using original visual analysis. All diffusion data acquisition requirements are already fulfilled and therefore MRI protocol will not be changed.

Brain temperature is measured noninvasively using proton MR spectroscopy**.(Amendment 2.4 see Summary)** The temperature is determined by the balance between heat produced by cerebral energy turnover and heat removed by cerebral blood flow. The purpose of this analysis is to investigate the possible mechanism of the neuroprotective effect of the combination of xenon and therapeutic hypothermia. A hypothesis is that xenon improves the neuroprotective effect of hypothermia by further decreasing the temperature of the brain tissue. The magnetic resonance imaging time will be increased by 2 minutes, i.e. from 50 minutes to 52 minutes.

All patients will go through the MRI (3.0 TE Siemens Verio), one within 16 hours after completion of rewarming the patient, i.e. approximately 36-52 hours after cardiac arrest, and the second MRI will be performed 10 ± 2 days **(Amendment 2.3 see Summary)** after OHCA. A third MRI including a susceptibility-weighted imaging (SWI) sequence will be performed in all survived patients 3 to 3.5 years after cardiac arrest in order to investigate the presence and pattern of long-term progression of brain damage and degeneration.

The patients will remain intubated and mechanically ventilated in a respirator until the first MRI scans have been performed. An experienced anaesthesiologist or intensivist will take care of the patient during the imaging. MRI scans will include proton density/T2 weighted (PDT2) multiecho images; fluid attenuation inversion recovery (FLAIR) images. T1-weighted FLASH images will be acquired for volumetric analysis. In addition, 1H-MRSI will be used to measure NAA, choline, and lactate and other brain metabolites will be performed in a multivoxel manner covering basal ganglia, thalamus and frontal white matter. The 1H-MRSI data from each time point will have a fixed multi-voxel grid overlaid allowing direct comparison between spectra collected. DT images will be used for measurement of the FA and MD of the normal and infarcted tissue. FA of the clinically corresponding and relevant white matter tracts will be measured to evaluate possible remote Wallerian degeneration.

The MR images will be analyzed at the Department of radiology by the Physicist Jani Saunavaara, neuroradiologist Riitta Parkkola and Sami Virtanen. They are blinded to the patient treatment group.

The MRI data will be analysed as follows: Routine MRI will be evaluated on standard PACS (Kodak Carestream) and perfusion and diffusion tensor imaging post-processing will be performed on Siemens Verio post-processing work station (Leonardo, Siemens Medical Imaging, Erlangen, Germany). The proton MR spectra data will be analysed using LCModel (S Provencer, Canada), which contains normal brain spectra data of the brain of 10 000 healthy volunteers. The results of metabolite rates received in this study are compared to the normal data of the LCModel. On 2D images ROI-measurements of the MD, FA, blood flow, blood volume, TTP, ischemic penumbra and metabolites will be performed. 3D tractography of DTI data will be performed to visualize and assess the white matter tracts in volume images. An advanced post-processing method is used in the volumetric analysis and applied in all three MRIs per patient. **(Amendment 6.7 see Summary)** The post-processing analysis will be performed with SPM8 software (Wellcome Department of Cognitive Neurology, London, UK, http://www.fil.ion.ucl.ac.uk/spm/). A susceptibility-weighted imaging (SWI) sequence will be carried out in the 3rd MRI protocol. SWI increases the imaging time by 5 minutes from 52 minutes to 57 minutes.

Constrained spherical deconvolution (CSD) **(Amendment 7 see Summary)**
Diffusion-weighted (DW) magnetic resonance imaging (MRI) is a noninvasive imaging method to investigate white matter microstructure and neural fibers. Traditionally the most commonly used method for the analysis of DW data is diffusion tensor imaging (DTI) (54). However, the shortcoming of DTI is that crossing fibers, present in 60-90 % of white matter voxels, cannot be correctly identified (55).
Constrained spherical deconvolution (CSD) is a new technique that estimates the orientation of multiple fiber populations, e.g. crossing fibers, in a voxel accurately and within a clinically feasible scan time (56,57). We will estimate the fiber orientation distributions with CSD and use this information to perform probabilistic fiber tractography for the whole brain and for certain tracts of interest (58).

Network analysis
The whole brain tract networks will be analyzed with graph theoretical analysis (59), which has been previously applied successfully in for example schizophrenia and traumatic brain injury [60, 61). The tract networks are complex, and graph theoretical analysis enables the efficient investigation of both local and global properties of the whole brain network.
DW data will be acquired with a 3T spin-echo echo-planar imaging (EPI) sequence with TR=9.1 s, TE=121 ms and 2.5 x 2.5 x 2.5 mm^3 voxel size. Field of view is 240 x 240 cm^2 with a 96 x 96 acquisition matrix and the number of excitations is 1. 56 axial slices are imaged with 2.5 mm thickness and no gap. Diffusion gradients with b = 2500 s/mm^2 will be applied in 60 directions uniformly distributed on a unit sphere (62). Nine images with b = 0 s/mm^2 will be acquired. In addition, eight b = 0 s/mm^2 images with reverse phase-encoding will be acquired to correct for EPI distortions (63).

6.1.3 PET imaging of the brain (not to be performed in Helsinki)

(Amendment 4.2, 5.2 and 5.8 see Summary) The PET scans can be performed only during weekdays which will decrease the number of scans to be carried out at the designated time. It will be carefully evaluated which of the clinical study subjects are in such a condition that they can be considered eligible for the PET study. Each clinical study subject will undergo brain MRI within 16 hours after completion of the rewarming procedure (i.e. 36-54 hours after cardiac arrest). A patient with multiple areas of cytotoxic cerebral oedema indicating severe hypoxic-ischemic cerebral injury

revealed by MRI will be considered to have a poor prognosis and therefore will not undergo the PET scan. Only patients with no more than minor cerebral ischemic injuries, stable hemodynamics, and normal ventilation and oxygenation will be considered eligible for PET scans. The PET scan will be carried out 5±2 days after cardiac arrest. **(Amendment 4.1 see Summary)**Given the non-elective nature of the patients, schedule of each PET scan will be adjusted individually regarding logistical, feasibility and safety aspects. Patients will be sedated primarily with dexmedetomidine if needed. A secondary choice of sedation will be propofol which can also be combined with dexmedetomidine, especially if patient is still intubated and mechanically ventilated. Muscle relaxation will be maintained with subsequent incremental boluses of rocuronium if necessary. At least one of the study physicians and nurse of the ICU team will be present throughout the PET study.

It will be explored whether 11C-PK 11195 PET can be used to test the hypothesis of neuroprotective effect of xenon. This could be demonstrated by showing less microglial activation in the combined therapy (xenon + hypothermia group) than in the reference therapy (hypothermia) group. PK11195 binds specifically to the "peripheral benzodiazepine binding site" (PBBS), also called 18-kDa Translocator Protein (TSPO). The PBBS is expressed by mitochondria in cells of the mononuclear phagocyte lineage and within the central nervous system is highly expressed by activated, though not resting, microglia - the brain's intrinsic population of tissue macrophages. PK 11195 has been labelled with carbon-11, which is a positron emitting isotope. Thus, 11C-PK11195 can be used in positron emission tomography (PET) to visualize microglial activation. In combination with volumetric MRI to provide detailed structural information, the enantiomeric PET ligand [^{11}C](R)-PK11195 has been used to measure microglial activation in acute and chronic inflammatory and non-inflammatory, brain diseases.

Microglial cell activation has been implicated in the pathogenesis of several neurodegenerative disorders and to be involved in other conditions where inflammation is involved. Inflammation and microglial activation is considered as one possible mechanism of destruction. Thus, measurement of microglial activation is expected to be a valuable tool for use in monitoring the effect of hypothermia and combined xenon and hypothermia therapy after cardiac arrest.

In order to ensure the feasibility of the PET procedure, a pilot study with 4 patients from either of the treatment groups will first be carried out. In the case of successful pilot study, further 16 subjects will be investigated with 11C-PK11195 and the effect of hypothermia or combined xenon + hypothermia therapy will be compared in a parallel fashion. Thus 8 subjects will be scanned with 11C-PK 11195 5 after hypothermia therapy and 8 subjects after xenon + hypothermia therapy.

PET scanning will be performed with GE Discovery VCT PET-CT device. This gives the possibility to obtain in addition to 11C-PK11195 uptake also anatomical information with CT. The PET-tracer to be used is [11C]-PK11195. The subjects need to have both venous (for tracer injection) and arterial catheters (for radioactivity concentration and metabolite measurements). The scanning will be performed in a dimly lit room. After transmission scanning (performed for attenuation correction), approximately 200-550 MBq of [11C]-PK11195 will be given by i.v. injection. A dynamic 60 min study will be performed with a protocol that yields 17 time frames in 3D mode: 2x15s, 3x30s, 3x60s, 7x300s and 2x600s. During the acquisition, continuous arterial sampling for the first 5 minutes with a flow of 6 ml/minute and sampling frequency 1 sample/second will be carried out. A blood sampling for metabolites and/or total blood activity will follow the acquisition period. A total amount of blood samples will not exceed 100 ml per patient.
The scanning data will be corrected for attenuation and scatter and radioactivity decay. Traditional region of interest (ROI) based method will be used to quantify 11C-PK 11195 uptake using metabolite purified arterial blood activity as an input function. The possibility to use automated ROI analysis (autoROI) and other ways to quantify 11C-PK11195 will be also explored.

The study subjects will be exposed to radiation, but the radioactivity from PET tracers is short lived. The dose of radiation for one subject from 11CPK 111965 scan will be about 2,1 mSv which equals to less than one year dose of background radiation (around 4-5mSv).

6.1.4 Neurological evaluation

Neurological outcome will be defined according to Pittsburgh cerebral-performance category (CPC category) and Modified Ranking Scale (mRS). The physician responsible for assessing the neurologic outcome within the first six months after the arrest is unaware of the treatment assignment. Pittsburgh cerebral-performance category is defined as follows: CPC 1, conscious and alert with normal function or only slight disability, good recovery; CPC 2, conscious and alert with moderate disability; CPC 3, conscious with severe disability; CPC 4, comatose or in a persistent vegetative state; CPC 5, certified brain death or dead by traditional criteria. Best neurological response is assessed at 1 h, 24 h, 48, 72 h, and 96 h after the collapse using Glascow Coma Scale (GCS) scale. Additionally pupillary response and involuntary movements, such as seizures, myoclonus and extension reactions are reported. Recovery from coma is defined by following verbal commands. A best value of CPC and mRS will be defined during hospital stay (i.e. a single item) and at six months.

6.1.5 Long-term outcome

The long-term outcome will be assessed at 3-3.5 years after the cardiac arrest by telephone, or by seeing the patient at home or at study physician's appointment. Also, deaths and reasons for deaths of all randomised patients will be verified from the national Cause-of-death Register at six months and at 5 years after cardiac arrest.

6.1.6 Biochemical assessments

A blood samples for determination of plasma troponin-T (P-TnT), plasma crietinine-cinase MB-subunit, mass (P-CK-MBm), and serum neuron-specific enolase (S-NSE) are obtained at arrival to ICU, and 24 hours, 48 hours and 72 hours after OHCA.. **(Amendment 2.8 see Summary)**

P-TnT, P-NSE and P-CK-MBm will be determined by TYKSLAB in Turku University Hospital and by *HUSLAB in Helsinki University Hospital* **(Amendment 5.10 see Summary)** with standard methods. The blood will be collected into three 5 ml tubes for determination of TnT, CK-MBm and NSE.

A blood sample of 15 ml will be collected at arrival to ICU, at 24 hours, at 48 hours and at 72 hours for later determination of catecholamines and D-lactate, and plasma will be promptly separated in a refrigerated centrifuge, aliquoted into 4 storage tubes, frozen at -20C and transferred within 24 h to -70 °C for storage. Catecholamine concentration will be determined with HPLC and coulometric electrochemical detection by *Clinical Research Services Turku CRST* CRST using established methods with intra- and inter-assay CVs below 10% in the relevant concentration range. D-lactate will be determined by CRST as well.

All urine produced and excreted during the MHT will be collected, aliquoted and stored for later determination of catecholamines.

An additional blood sample of 10 ml is drawn and stored in -70 °C for possible later use. The blood and urine samples obtained and stored in -70 ºC in HUSLAB will be transported to Turku CRST for later determination of catecholamines **(Amendment 5.11 see Summary)**.

Quantitative interpretations of signal are confounded by large interindividual variability in binding affinity of 11C-PK11195 to the PBBS (also called TSPO) displaying a trimodal (i.e.low-, mixed- and high-affinity binders) distribution compatible with a codominant genetic trait. A complete agreement was recently observed between the TSPO Ala147Thr genotype and PBR28 binding affinity phenotype (P value = 3.1_10_13). The TSPO Ala147Thr polymorphism predicts PBR28 binding affinity in human platelets. As all second-generation TSPO PET radioligands tested hitherto display a trimodal distribution in binding affinity analogous to PBR28 (e.g. C11-PK 11195 used in our study), testing for this polymorphism may allow quantitative interpretation of TSPO PET studies with these radioligands.Therefore, a blood sample of 20 ml for determination of an individual TSPO Ala147Thr polymorphism will be obtained from each patient enrolled to the PET scans (not to be performed in Helsinki). The subjects will have indwelling arterial lines already as part of the procedures stipulated by the existing protocol, so new cannulations will not be required. An additional assent will be obtained from the next of kin or the legal representative for this assessment.

Reference: Owen DR et al. An 18-kDa Translocator Protein (TSPO) polymorphism explains differences in binding affinity of the PET radioligand PBR28. Journal of Cerebral Blood Flow & Metabolism advance online publication, 19 October 2011; doi:10.1038/jcbfm.2011.147

6.1.7 Transcranial Doppler (not to be performed in Helsinki)

(Amendment 2.6 and 5.8 see Summary) An experienced neurologist will perform a transcranial doppler (TCD) imaging to all feasible patients. A following TCD protocol will be applied whenever feasible:

Treatment group:
Before initiating MHT or xenon
During MHT prior xenon administration if feasible
20 ± 4 hours after initiation of MHT
24 ± 4 hours after completing rewarming procedure
At discharge from hospital or 7 days after treatment

Control group:
Before initiating MHT
20 ± 4 hours after initiation of MHT
24 ± 4 hours after completing rewarming procedure
At discharge from hospital or 7 days after treatment

6.1.8 Cerebral carbon dioxide reactivity test (not to be performed in Helsinki)

(Amendment 3.2 see Summary) The effect of xenon inhalation and hypothermia treatment on cerebral carbon dioxide reactivity will be tested on all eligible patients. Reactivity test will be repeated twice; during MHT/MHT+xenon treatments and during room air/oxygen inhalation (baseline). The effect of xenon on cerebral blood flow will be assessed using trans cranial Doppler sonography (see TCD protocol). First the flow velocity in three arteries bilaterally (anterior, middle and posterior cerebral arteries) is measured. Thereafter ventilation is increased to allow 1kPa decrease in arterial partial pressure for carbon dioxide. Thereafter the flow assessments are repeated.

6.1.9 Heart rate variability

Heart rate variability will also be assessed in all patients. GE Datex-Ohmeda S/5 Anesthesia Monitor and a portable computer running the S/5 Collect software will be used for recording continuous ECG (standard patient monitoring leads) with 1028 Hz sampling. The recording will be completed 24 hours after the normal body temperature has been achieved. The Holter ECG data is sampled digitally and transferred to a microcomputer. Careful manual editing of the RR-interval tachograms with inspection of the ECG data by deleting premature beats and noise is performed. All RR-intervals of suspected portions are printed-out on a 2-channel ECG at a paper speed of 25 mm/s to confirm the sinus origin of the RR-interval (i.e. beat-to-beat interval) data as previously described (64). The same edited data is used for all heart rate variability measures in this study. Mean length of RR-intervals and standard deviation of all RR intervals (SDNN) are used as time-domain measures of heart rate variability. RR-interval spectrum is computed according to standard procedures (65). In addition, various non-linear methods, such as Poincaré plot analysis, detrended fluctuation analysis and approximate entropy, will be applied as previously described (64). WinCPRS software programs will be used in the analyses. Spontaneous baroreflex sensitivity (BRS) can be evaluated by analysing the slopes of spontaneously occurring sequences of three or more consecutive beats in which systolic blood pressure (SAP) and pulse interval of the following beat change in the same direction (either increase or decrease), in a linear fashion. Individual BRS can be obtained by averaging all slopes computed within a given test period i.e. a linear regression is applied to each individual sequence and the mean slope of the SAP/RRI relationship is calculated and taken as a measure of the spontaneous BRS for that period. Spontaneous baroreflex analysis provides good BRS reproducibility under various stimuli that affect the neural control of circulation differently. Baroreflex effective index will be also measured. It quantifies the number of times the baroreflex is effective in driving the sinus node (66,67).

6.1.10 Esophagus pressure measurements

(Amendment 3.1 see Summary) Transpulmonic pressure measurements will be performed for all eligible patients both in the xenon treatment group and in the control group. Pressure assessment will be done using oesophageal balloon catheter (Marquat Genie Biomedical, Boissy-Saint-Léger Cédex, France). Catheter will be inserted in the oesophagus of the patients upon arrival in the intensive care unit. The probe is placed in the middle / inferior third of the oesophagus and it is filled with 0.5 – 3 ml air during the measurements. The orifice is attached to a low range differential pressure transducer (Extech Instruments, Maine, USA) in order to measure the pressures. Data will be stored online into a laptop using USB connection of the device and the software provided by the manufacturer (Extec Instruments). The probe will be removed after pressure assessments (see protocol below).

Esophageal pressure protocol

All measurements will be performed in 5 minute epochs in all feasible patients.

Additional measurements will be carried out at the time of significant adjustments to ventilator settings whenever otherwise feasible.

Each of the following 4 periods in each group will contain 2 epochs.

Control group (MHT)

1. During cooling before target temperature has been achieved
2. During MHT
3. During warming

4. After normal body temperature has been achieved

Treatment group (Xe+MHT)

1. Before start of xenon and during cooling before target temperature has been achieved
2. During Xe+MHT
3. During warming
4. After normal body temperature has been achieved

6.2 Assessment of safety

A brain CT-scan is performed for all patients before initiation of any treatments to exclude a possible cerebral origin of the cardiac arrest.

An independent safety committee conducted by Turku CRC will be responsible for both centres. **(Amendment 5.9 see Summary)** A monitor from both centres will be involved throughout the trial. Turku CRC will carry out monitoring in Turku University Hospital and the Clinical Research Institute Helsinki University Central Hospital Ltd. (HYKS-instituutti) will be responsible for monitoring patients enrolled in Helsinki University Hospital.

The subjects will be continuously monitored according to standard care of patients in ICU (i.e. continuous invasive blood pressure, continuous 2-channel ECG, pulse oximetry, central venous pressure, body temperature, blood gas assessment at least once per hour, continuous end-tidal CO_2 %, ventilatory frequency, tidal volume, minute volyme, inspiratory O_2 %).

The concentration of end-tidal xenon and oxygen is electronically monitored, recorded and stored continuously.

The following safety limits have to be maintained and adjusted accordingly. All deviations will be recorded on the CRF.

Mean arterial pressure	60 – 100 mmHg
Systolic arterial pressure	>100 mmHg
Heart rate during MHT	>30/min
Heart rate after MHT	>40/min
Xenon concentration	20 – 65 %
PaO_2	9 - 20 kPa
Central venous pressure	4 – 14 mm Hg (PEEP corrected)
Serum glucose	4.5 – 6.5 mmol/L
Hemoglobin	> 70
Potassium	3.0 – 5.0 mmol/L (temperature dependent)

See section 5.4.: treatment instructions.

Maintenance of fluid and electrolyte homeostasis is monitored either by central venous catheter or by fluid intake and output.

Parenteral nutrition or enteric feeding is initiated as soon as possible after first 24 hours of arrival to ICU. An optimal head position of 30º is provided.

In addition, during PET scanning the patients will be monitored according to standard care of patients in ICU. A study physician will always be present at the PET study site.

Routine laboratory tests: small blood count, C-reactive protein, platelet count on Tuesday, Thursday, Saturday, ad on Sunday and small blood count, C-reactive protein, creatinine, Urea, platelet count, bilirubine, on Monday, Wednesday, and on Friday

Patient care during treatment and imaging procedures

During office hours, there are altogether 4-6 anesthesiologists and intensivists as attending physicians in the ICU. Beyond office hours, there are two physicians, one anesthesiologist and one specialist in internal medicine, on duty. Several research (5-10) nurses will be trained to administer xenon. The research nurses will be involved in the xenon treatment most of the time but the study physician will confirm the safety of the treatment at all times. The study physician will always initiate the xenon treatment and assure that its administration can be managed safely in accordance with the protocol. The study physician and research nurse will always be available during the MHT treatments. **(Amendment 1 see Summary)** In addition, study physician can be reached by telephone during the period of ICU treatment. However, study physician can not be involved in any treatments including emergency situations during the MHT or MHT + Xe treatments.

The temperature management and cooling procedure have been standard care for the out-of-hospital cardiac arrest patients for at least five years in the ICU of Turku University Hospital. All study physicians of the ICU team and other attending physicians have been involved in treating patients with hypothermia. The mechanical ventilation will be continued until the first MRI and the possible PET imaging have been performed. An anesthesiologist or intensivist and a nurse will take care of the patient during the brain imaging procedures.

6.3 Other assessment

The concentration of end-tidal xenon and oxygen is electronically monitored, recorded and stored continuously. In addition, it is recorded on specifically designed Case Report Forms (CRF) in every 30 minutes during the xenon administration.

6.4 Primary and secondary variables

Primary outcome is to show a significant reduction in the degree of severity of the ischemic brain injury in the MHT+Xe group as compared with the MHT group, reflected by following MRI:

a) Brain tissue anatomy: T2, FLAIR and 3DT1 images
b) Depth and extent of the ischemia: Mean diffucivity value of diffusion tensor proton magnetic resonance imaging (DTI)
c) White matter tract degeneration of the corresponding tracts of the infarcted area: Fractional Anisotrophy value of DTI
d) Extent of hypoperfusion of the brain tissue: Perfusion MRI Data
e) Cerebral metabolic dysfunction: Proton magnetic resonance spectroscopy

<u>In detail:</u>

1) Routine T2, FLAIR and 3DT1 images give detailed information about the brain tissue anatomy. The cerebro-spinal fluid spaces (ventricles and cortical fluid spaces) can be evaluated and volume

measured on these images. 3DT1 images can be used as an anatomic reference images for PET-studies. Volume of possible old and new ischemic insults can be seen and measured by means of DTI.

2 and 3) On DTI data, the fresh ischemic insults can be detected and differentiated from old insults. The volume of fresh infarcts and ischemic areas is evaluated. The MD values of the ischemic area reflect the depth of ischemia. Normal range of MD of both the grey and white matter is from 0.75 to 0.85 10^{-3} mm^2 s^{-1}. In acute ischemia, the MD values of the ischemic brain tissue decreases to 0.4-0.5 x 10^{-3} mm^2 s^{-1} and then gradually increases to the pseudonormal phase value at about 2 weeks after insult and then increases to the value of cerebro-spinal fluid in months. The FA values of the neural tissue range from 0 to 1. Value near zero can be measured on areas, where no tract bundles can be expected. On strong and healthy tracts like pyramid tract values up to 0.8 to 0.9 can be expected. The FA values of clinically relevant tracts will be measured respective to the ischemic grey matter areas related to the cardiac arrest prior to this study.

4) Perfusion MR Data gives semi-quantitative brain blood volume maps, brain blood flow maps and Time-to-Peak maps of the whole brain tissue. On these maps the depth and volume of blood volume and blood flow loss of the ischemic area can be measured relative to blood volume and flow of the areas of normal perfusion of the brain. Based on blood volume and flow maps, ischemic penumbra `the tissue at risk` can be evaluated and volume measured.

5) The brain MR spectroscopy gives data about the metabolite ratios of the brain tissue. The metabolite rates will be measured at the basal ganglia and thalamus and frontal white matter. The spectra data will be analyzed using LCModel post-processing software (S Provencer, Canada). This software contains the normal brain spectra metabolite values of about 10000 healthy volunteers. The results of the recent work are compared to this normal database of the post-processing program.

Secondary end-points will be neurological outcome within six months, mortality (non-cardiac death, cardiac death, sudden cardiac death), morbidity (myocardial infarction, cardiac arrhythmias needing treatment or hospitalization, stroke) at six months, and the complication rate (according to HACA study: severe bleeding, pneumonia, sepsis, pancreatitis, renal failure, pulmonary oedema, seizures, arrhythmias, pressure sores) during the first week.

7 ADVERSE EVENTS

7.1 Definitions

An Adverse Event (AE) is any untoward medical occurrence in a study subject to which the study treatment may or may not have positive causal relationship. An unexpected adverse drug reaction is defined as an adverse drug reaction, which or the severity of which is not available in the information submitted to the investigator. It implies a positive judgment of causal relationship to the study drug by the investigator.

An AE may be reported to the investigator by the study subject or his/her caregiver or observed by the investigator clinically, or be an adverse change in laboratory assessment results. An AE may be a subjective symptom, worsening of existing illness, newly appearing disease, accident or a new finding in a clinical or laboratory assessment.

Study subjects participating in this study already need intensive care due to one or more organ insufficiency or failure. Therefore, multiple and diverse clinical symptoms and laboratory findings

must be expected to occur frequently. Expected minor fluctuations in the study patient's presenting illness would not represent an AE. Any clinically significant worsening in a study patient's condition according to clinical judgement, labarotory finding or other diagnostic finding, compared with the study patient's baseline status at the time of starting xenon treatment should be recorded as an AE whether the worsening condition is considered to be due to the study patient's underlying illness or study treatment.

A Serious adverse event (SAE) is any AE in a study subject of the treatment group that is
- fatal,
- life-threatening,
- requires or prolongs inpatient hospitalization,
- results in persistent or relevant disability or incapacity,
- is a congenital anomaly/birth defect or
- another medically significant event, e.g. an intervention to prevent one of the above outcomes.

In this study, a careful judgment is needed when reporting SAEs, since the patients are already in a life-threatening condition and their length of ICU stay or hospitalization cannot be predicted. Therefore, AEs cannot readily be judged as life-threatening condition or the reason for prolonged ICU stay or hospital stay. Furthermore, in this study MHT itself as a routine treatment may cause clinical perturbations or clinical worsening of the study patient's condition. In such case, if xenon treatment is initiated simultaneously with MHT, it may be difficult to distinguish which one of the treatment is the cause for any adverse affect. Any persistent disability would not necessarily indicate an SAE, if it was predictable from the study patient's clinical condition at the time of starting study treatment. However, any newly-emergent condition that meets the above definitions of an SAE in the study treatment group should be recorded as an SAE including death in all circumstances. In addition, the investigator should make best efforts to identify any clinically significant worsening of the study patient's underlying condition that meet the definition of SAE. However, all safety limit deviations, clinical symptoms and events or other major fluctuations in all patients will be recorded in the CRF and evaluated by the independent safety committee.

7.2 Reporting of adverse events (AEs)

All AE:s must be elicited, documented and reported by the investigator from the moment of treatment until the end of the follow-up period of six months. All AEs must be recorded by the investigator on the appropriate space in the CRFs irrespective of the causal relationship of the study treatment to the event as assessed by the investigator. The independent safety committee will check and evaluate the reports of AEs.
The investigator should provide the following information in all cases:
1. onset and resolution time points of the AE
2. frequency of occurrence
3. degree of severity
4. causal relationship of the study treatment to the AE
5. how the AE came to the attention of the investigator
6. subject outcome
7. absence of AEs should also be documented.

7.3 SAE reporting

Most of the SAEs are also end points in this study, and therefore a summary of SAEs and suspected unexpected serious adverse reactions (SUSARs) will be reported by the principal investigator to the

National Agency for Medicines (NAM) in every 6 months instead of employing the“7 and 15 day rule”. The principal investigator will report SAEs to safety committee in every month. The independent safety committee must report to NAM within 7 days after finalizing their reports.

7.4 Treatment of emergencies

The personnel of the ICU are experienced in handling all kinds of emergencies. Emergencies will be treated according to state of the art in ICU by attending physicians and investigators.

See section 5.6., criteria for premature study termination

8 STATISTICS

8.1 Statistical hypothesis

Our objective is to show a significant reduction in the degree of severity of the ischemic brain injury in the MTH+Xe group as compared with the MTH group, reflected by MRI.

8.2 Sample size

The power analysis is based on the FA values of the DTI data. FA reflects the directionality of diffusion and can be used in estimating white matter integrity. Restricted diffusion is typical finding in acute ischemic brain insult inside the lesion and reflects the depth and extent of the ischemia. The study is a randomized two-armed parallel follow-up study. Fifty-five patients per treatment group are needed to show an absolute median difference of 15 % in FA values between the groups (85 % power, $\alpha < 0.05$). The median difference of 15 % is considered as clinically relevant.

8.3 Statistical plan and analysis

The univariate distributions of variables and the associations between variables will be described using summary statistics. Basic statistical tests (t-tests, Mann-Whitney, Chisquare, etc) will be used in the preliminary analysis.

Survival analysis methods will be applied for the occurrence of end-point events. An analysis of variance of repeated measurements will be applied for normally distributed response variables. In the case of other type of variables, the analysis will be based on generalized linear mixed models which include comprehensive and general tools to analyse longitudinal data. Baseline characteristics of patients will be used as covariates when needed.

The statistical analysis will be based on all available data. If an analysis is based only on complete cases, it will be mentioned separately. The need for ITT analysis will be considered case by case.

Significance level of 0.05 and an estimation of 95 % confidence intervals will be used in the statistical analyses.

8.4 Interim analyses and stopping rules

An interim analysis will be performed with 60 patients after they have undergone a 6-months follow-up. A premature termination will be considered in the case clinically adverse outcome shown by CPC and mRS can be shown in the Xe+MTH group after interim analysis.

9 QUALITY CONROL AND QUALITY ASSURANCE

9.1 Information of study personnel and training

Study personnel will be appropriately informed of the nature and conduct of the study. All study physicians are experienced specialists in anesthesiology and intensive care. One of the study physicians (Ruut Laitio) is experienced in delivering xenon to man and she will train all study physicians to conduct this phase of the study. The investigators have extensive experience in running complex trials in the ICU.

9.2 Monitoring

Data and Safety Monitoring Plan will be prepared and a Data and Safety Monitoring Board (i.e. independent safety committee) will be established according to the relevant SOPs of Turku CRC and that the Plan and the composition of the Board will be communicated with the Ethics Committee of the Hospital District of Southwest Finland before commencement of this study.

(Amendment 5.12 see Summary) The independent safety committee conducted by Turku CRC and chaired by Päivi Rautava, M.D, PhD, will be responsible for both centres. Turku CRC will carry out monitoring in Turku University Hospital and the Clinical Research Institute Helsinki University Central Hospital Ltd. (HYKS-instituutti) will be responsible for monitoring patients enrolled in Helsinki University Hospital.

In addition, the Ethics Committee will be informed about outcomes related to the responsibilities of the Data and Safety Monitoring Board and about other responsibilities defined by ICH GCP.

The study monitor will visit the study centre regularly as agreed by the principal investigator. The study monitor will ensure that the study complies with the good clinical practice (GCP) and applicable regulatory requirements and that the protocol is followed in all aspects, accurate recording of results, reporting of AEs, drug accountability and record keeping.

Furthermore, it will be verified that the clinical facilities remain adequate, and that the CRFs correspond with source data. For this purpose the study monitor will be allowed direct access to hospital or patient records of the study subject, original laboratory data etc. relevant to the study.

It is essential that the investigator and other relevant members of the study centre team are available during the monitoring visits and inspections, and that they devote sufficient time to these processes.

9.3 Protocol amendments

All possible protocol amendments will be provided in writing and consequently sent to the local EC. If other than administrative changes will be made to the protocol, the study will be discontinued until approval by the local EC.

10 DATA HANDLING AND RECORD KEEPING

(Amendment 5.13 see Summary) The data obtained in Helsinki University Hospital will be transported to Timo Laitio, the principal investigator and person in charge of Xe-Hypotheca trial. Address: Department of Anaesthesiology, Intensive Care, Emergency Care and Pain Medicine, Turku University Hospital, Turku, Finland, P.O.X. 52, FIN-20521, Turku, Finland. A safety copy of

all data obtained in Helsinki University Hospital will be stored at ICU and HUSRöntgen (i.e. MRI data).

10.1 Case Report Forms

Relevant baseline information and follow-up monitoring will be manually recorded on specifically designed CRFs by study nurse or by the investigators of the ICU team. These same persons are allowed to make corrections to the CRF if necessary. After the correction, the original writing must be visible, and the correction must be dated and marked with one's initials. The following is considered as a source data:

- Electronic data collection and capture (see details in 10.2.)
- Original patient documents and reports made by nurses and physicians during the hospital stay(s) and during the follow-up
- X-rays, brain CT, MRIs and electronic PET data

10.2 Electronic data collection and capture

Collection:

- Original continuous electrocardiogram (3 channels), arterial blood pressure, and pulse oximetry will be recorded during the treatments and displayed on a multichannel monitor (GE Healthcare S/5 Compact Monitor, E-PRESTIN module, ECG cable, and S/5 Collect software on a laptop PC). All data is transferred/copied to PC-workstations for further analysis.

Captured from the ICU database:

- Ventilatory parameters (i.e. tidal volume, respiratory frequency, minute volume),
- Hemodynamic: central venous pressure and blood pressure.
- Bladder and oesophageal temperature
- The concentrations of end tidal xenon and oxygen are displayed electronically and continuously monitored, recorded and stored.
- All laboratory results
- Use and dosage of all medication
- All medical reports written or dictated by nurses and physicians

10.3 Data management

Manually recorded CRFs will be stored at the Department of Anesthesiology and Intensive Care of the Turku University Hospital. Chart files containing the continuously measured clinical data will be stored on a PC workstation, and will be packed up on CD-ROMs. The data which have been collected before a possible withdrawal will be used for study purposes. All patient files, including ethical committee approvals and amendments, all source documents and case report copies, PET film hard copies, and patient informed consent forms will be stored for a minimum of 15 years. Data Protection: We will follow the guidelines in the European Clinical Trial Directive regarding protecting the identity of the patients.

10.4 Study subject register

Each study patient is identified by a patient code (i.e. first two initials of first and last name and subject number). The patient identity (initials and screening/subject number, the subject's first name, last name, date of birth, social security number) is recorded on a separate register (i.e. patient identity file) which can be accessed only by the PI with a password. Study subject records, subject screening log, all original signed informed consent forms, and CRFs will be kept in the Investigator's study file to enable the follow-up assessements by safety committee and monitor. Investigator's study file can be accessed only by the PI and biostatistician with a password.

The study subject code (initials and screening/subject number), date of informed consent, date of entry or date of exclusion and the reason for exclusion for those recruited for screening (i.e. a patient with a decision to start MHT) but not fulfilling the inclusion criteria and therefore excluded will be recorded in the subject screening log. Also, all those patients who have been admitted to ICU after OHCA but will not receive MHT, will be recorded in the list with a reason for not to be treated with MHT.

11 ETHICS

11.1 Ethical review

Prior to commencement of this investigation, the study protocol, subject information and informed consent form will be submitted for approval to the Ethics Committee (EC) of the Hospital District of Southwest Finland. The principal investigator (PI) is responsible for obtaining approval of the EC for the study protocol including its appendices and for keeping the EC informed of any SAEs and amendments as requested by the EC. The PI should file all correspondence with the EC in the Investigator's Study File.

11.2 Ethical conduct of the study

The study will be conducted in accordance with the current revision of Declaration of Helsinki guiding physicians in medical research involving human subjects.

The issue of informed consent from a comatose patient is a vexing matter that has engendered considerable debate regarding the conduct of trials related to brain protection where the window of opportunity to initiate a beneficial intervention is quite small. The laws regarding informed consent from a comatose patient vary across different countries. We propose to undertake due diligence of the pertinent laws in Finland relating to informed consent of a comatose patient.

The current study treatment i.e., the combination of inhaled Xenon and endovascular hypothermia has not been administered to any patient, or a group of patients, or other individuals prior this trial. Therefore, a serious attention to the safety of the patients has been and will be paid during this trial. Especially, investigators involved in administering xenon to the patient will be carefully trained by an investigator (RL) who has an earlier experience in administering xenon to man. This study will be conducted in accordance with GCP and with good scientific practice (GSP). An independent safety committee will be involved throughout the trial. All investigations performed and the methods employed in this clinical trial, except administering xenon to a patient needing intensive care, are in routine use in the ICU of Turku University Hospital. Therefore, it is crucial that all investigators who will be involved in taking care of the patients in the ICU as well as during the imaging procedures are experienced anesthesiologists and/or intensivists. Also, technical and other supporting staff of Turku PET Centre and the department of radiology are well experienced to perform PET and MRI studies.

11.3 Subject information and informed consent

The written informed consent will be obtained from the next of kin as soon as possible after the patient has arrived to the hospital. Next of kin will be provided with appropriate information on the investigation, the way it is conducted, its risks and its aims. They will be allowed sufficient time to acquaint them selves with the subject information prior to obtaining the written informed consent.

12 FINANCING AND INSURANCE

This study has received a 4-year (2009-2012) grant from the Academy of Finland. An additional funding has been applied form the Sigrid Juselius Foundation. Further grants will be applied (e.g. EVO) as considered necessary.

All patients will be insured by the "Insurance against medicine-related injuries" (Lääkevahinkovakuutus).

13 STUDY REPORT AND PUBLICATION(S)

All effort will be put on eventually publishing the results in peer-reviewed high impact medical journals. The authors of such publications will be determined in joint agreement by the investigators.

14 ARCHIVING

The principal investigator is responsible for archiving all study related material according to current regulations.

15 STUDY AGREEMENT

(Amendment 5.14 see Summary) A study agreement will be signed by the principal investigators of both participating centres, and by the managing director Seppo Pakkala of Clinical Research Institute Helsinki University Central Hospital Ltd. (HYKS-instituutti) and director of Hospital District of Southwest Finland before the study can be started in Helsinki University Hospital.

16 REFERENCES

1. Katz E. *Rev Med Suisse* 2007;3(96):302-07; **2.** Bernard SA *et al. Ann Emerg Med* 1997; 30:146–53; **3.** Hypothermia after Cardiac Arrest Study Group. *N Engl J Med* 2002;346:549-56; **4.** Bernard SA *et al. N Engl J Med* 2002;346:557-63; **5.** Holzer M *et alCrit Care Med* 2005;33(2):414-18; **6.** Trudell et al. Anesth Analg 1998;87:239-41; **7.** Franks NP *et al. Nature* 1998;396:324; **8.** Wilhelm S *et al. Anesthesiology* 2002;96(6):1485-91; **9.** Ma D *et al. Anesthesiology* 2003;99(3):748-51; **10.** Homi HM *et al. Anesthesiology* 2003;99(4):876-81; **11.** Petzelt C *et al. Life Sci* 2003;72(17):1909-18; **12.** Petzelt C *et al. BMC Neurosci* 2004;5(1):55; **13.** Dingley J *et al. Stroke* 2006;37(2):501-06; **14.** Ma D *et al. Anesthesiology* 2003;98(3):690-98; **15.** Fries M *et al. Crit Care Med* 2008;36:2420-26; **16.** Ma D *et al. Ann Neurol* 2005; 58(2):182-93; **17.** Heurteaux C *et al.* EMBO J 2004;23:2684-95; **18.** Plested AJ, *et al. Anesthesiology* 2004;100(2):347-58; **19.** Dinse A *et al. Br J Anaesth* 2005;94(4):479-85; **20.** Kilic U *et al. FASEB J* 2005;19(14):2026-28; **21.** Ma *et al.* J Cerebral Blood Flow & Metabolism 2006;26:199-208; **22.** Rex S *et al. Anesthesiology* 2006;105(5):936-43; **23.** Laitio R et al. Anesthesiology 2007;106:1128-33; **24.** Laitio R. Doctoral Thesis, Ser. D-TOM 783.

Medica-Odontologica. Annales Universitatis Turkuensis. 112 pages. 2007; **25.** Langsjo JW *et al. Anesthesiology* 2004; 100:1065–71; **26.** Kaisti KK *et al. Anesthesiology* 2003;99:603–13; **27.** Gingsberg MDM. New York: Marcel Dekker 2005;85-140; **28.** Martin JL *et al. Br J Anaesth* 2007;98(2):236-40; **29.** Hobbs C *et al. Stroke 2008;39:* **30.** Sanders RD *et al. Br J Anaesth* 2003;91(5):709-17; **31.** Burov NE *et al. Anesteziol Reanimatol* 1999;6:56-60; **32.** Rossaint R *et al. Anesthesiology* 2003;98(1):6-13; **33.** Stowe DF *et al. Anesthesiology* 2000;92:516–22; **34.** Schroth SC *et al. Anesthesiology* 2002;96: 422–27;**35.** Baumert JH *et al. Acta Anaesthesiol Scand* 2005;49:743-49; **36.** Preckel B *et al. Anesth Analg* 2000;91(6):1327-32; **37.** Bedi A *et al. Crit Care Med* 2003;31(10):2470-77; **38.** Hillman J *et al. Br J Neurosurg* 2005;19(5):395-401; **39.** Lockwood GG *et al. Anesthesiology* 2006;104(3):458-65; **40.** Laitio R *et al.* Anesthesiology 2008;108:63-70; **41**. Langhelle A *et al*. Resuscitation 2005; 66: 271–283; **42**. Baelieu C. *NMR Biomed* 15:435-455:2002)*;* **43**. Kim DG et al. *NeuroRehabilitation* 22:273-277, 2007); **44.** Ross BD *et al. J Neurotrauma* 2001; 18: 977; **45.** Garnett MR *et al. Brain* 2000; 123:2046-54; **46.** Demougeot C *et al. J. Neurochem* 2004;90:776–83; **47.** Signoretti S. *J Neurotrauma* 2001;18:977. **48.** Pan JW, Takahasi 2005. **49.** Patel TB, Clark JB. *Biochem J* 1979;184:539–46; **50.** Bates T *et al.* Neuroreport 1996;7:1397– 4100; **51.** Pereira AC *et al. Stroke* 1999;30:1577–82; **52.** Coon AL *et al. Am J Neuroradiol* 2006;27:1053-58; **53.** Barker PB. In: Gillard JH, Waldman AD, Barker PB, editors. Cambridge: Cambridge University Press;2005.7–26; **54.** Basser PJ et al. Biophysical journal 1994; 66(1): 259-267 ; **55.** Jeurissen B. et al. Human Brain Mapping 2013; 34(11):2747-2766; **56.** Tournier J. et al. NeuroImage 2004; 23(3):1176-1185 ; **57.**Tournier J. et al. NeuroImage 2007; 35(4):1459-1472 ; **58.** Jeurissen B. et al. Human brain mapping 2011; 32(3): 461-479; **59.**Bullmore E. & Sporns, O. Nature Reviews Neuroscience 2009; 10(3): 186-198; **60.** van den Heuvel MP et al. The Journal of Neuroscience 2010; 30(47):15915-15926; **61.** Caeyenberghs K. et al. Brain Structure and Function 2012, 1-17; **62.** Jones DK. et al. Magn Reson Med 1999, 42; **63.** Andersson JL. et al. Neuroimage 2003; 20(2): 870-888 ; **64**. Laitio et al. Anesthesiology 2000; 93:69-80; **65**. Task Force of the ESC. Special Report. Circulation 1996; 93:1043-65; **66.** Iellamo F et al. J Hypertens. 1996; 14(9):1099-104; **67**. Iellamo F et al. Circulation. 1999;6;100(1):27-32.

17 ABBREVIATIONS AND DEFINITION OF TERMS

ADNP	Activity dependent neuroprotective protein
AMPA	α-amino-3-hydroxine-5-methyl-4-isoxazoleproprionate
BRS	Baroreflex sensitivity
DTI	Diffusion tensor magnetic resonance imaging
[^{18}F]-EF5	^{18}F-2-(2-nitro-1H-imidazol-1-yl)-N-(2,2,3,3,3-pentafluoropropyl)-acetamide
EPO	Erythropoeitin
FA	Fractional anisotrophy
HIF 1a	Hypoxia inducible factor 1
1H-MRSI	Proton magnetic resonance spectroscopy
LV	Left ventricular
ICU	Intensive care unit
OHCA	Out-of-hospital cardiac arrest
MAC	Minimal alveolar concentration
MD	Mean Diffucivity
MHT	Mild hypothermia treatment
MR	Magnetic resonance
MRGlu	Regional cerebral metabolic rate of glucose
MRI	Magnetic resonance imaging
NMDA	N-metyl-D-aspartate
NAA	N-acetylaspartate
NSE	Neuron specific enolase
PET	Positron emission tomography
ROSC	Return of spontaneous circulation (ROSC)
VEGF	Vascular endothelial growth factor
Xe	Xenon

18 SUMMARY OF THE AMENDMENTS

AMENDMENT	DATE	Section	Regarding	Earlier protocol	Amended protocol	Reason
No 1.1	10.6.2009	**6.2**	Patient care	A research nurse will be present if needed. Study physician can be reached by telephone.	The study physician and research nurse will always be available during the MHT treatments. Study physician cannot make any clinical decisions	Safety and to avoid bias
No 2.1	16.10.2009	**4.4**	Exclusion criteria	Exclusion criteria: Admission B-Glucose > 15 mmol/litre	Omitted	Weak evidence
2.2		**4.4**	Exclusion criteria	Occurrence of cardiac arrest after the arrival of emergency medical personnel	Omitted	Clerical error
2.3		**6.1.2**	MRI timing	The second MRI will be performed 10 days after OHCA.	Second MRI will be performed 10 ± 2 days after OHCA	Safety
2.4		**6.1.2**	MRI protocol		Brain temperature is measured noninvasively using proton MR spectroscopy	Study spin-off **(was not feasible)**
2.5		**6.1.2**	MRI protocol		A new method of Tract Based Spatial Statistics (TBSS) with diffusion tensor magnetic resonance imaging will be applied.	New method
2.6		**6.1**	Patient monitoring		Transcranial doppler (TCD) imaging will be performed to all feasible patients.	Study spin-off **(was not feasible; not implemented)**
2.7			Investigators		MRI physicist PhD Jani Saunavaara has been added to the investigator list.	New investigator
2.8		**6.1.2**	Laboratory samples	TNT, Ck-mb and NSE are obtained at arrival to ICU, at 24 hours, at 48 hours and at 72 hours after arrival to the hospital.	TNT, Ck-mb and NSE are obtained at arrival to ICU, and 24 hours, 48 hours and 72 hours after OHCA.	Previous literature
2.9		**4.3**	Inclusion criteria	Inclusion criteria: age 18 – 75 years	Inclusion criteria age: 18-80 years	Weak evidence
No 3.1	21.2.2011	**6.1**	Ventilatory care		Transpulmonic pressure measurements during treatments	Study spin-off **(was not feasible; not implemented)**

3.2		**6.1**	Monitoring		The effect of xenon inhalation on cerebral carbon dioxide reactivity via trans cranial doppler	Study spin-off **(was not feasible not; implemented)**
3.3			Investigators		Neuroradiologist Sami Virtanen has been added to the investigator list	New investigator
No 4.1	29.12.2011	**6.1.3**	PET protocol	PET will be performed within 24 hours after completion of rewarming: Imaging hypoxic but viable brain tissue with hypoxia tracer [18F]-EF5	Brain PET will be performed 5±2 days after OHCA to image microglia cell activation Imaging hypoxic but viable brain tissue with hypoxia tracer [18F]-EF5	Study spin-off **(was not feasible)** , logistical rearrangements
4.2		**6.1.3**	PET protocol	PET protocol	PET protocol rewritten	Study spin-off **(was not feasible)**
No 5.1	22.3.2012		Study sites		List of investigators and study sites as a new recruiting center Helsinki University Hospital became involved.	Recruitment
5.2		**6.1.3**	PET protocol	Brain PET will be performed 5±2 days after OHCA	Brain PET will be performed 5±2 days after OHCA (not to be performed in Helsinki).	Availability **(was not feasible, by the end of patient recruitment PET imaging was undertaken in 5 patients)**
5.3		**3.1**	Study design	A phase II, randomized, controlled two armed parallel follow-up study.	A two-centre, phase II, randomized, controlled two armed parallel follow-up study.	New recruiting center
5.4		**3.2**	Randomisation	Randomisation will be performed in random blocks of several sizes.	Randomisation will be performed in random blocks of several sizes. The randomisation will be performed independently and separately at each centre.	New recruiting center
……5.5		**5.1**	Patient care	0.5°C/hour rewarming of the patients is allowed to a temperature of 36.5-37.0 °C	A maximum rate 0.5°C/hour rewarming of the patients is allowed to a temperature of 36.5-37.0 °C	Previous literature
5.6		**5.4.3**	Patient care	Serum glucose will be kept between 4.5-6.5 mmol/l	Serum glucose will be kept between 5.0-8.0 mmol/l	New evidence from literature

5.7	**6**	Data analysis		All data obtained in Helsinki University Hospital will be transported to Turku University Hospital, and analyzed in Turku	New recruiting center
5.8	**6.1**	PET and TCD protocol		PET, TCD and carbon dioxide reactivity not to be assessed in Helsinki	Study spin-off, Turku **(was not feasible)**
5.9	**6.2**	Safety	An independent safety committee and a monitor will be involved throughout the trial.	An independent safety committee conducted by Turku CRC will be responsible for both centres. A monitor from both centres will be involved throughout the trial. Turku CRC will carry out monitoring in Turku and the Clinical Research Institute Helsinki University Central Hospital Ltd. will be responsible for monitoring patients enrolled in Helsinki.	Safety
5.10	**6.1.6**	Laboratory samples	P-TnT, P-NSE and P-CK-MBm will be determined by TYKSLAB	P-TnT, P-NSE and P-CK-MBm will be determined by TYKSLAB in Turku University Hospital and by HUSLAB in Helsinki University Hospital	New recruiting center
5.11	**6.1.6**	Laboratory samples		The blood and urine samples obtained and stored in -70 ºC in HUSLAB will be transported to Turku CRST for later determination of catecholamines.	New recruiting center
5.12	**9**	Study monitoring		The independent safety committee conducted by Turku CRC and chaired by Päivi Rautava, M.D, PhD, will be responsible for both centres. Turku CRC will carry out monitoring in Turku University Hospital and the Clinical Research Institute Helsinki University Central Hospital Ltd. will be responsible for monitoring patients enrolled in Helsinki University Hospital.	New recruiting center

5.13		**10**	Data handling		The data obtained in Helsinki University Hospital will be transported to Timo Laitio, the principal investigator and person in charge of Xe-Hypotheca trial.	New recruiting center
5.14		**15**	Study agreement		A study agreement will be signed by the principal investigators of both participating centres, and by the managing director Seppo Pakkala of Clinical Research Institute Helsinki University Central Hospital Ltd. (HYKS-instituutti) and director of Hospital District of Southwest Finland before the study can be started in Helsinki University Hospital.	New recruiting center
No 6.1	28.1.2013		Investigators		Updated list of investigators	
6.2		**Synopsis**	MRI protocol	First magnetic resonance imaging (MRI) of the brain will be performed within 16 hours after completion of rewarming and the 2nd MRI 10 days after OHCA	First magnetic resonance imaging (MRI) of the brain will be performed within 16 hours after completion of rewarming, the 2nd MRI 10±2 days and the 3rd 3-3.5 years after OHCA	New method
6.3		**Synopsis**	MRI protocol		Volumetric changes of the gray matter, white matter, and cerebro-spinal fluid spaces: Advanced post-processing method	New method **(study spin-off)**
6.4		**Synopsis**	MRI protocol		Estimation of hemosiderin and local neurodegeneration: Susceptibility-weighted imaging (SWI), will be used only in the 3rd MRI	New method **(study spin-off)**
6.5		**Synopsis**	Follow-up	Long-term outcome: 5years after cardiac arrest	Long-term outcome: 3-3.5 years after cardiac arrest	To avoid loss of follow-up

6.6		**2.1**	MRI protocol		Added protocol for mri verified damage. Late manifestations: white matter degeneration, volumetric changes, atrophy of the both white and gray matter and dilatation of the cortical cerebro-spinal fluid spaces and ventricles.	Exploratory plan
6.7		**6.1.2**	MRI protocol		An advanced post-processing method is applied in the volumetric analysis and carried out in all obtained MRIs.	Exploratory plan **(study spin-off)**
6.8		**3.3**	Follow-up	The long-term outcome will be assessed at 5 years after the cardiac arrest by telephone, or by seeing the patient at home or at study physician's appointment.	The long-term outcome will be assessed at 3-3.5 years after the cardiac arrest by telephone, or by seeing the patient at home or at study physician's appointment.	To avoid loss of follow-up
No 7.	24.2.2014	**6.1.2**	MRI protocol		Constrained spherical deconvolution (CSD) and whole brain tract networks graph theoretical analysis added as MRI methods	Study spin-off
