# Supplementary material for: A metabolic profile of xenon and metabolite associations with 6-month mortality after out-of-hospital cardiac arrest: A post-hoc study of the randomised Xe-Hypotheca trial
Source: PLoS One. 2024 Jun 4;19(6):e0304966. doi: 10.1371/journal.pone.0304966 (PMC11149864; doi:10.1371/journal.pone.0304966)
Supplement: S1 Fig — (DOCX) [file pone.0304966.s003.docx]

**
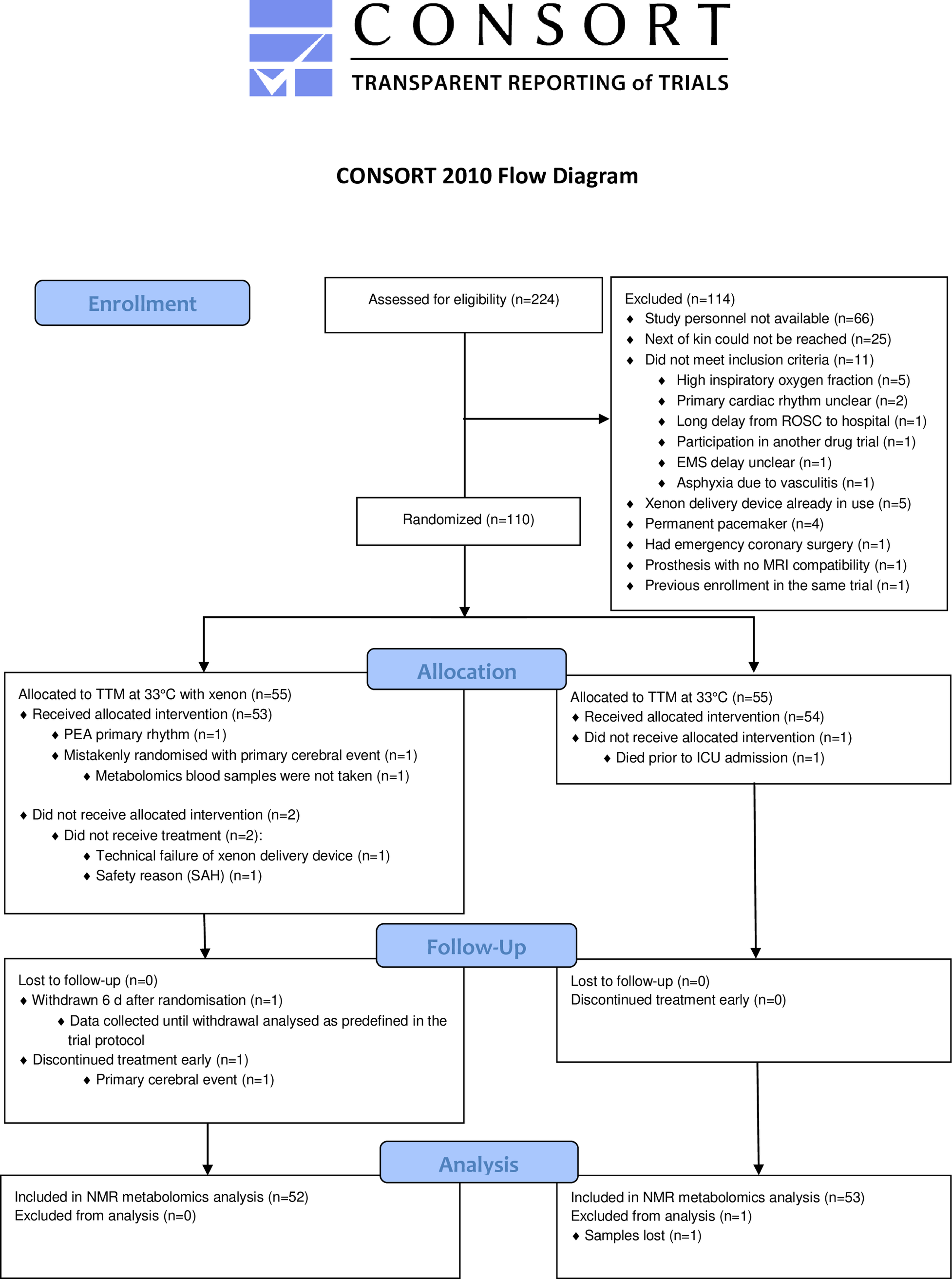
**

**S1 Fig CONSORT flow diagram.** Abbreviations: EMS, emergency medical service; ICU, intensive care unit; NMR, nuclear magnetic resonance spectroscopy; MRI, magnetic resonance imaging; PEA, pulseless electrical activity; ROSC, return of spontaneous circulation; SAH, subarachnoid haemorrhage; TTM, targeted temperature management.
