# Supplementary material for: A metabolic profile of xenon and metabolite associations with 6-month mortality after out-of-hospital cardiac arrest: A post-hoc study of the randomised Xe-Hypotheca trial
Source: PLoS One. 2024 Jun 4;19(6):e0304966. doi: 10.1371/journal.pone.0304966 (PMC11149864; doi:10.1371/journal.pone.0304966)
Supplement: S1 Table — (DOCX) [file pone.0304966.s004.docx]

**S1 Table Metabolite abbreviations in alphabetical order**

| **Abbreviation** | **Name** |
| --- | --- |
| AcAce | Acetoacetate |
| Ace | Acetate |
| Ala | Alanine |
| Alb | Albumin |
| ApoA1 | Apolipoprotein A-1 |
| ApoB | Apolipoprotein B |
| ApoB-ApoA1 | Ratio of apolipoprotein B to apolipoprotein A-1 |
| bOHBut | 3-hydroxybutyrate |
| Cit | Citrate |
| Crea | Creatinine |
| DHA-FA | Ratio of 22:6 docosahexaenoic acid to total fatty acids |
| DHA | 22:6 docosahexaenoic acid |
| EstC | Esterified cholesterol |
| FAw3-FA | Ratio of omega-3 fatty acids to total fatty acids |
| FAw3 | Omega-3 fatty acids |
| FAw6-FA | Ratio of omega-6 fatty acids to total fatty acids |
| FAw6 | Omega-6 fatty acids |
| FreeC | Free cholesterol |
| Glc | Glucose |
| Gln | Glutamine |
| GlycA | Glycoprotein acetylation |
| HDL-C | Total cholesterol in HDL |
| HDL-D | Mean diameter of HDLDL particles |
| HDL-TG | Triglycerides in HDL |
| HDL2-C | Total cholesterol in HDL2 |
| HDL3-C | Total cholesterol in HDL3 |
| His | Histidine |
| IDL-C | Total cholesterol in IDL particles |
| IDL-CE | Cholesterol esters in IDL particles |
| IDL-FC | Free cholesterol in IDL particles |
| IDL-L | Total lipids in IDL particles |
| IDL-P | Concentration of IDL particles |
| IDL-PL | Phospholipids in IDL particles |
| IDL-TG | Triglycerides in IDL particles |
| Ile | Isoleucine |
| L-HDL-C | Total cholesterol in large HDL particles |
| L-HDL-CE | Cholesterol esters in large HDL particles |
| L-HDL-FC | Free cholesterol in large HDL particles |
| L-HDL-L | Total lipids in large HDL particles |
| L-HDL-P | Concentration of large HDL particles |
| L-HDL-PL | Phospholipids in large HDL particles |
| L-HDL-TG | Triglycerides in large HDL particles |
| L-LDL-C | Total cholesterol in large LDL particles |
| L-LDL-CE | Cholesterol esters in large LDL particles |
| L-LDL-FC | Free cholesterol in large LDL particles |
| L-LDL-L | Total lipids in large LDL particles |
| L-LDL-P | Concentration of large LDL particles |
| L-LDL-PL | Phospholipids in large LDL particles |
| L-LDL-TG | Triglycerides in large LDL particles |
| L-VLDL-C | Total cholesterol in large VLDL particles |
| L-VLDL-CE | Cholesterol esters in large VLDL particles |
| L-VLDL-FC | Free cholesterol in large VLDL particles |
| L-VLDL-L | Total lipids in large VLDL particles |
| L-VLDL-P | Concentration of large VLDL particles |
| L-VLDL-PL | Phospholipids in large VLDL particles |
| L-VLDL-TG | Triglycerides in large VLDL particles |
| LA-FA | Ratio of 18:2 linoleic acid to total fatty acids |
| LA | 18:2 linoleic acid |
| Lac | Lactate |
| LDL-C | Total cholesterol in LDL |
| LDL-D | Mean diameter of LDL particles |
| LDL-TG | Triglycerides in LDL |
| Leu | Leucine |
| M-HDL-C | Total cholesterol in medium HDL particles |
| M-HDL-CE | Cholesterol esters in medium HDL particles |
| M-HDL-FC | Free cholesterol in medium HDL particles |
| M-HDL-L | Total lipids in medium HDL particles |
| M-HDL-P | Concentration of medium HDL particles |
| M-HDL-PL | Phospholipids in medium HDL particles |
| M-HDL-TG | Triglycerides in medium HDL particles |
| M-LDL-C | Total cholesterol in medium LDL particles |
| M-LDL-CE | Cholesterol esters in medium LDL particles |
| M-LDL-FC | Free cholesterol in medium LDL particles |
| M-LDL-L | Total lipids in medium LDL particles |
| M-LDL-P | Concentration of medium LDL particles |
| M-LDL-PL | Phospholipids in medium LDL particles |
| M-LDL-TG | Triglycerides in medium LDL particles |
| M-VLDL-C | Total cholesterol in medium VLDL particles |
| M-VLDL-CE | Cholesterol esters in medium VLDL particles |
| M-VLDL-FC | Free cholesterol in medium VLDL particles |
| M-VLDL-L | Total lipids in medium VLDL particles |
| M-VLDL-P | Concentration of medium VLDL particles |
| M-VLDL-PL | Phospholipids in medium VLDL particles |
| M-VLDL-TG | Triglycerides in medium VLDL particles |
| MUFA | Monounsaturated fatty acids 16:1. 18:1 |
| MUFA-FA | Ratio of monounsaturated fatty acids to total fatty acids |
| PC | Phosphatidylcholine and other cholines |
| Phe | Phenylalanine |
| PUFA-FA | Ratio of polyunsaturated fatty acids to total fatty acids |
| PUFA | Polyunsaturated fatty acids |
| Remnant-C | Remnant cholesterol (non-HDL. non-LDL -cholesterol) |
| S-HDL-C | Total cholesterol in small HDL particles |
| S-HDL-CE | Cholesterol esters in small HDL particles |
| S-HDL-FC | Free cholesterol in small HDL particles |
| S-HDL-L | Total lipids in small HDL particles |
| S-HDL-P | Concentration of small HDL particles |
| S-HDL-PL | Phospholipids in small HDL particles |
| S-HDL-TG | Triglycerides in small HDL particles |
| S-LDL-C | Total cholesterol in small LDL particles |
| S-LDL-CE | Cholesterol esters in small LDL particles |
| S-LDL-FC | Free cholesterol in small LDL particles |
| S-LDL-L | Total lipids in small LDL particles |
| S-LDL-P | Concentration of small LDL particles |
| S-LDL-PL | Phospholipids in small LDL particles |
| S-LDL-TG | Triglycerides in small LDL particles |
| S-VLDL-C | Total cholesterol in small VLDL particles |
| S-VLDL-CE | Cholesterol esters in small VLDL particles |
| S-VLDL-FC | Free cholesterol in small VLDL particles |
| S-VLDL-L | Total lipids in small VLDL particles |
| S-VLDL-P | Concentration of small VLDL particles |
| S-VLDL-PL | Phospholipids in small VLDL particles |
| S-VLDL-TG | Triglycerides in small VLDL particles |
| Serum-C | Serum total cholesterol |
| Serum-TG | Serum total triglycerides |
| SFA | Saturated fatty acids |
| SFA-FA | Ratio of saturated fatty acids to total fatty acids |
| SM | Sphingomyelins |
| TG-PG | Ratio of total triglycerides to phosphoglycerides |
| TotCho | Total cholines |
| TotFA | Total fatty acids |
| TotPG | Total phosphoglycerides |
| Tyr | Tyrosine |
| UnSat | Estimated degree of unsaturation |
| Val | Valine |
| VLDL-C | Total cholesterol in VLDL |
| VLDL-D | Mean diameter of VLDL particles |
| VLDL-TG | Triglycerides in VLDL |
| XL-HDL-C | Total cholesterol in very large HDL particles |
| XL-HDL-CE | Cholesterol esters in very large HDL particles |
| XL-HDL-FC | Free cholesterol in very large HDL particles |
| XL-HDL-L | Total lipids in very large HDL particles |
| XL-HDL-P | Concentration of very large HDL particles |
| XL-HDL-PL | Phospholipids in very large HDL particles |
| XL-HDL-TG | Triglycerides in very large HDL particles |
| XL-VLDL-C | Total cholesterol in very large VLDL particles |
| XL-VLDL-CE | Cholesterol esters in very large VLDL particles |
| XL-VLDL-FC | Free cholesterol in very large VLDL particles |
| XL-VLDL-L | Total lipids in very large VLDL particles |
| XL-VLDL-P | Concentration of very large VLDL particles |
| XL-VLDL-PL | Phospholipids in very large VLDL particles |
| XL-VLDL-TG | Triglycerides in very large VLDL particles |
| XS-VLDL-C | Total cholesterol in very small VLDL particles |
| XS-VLDL-CE | Cholesterol esters in very small VLDL particles |
| XS-VLDL-FC | Free cholesterol in very small VLDL particles |
| XS-VLDL-L | Total lipids in very small VLDL particles |
| XS-VLDL-P | Concentration of very small VLDL particles |
| XS-VLDL-PL | Phospholipids in very small VLDL particles |
| XS-VLDL-TG | Triglycerides in very small VLDL particles |
| XXL-VLDL-C | Total cholesterol in chylomicrons and extremely large VLDL particles |
| XXL-VLDL-CE | Cholesterol esters in chylomicrons and extremely large VLDL particles |
| XXL-VLDL-FC | Free cholesterol in chylomicrons and extremely large VLDL particles |
| XXL-VLDL-L | Total lipids in chylomicrons and extremely large VLDL particles |
| XXL-VLDL-P | Concentration of chylomicrons and extremely large VLDL particles |
| XXL-VLDL-PL | Phospholipids in chylomicrons and extremely large VLDL particles |
| XXL-VLDL-TG | Triglycerides in chylomicrons and extremely large VLDL particles |
