# Supplementary material for: A metabolic profile of xenon and metabolite associations with 6-month mortality after out-of-hospital cardiac arrest: A post-hoc study of the randomised Xe-Hypotheca trial
Source: PLoS One. 2024 Jun 4;19(6):e0304966. doi: 10.1371/journal.pone.0304966 (PMC11149864; doi:10.1371/journal.pone.0304966)
Supplement: S2 Table — (DOCX) [file pone.0304966.s005.docx]

**S2 Table** Metabolite associations with 6-month mortality, all measured metabolites. Data is reported as adjusted hazard ratio (HR) with 95 % confidence interval (95%-CI), unadjusted and adjusted p-value. Based on principal component analysis over 95% of variation in metabolomic data was explained by 14 components in all three time points, thus the p-values have been adjusted by a factor of 14 for multiple testing, an alpha threshold of 0.05 was used. Statistically significant results are marked with *.

| **Concentration of lipoprotein particles** | | |  |  |  |
| --- | --- | --- | --- | --- | --- |
| **Metabolite** | **Timepoint** | **HR** | **95%-CI** | **Unadjusted p-value** | **Adjusted p-value** |
| XXL-VLDL-P | 1 | 0.58 | (95%-CI 0.35 to 0.96) | 0.033 | 0.465 |
|  | 2 | 0.82 | (95%-CI 0.55 to 1.22) | 0.324 | 1 |
|  | 3 | 1.18 | (95%-CI 0.72 to 1.92) | 0.51 | 1 |
| XL-VLDL-P | 1 | 0.89 | (95%-CI 0.53 to 1.5) | 0.675 | 1 |
|  | 2 | 0.77 | (95%-CI 0.42 to 1.39) | 0.381 | 1 |
|  | 3 | 1.14 | (95%-CI 0.66 to 1.98) | 0.639 | 1 |
| L-VLDL-P | 1 | 0.87 | (95%-CI 0.53 to 1.44) | 0.599 | 1 |
|  | 2 | 0.81 | (95%-CI 0.54 to 1.2) | 0.29 | 1 |
|  | 3 | 0.94 | (95%-CI 0.54 to 1.64) | 0.827 | 1 |
| M-VLDL-P | 1 | 0.61 | (95%-CI 0.39 to 0.94) | 0.025 | 0.348 |
|  | 2 | 0.81 | (95%-CI 0.54 to 1.21) | 0.302 | 1 |
|  | 3 | 0.87 | (95%-CI 0.53 to 1.44) | 0.599 | 1 |
| S-VLDL-P | 1 | 0.64 | (95%-CI 0.38 to 1.06) | 0.081 | 1 |
|  | 2 | 0.74 | (95%-CI 0.49 to 1.11) | 0.143 | 1 |
|  | 3 | 0.94 | (95%-CI 0.58 to 1.51) | 0.799 | 1 |
| XS-VLDL-P | 1 | 0.81 | (95%-CI 0.55 to 1.18) | 0.273 | 1 |
|  | 2 | 0.61 | (95%-CI 0.34 to 1.1) | 0.102 | 1 |
|  | 3 | 0.73 | (95%-CI 0.44 to 1.22) | 0.226 | 1 |
| IDL-P | 1 | 0.77 | (95%-CI 0.53 to 1.13) | 0.187 | 1 |
|  | 2 | 0.64 | (95%-CI 0.39 to 1.07) | 0.09 | 1 |
|  | 3 | 0.56 | (95%-CI 0.32 to 0.99) | 0.048 | 0.67 |
| L-LDL-P | 1 | 0.69 | (95%-CI 0.45 to 1.04) | 0.077 | 1 |
|  | 2 | 0.66 | (95%-CI 0.31 to 1.4) | 0.282 | 1 |
|  | 3 | 0.4 | (95%-CI 0.18 to 0.88) | 0.023 | 0.327 |
| M-LDL-P | 1 | 0.84 | (95%-CI 0.54 to 1.31) | 0.443 | 1 |
|  | 2 | 0.64 | (95%-CI 0.3 to 1.38) | 0.258 | 1 |
|  | 3 | 0.61 | (95%-CI 0.27 to 1.37) | 0.231 | 1 |
| S-LDL-P | 1 | 0.61 | (95%-CI 0.37 to 1.01) | 0.055 | 0.774 |
|  | 2 | 0.71 | (95%-CI 0.29 to 1.76) | 0.457 | 1 |
|  | 3 | 0.49 | (95%-CI 0.19 to 1.27) | 0.143 | 1 |
| XL-HDL-P | 1 | 0.91 | (95%-CI 0.59 to 1.4) | 0.657 | 1 |
|  | 2 | 1.07 | (95%-CI 0.7 to 1.64) | 0.752 | 1 |
|  | 3 | 1.02 | (95%-CI 0.6 to 1.73) | 0.942 | 1 |
| L-HDL-P | 1 | 0.94 | (95%-CI 0.6 to 1.49) | 0.807 | 1 |
|  | 2 | 1.07 | (95%-CI 0.72 to 1.59) | 0.724 | 1 |
|  | 3 | 0.74 | (95%-CI 0.36 to 1.51) | 0.405 | 1 |
| M-HDL-P | 1 | 0.76 | (95%-CI 0.48 to 1.22) | 0.256 | 1 |
|  | 2 | 0.78 | (95%-CI 0.45 to 1.35) | 0.382 | 1 |
|  | 3 | 0.51 | (95%-CI 0.25 to 1.03) | 0.061 | 0.858 |
| S-HDL-P | 1 | 0.8 | (95%-CI 0.51 to 1.25) | 0.326 | 1 |
|  | 2 | 0.64 | (95%-CI 0.4 to 1.03) | 0.067 | 0.935 |
|  | 3 | 0.68 | (95%-CI 0.4 to 1.16) | 0.16 | 1 |
|  |  |  |  |  |  |
|  |  |  |  |  |  |
| **Total lipids in lipoprotein particles** | | |  |  |  |
| **Metabolite** | **Timepoint** | **HR** | **95%-CI** | **Unadjusted p-value** | **Adjusted p-value** |
| XXL-VLDL-L | 1 | 0.58 | (95%-CI 0.35 to 0.96) | 0.034 | 0.475 |
|  | 2 | 0.82 | (95%-CI 0.55 to 1.22) | 0.321 | 1 |
|  | 3 | 1.17 | (95%-CI 0.71 to 1.91) | 0.541 | 1 |
| XL-VLDL-L | 1 | 0.9 | (95%-CI 0.54 to 1.51) | 0.691 | 1 |
|  | 2 | 0.76 | (95%-CI 0.42 to 1.38) | 0.371 | 1 |
|  | 3 | 1.13 | (95%-CI 0.65 to 1.96) | 0.659 | 1 |
| L-VLDL-L | 1 | 0.87 | (95%-CI 0.53 to 1.44) | 0.6 | 1 |
|  | 2 | 0.81 | (95%-CI 0.54 to 1.2) | 0.286 | 1 |
|  | 3 | 0.94 | (95%-CI 0.53 to 1.64) | 0.82 | 1 |
| M-VLDL-L | 1 | 0.61 | (95%-CI 0.39 to 0.93) | 0.023 | 0.329 |
|  | 2 | 0.8 | (95%-CI 0.54 to 1.2) | 0.284 | 1 |
|  | 3 | 0.86 | (95%-CI 0.52 to 1.42) | 0.559 | 1 |
| S-VLDL-L | 1 | 0.64 | (95%-CI 0.39 to 1.05) | 0.08 | 1 |
|  | 2 | 0.73 | (95%-CI 0.48 to 1.1) | 0.128 | 1 |
|  | 3 | 0.92 | (95%-CI 0.57 to 1.48) | 0.73 | 1 |
| XS-VLDL-L | 1 | 0.8 | (95%-CI 0.54 to 1.16) | 0.239 | 1 |
|  | 2 | 0.61 | (95%-CI 0.34 to 1.1) | 0.103 | 1 |
|  | 3 | 0.69 | (95%-CI 0.41 to 1.16) | 0.161 | 1 |
| IDL-L | 1 | 0.76 | (95%-CI 0.52 to 1.12) | 0.168 | 1 |
|  | 2 | 0.64 | (95%-CI 0.38 to 1.06) | 0.085 | 1 |
|  | 3 | 0.54 | (95%-CI 0.3 to 0.96) | 0.036 | 0.504 |
| L-LDL-L | 1 | 0.68 | (95%-CI 0.45 to 1.04) | 0.073 | 1 |
|  | 2 | 0.66 | (95%-CI 0.31 to 1.39) | 0.276 | 1 |
|  | 3 | 0.37 | (95%-CI 0.17 to 0.83) | 0.016 | 0.224 |
| M-LDL-L | 1 | 0.83 | (95%-CI 0.53 to 1.31) | 0.428 | 1 |
|  | 2 | 0.64 | (95%-CI 0.3 to 1.37) | 0.248 | 1 |
|  | 3 | 0.6 | (95%-CI 0.27 to 1.32) | 0.203 | 1 |
| S-LDL-L | 1 | 0.62 | (95%-CI 0.37 to 1.01) | 0.057 | 0.801 |
|  | 2 | 0.71 | (95%-CI 0.28 to 1.77) | 0.459 | 1 |
|  | 3 | 0.48 | (95%-CI 0.19 to 1.24) | 0.128 | 1 |
| XL-HDL-L | 1 | 0.9 | (95%-CI 0.58 to 1.39) | 0.638 | 1 |
|  | 2 | 1.07 | (95%-CI 0.7 to 1.64) | 0.756 | 1 |
|  | 3 | 1.02 | (95%-CI 0.6 to 1.72) | 0.948 | 1 |
| L-HDL-L | 1 | 0.94 | (95%-CI 0.6 to 1.49) | 0.801 | 1 |
|  | 2 | 1.08 | (95%-CI 0.73 to 1.6) | 0.71 | 1 |
|  | 3 | 0.74 | (95%-CI 0.36 to 1.52) | 0.408 | 1 |
| M-HDL-L | 1 | 0.76 | (95%-CI 0.48 to 1.21) | 0.255 | 1 |
|  | 2 | 0.79 | (95%-CI 0.46 to 1.37) | 0.404 | 1 |
|  | 3 | 0.51 | (95%-CI 0.25 to 1.03) | 0.06 | 0.84 |
| S-HDL-L | 1 | 0.79 | (95%-CI 0.51 to 1.23) | 0.303 | 1 |
|  | 2 | 0.63 | (95%-CI 0.39 to 1.02) | 0.062 | 0.869 |
|  | 3 | 0.66 | (95%-CI 0.38 to 1.12) | 0.124 | 1 |
|  |  |  |  |  |  |
|  |  |  |  |  |  |
|  |  |  |  |  |  |
|  |  |  |  |  |  |
|  |  |  |  |  |  |
|  |  |  |  |  |  |
| **Phospholipids in lipoprotein particles** | | |  |  |  |
| **Metabolite** | **Timepoint** | **HR** | **95%-CI** | **Unadjusted p-value** | **Adjusted p-value** |
| XXL-VLDL-PL | 1 | 0.56 | (95%-CI 0.37 to 0.85) | 0.006 | 0.085 |
|  | 2 | 0.83 | (95%-CI 0.58 to 1.18) | 0.295 | 1 |
|  | 3 | 1.18 | (95%-CI 0.71 to 1.96) | 0.516 | 1 |
| XL-VLDL-PL | 1 | 0.89 | (95%-CI 0.54 to 1.45) | 0.635 | 1 |
|  | 2 | 0.73 | (95%-CI 0.42 to 1.25) | 0.25 | 1 |
|  | 3 | 1.06 | (95%-CI 0.62 to 1.82) | 0.833 | 1 |
| L-VLDL-PL | 1 | 0.88 | (95%-CI 0.54 to 1.44) | 0.606 | 1 |
|  | 2 | 0.8 | (95%-CI 0.54 to 1.17) | 0.25 | 1 |
|  | 3 | 0.91 | (95%-CI 0.51 to 1.63) | 0.755 | 1 |
| M-VLDL-PL | 1 | 0.61 | (95%-CI 0.4 to 0.94) | 0.025 | 0.352 |
|  | 2 | 0.81 | (95%-CI 0.54 to 1.19) | 0.281 | 1 |
|  | 3 | 0.85 | (95%-CI 0.52 to 1.39) | 0.513 | 1 |
| S-VLDL-PL | 1 | 0.67 | (95%-CI 0.41 to 1.09) | 0.11 | 1 |
|  | 2 | 0.74 | (95%-CI 0.5 to 1.09) | 0.128 | 1 |
|  | 3 | 0.98 | (95%-CI 0.62 to 1.55) | 0.932 | 1 |
| XS-VLDL-PL | 1 | 0.79 | (95%-CI 0.53 to 1.17) | 0.237 | 1 |
|  | 2 | 0.61 | (95%-CI 0.34 to 1.08) | 0.09 | 1 |
|  | 3 | 0.6 | (95%-CI 0.34 to 1.05) | 0.073 | 1 |
| IDL-PL | 1 | 0.74 | (95%-CI 0.5 to 1.12) | 0.152 | 1 |
|  | 2 | 0.68 | (95%-CI 0.36 to 1.27) | 0.224 | 1 |
|  | 3 | 0.51 | (95%-CI 0.26 to 1) | 0.051 | 0.712 |
| L-LDL-PL | 1 | 0.66 | (95%-CI 0.44 to 1) | 0.049 | 0.687 |
|  | 2 | 0.6 | (95%-CI 0.28 to 1.3) | 0.197 | 1 |
|  | 3 | 0.33 | (95%-CI 0.14 to 0.75) | 0.008 | 0.116 |
| M-LDL-PL | 1 | 0.65 | (95%-CI 0.38 to 1.11) | 0.118 | 1 |
|  | 2 | 0.56 | (95%-CI 0.22 to 1.43) | 0.228 | 1 |
|  | 3 | 0.49 | (95%-CI 0.19 to 1.26) | 0.139 | 1 |
| S-LDL-PL | 1 | 0.54 | (95%-CI 0.32 to 0.89) | 0.017 | 0.236 |
|  | 2 | 0.79 | (95%-CI 0.32 to 1.99) | 0.622 | 1 |
|  | 3 | 0.63 | (95%-CI 0.27 to 1.48) | 0.286 | 1 |
| XL-HDL-PL | 1 | 0.98 | (95%-CI 0.65 to 1.47) | 0.925 | 1 |
|  | 2 | 1.11 | (95%-CI 0.75 to 1.63) | 0.607 | 1 |
|  | 3 | 0.95 | (95%-CI 0.57 to 1.59) | 0.852 | 1 |
| L-HDL-PL | 1 | 0.9 | (95%-CI 0.56 to 1.44) | 0.658 | 1 |
|  | 2 | 1.02 | (95%-CI 0.68 to 1.54) | 0.913 | 1 |
|  | 3 | 0.66 | (95%-CI 0.32 to 1.35) | 0.256 | 1 |
| M-HDL-PL | 1 | 0.82 | (95%-CI 0.52 to 1.29) | 0.39 | 1 |
|  | 2 | 0.84 | (95%-CI 0.49 to 1.45) | 0.539 | 1 |
|  | 3 | 0.49 | (95%-CI 0.22 to 1.1) | 0.084 | 1 |
| S-HDL-PL | 1 | 0.85 | (95%-CI 0.53 to 1.38) | 0.511 | 1 |
|  | 2 | 0.81 | (95%-CI 0.51 to 1.29) | 0.378 | 1 |
|  | 3 | 0.91 | (95%-CI 0.52 to 1.59) | 0.73 | 1 |
|  |  |  |  |  |  |
|  |  |  |  |  |  |
|  |  |  |  |  |  |
|  |  |  |  |  |  |
|  |  |  |  |  |  |
|  |  |  |  |  |  |
| **Total cholesterol in lipoprotein particles** | | |  |  |  |
| **Metabolite** | **Timepoint** | **HR** | **95%-CI** | **Unadjusted p-value** | **Adjusted p-value** |
| XXL-VLDL-C | 1 | 0.65 | (95%-CI 0.43 to 1) | 0.048 | 0.675 |
|  | 2 | 0.81 | (95%-CI 0.55 to 1.19) | 0.292 | 1 |
|  | 3 | 0.98 | (95%-CI 0.54 to 1.77) | 0.951 | 1 |
| XL-VLDL-C | 1 | 0.97 | (95%-CI 0.6 to 1.57) | 0.902 | 1 |
|  | 2 | 0.76 | (95%-CI 0.44 to 1.32) | 0.331 | 1 |
|  | 3 | 1.08 | (95%-CI 0.62 to 1.9) | 0.787 | 1 |
| L-VLDL-C | 1 | 0.9 | (95%-CI 0.55 to 1.45) | 0.654 | 1 |
|  | 2 | 0.83 | (95%-CI 0.57 to 1.19) | 0.305 | 1 |
|  | 3 | 0.94 | (95%-CI 0.54 to 1.66) | 0.841 | 1 |
| M-VLDL-C | 1 | 0.64 | (95%-CI 0.44 to 0.92) | 0.016 | 0.229 |
|  | 2 | 0.72 | (95%-CI 0.5 to 1.03) | 0.07 | 0.975 |
|  | 3 | 0.75 | (95%-CI 0.5 to 1.13) | 0.174 | 1 |
| S-VLDL-C | 1 | 0.68 | (95%-CI 0.42 to 1.1) | 0.115 | 1 |
|  | 2 | 0.57 | (95%-CI 0.32 to 1.01) | 0.054 | 0.759 |
|  | 3 | 0.68 | (95%-CI 0.39 to 1.17) | 0.162 | 1 |
| XS-VLDL-C | 1 | 0.74 | (95%-CI 0.5 to 1.09) | 0.131 | 1 |
|  | 2 | 0.64 | (95%-CI 0.36 to 1.14) | 0.128 | 1 |
|  | 3 | 0.6 | (95%-CI 0.35 to 1.02) | 0.061 | 0.856 |
| IDL-C | 1 | 0.71 | (95%-CI 0.48 to 1.06) | 0.098 | 1 |
|  | 2 | 0.63 | (95%-CI 0.33 to 1.19) | 0.153 | 1 |
|  | 3 | 0.46 | (95%-CI 0.23 to 0.96) | 0.037 | 0.519 |
| L-LDL-C | 1 | 0.68 | (95%-CI 0.45 to 1.02) | 0.063 | 0.887 |
|  | 2 | 0.64 | (95%-CI 0.3 to 1.35) | 0.24 | 1 |
|  | 3 | 0.31 | (95%-CI 0.13 to 0.72) | 0.006 | 0.087 |
| M-LDL-C | 1 | 0.87 | (95%-CI 0.56 to 1.34) | 0.521 | 1 |
|  | 2 | 0.64 | (95%-CI 0.32 to 1.28) | 0.204 | 1 |
|  | 3 | 0.6 | (95%-CI 0.3 to 1.2) | 0.152 | 1 |
| S-LDL-C | 1 | 0.69 | (95%-CI 0.46 to 1.04) | 0.075 | 1 |
|  | 2 | 0.89 | (95%-CI 0.5 to 1.59) | 0.699 | 1 |
|  | 3 | 0.66 | (95%-CI 0.37 to 1.16) | 0.15 | 1 |
| XL-HDL-C | 1 | 0.84 | (95%-CI 0.53 to 1.31) | 0.434 | 1 |
|  | 2 | 1.02 | (95%-CI 0.63 to 1.65) | 0.944 | 1 |
|  | 3 | 1.11 | (95%-CI 0.64 to 1.91) | 0.712 | 1 |
| L-HDL-C | 1 | 0.96 | (95%-CI 0.61 to 1.5) | 0.86 | 1 |
|  | 2 | 1.11 | (95%-CI 0.76 to 1.63) | 0.573 | 1 |
|  | 3 | 0.8 | (95%-CI 0.39 to 1.64) | 0.536 | 1 |
| M-HDL-C | 1 | 0.73 | (95%-CI 0.45 to 1.16) | 0.183 | 1 |
|  | 2 | 0.79 | (95%-CI 0.46 to 1.37) | 0.408 | 1 |
|  | 3 | 0.54 | (95%-CI 0.29 to 1.01) | 0.055 | 0.766 |
| S-HDL-C | 1 | 0.8 | (95%-CI 0.55 to 1.15) | 0.228 | 1 |
|  | 2 | 0.57 | (95%-CI 0.36 to 0.9) | 0.016 | 0.218 |
|  | 3 | 0.41 | (95%-CI 0.22 to 0.75) | 0.004 | 0.055 |
|  | | | |  |  |
|  | | | |  |  |
|  | | | |  |  |
|  | | | |  |  |
|  | | | |  |  |
|  | | | |  |  |
| **Cholesterol esters in lipoprotein particles** | | | |  |  |
| **Metabolite** | **Timepoint** | **HR** | **95%-CI** | **Unadjusted p-value** | **Adjusted p-value** |
| XXL-VLDL-CE | 1 | 0.72 | (95%-CI 0.49 to 1.07) | 0.103 | 1 |
|  | 2 | 0.83 | (95%-CI 0.57 to 1.2) | 0.326 | 1 |
|  | 3 | 0.96 | (95%-CI 0.55 to 1.7) | 0.899 | 1 |
| XL-VLDL-CE | 1 | 1.02 | (95%-CI 0.64 to 1.63) | 0.939 | 1 |
|  | 2 | 0.79 | (95%-CI 0.45 to 1.4) | 0.422 | 1 |
|  | 3 | 1.13 | (95%-CI 0.62 to 2.04) | 0.696 | 1 |
| L-VLDL-CE | 1 | 0.9 | (95%-CI 0.55 to 1.46) | 0.659 | 1 |
|  | 2 | 0.84 | (95%-CI 0.58 to 1.22) | 0.363 | 1 |
|  | 3 | 0.96 | (95%-CI 0.55 to 1.69) | 0.891 | 1 |
| M-VLDL-CE | 1 | 0.63 | (95%-CI 0.44 to 0.89) | 0.01 | 0.134 |
|  | 2 | 0.66 | (95%-CI 0.47 to 0.94) | 0.021 | 0.293 |
|  | 3 | 0.76 | (95%-CI 0.56 to 1.01) | 0.062 | 0.871 |
| S-VLDL-CE | 1 | 0.68 | (95%-CI 0.42 to 1.09) | 0.107 | 1 |
|  | 2 | 0.48 | (95%-CI 0.25 to 0.95) | 0.034 | 0.482 |
|  | 3 | 0.49 | (95%-CI 0.26 to 0.93) | 0.028 | 0.394 |
| XS-VLDL-CE | 1 | 0.72 | (95%-CI 0.49 to 1.06) | 0.099 | 1 |
|  | 2 | 0.62 | (95%-CI 0.35 to 1.11) | 0.11 | 1 |
|  | 3 | 0.6 | (95%-CI 0.35 to 1.03) | 0.062 | 0.872 |
| IDL-CE | 1 | 0.7 | (95%-CI 0.47 to 1.04) | 0.079 | 1 |
|  | 2 | 0.61 | (95%-CI 0.32 to 1.15) | 0.124 | 1 |
|  | 3 | 0.45 | (95%-CI 0.21 to 0.94) | 0.033 | 0.469 |
| L-LDL-CE | 1 | 0.67 | (95%-CI 0.45 to 1.01) | 0.057 | 0.802 |
|  | 2 | 0.6 | (95%-CI 0.28 to 1.3) | 0.195 | 1 |
|  | 3 | 0.29 | (95%-CI 0.12 to 0.69) | 0.005 | 0.076 |
| M-LDL-CE | 1 | 0.92 | (95%-CI 0.62 to 1.37) | 0.683 | 1 |
|  | 2 | 0.61 | (95%-CI 0.29 to 1.26) | 0.182 | 1 |
|  | 3 | 0.6 | (95%-CI 0.29 to 1.25) | 0.174 | 1 |
| S-LDL-CE | 1 | 0.66 | (95%-CI 0.47 to 0.94) | 0.019 | 0.269 |
|  | 2 | 1.02 | (95%-CI 0.63 to 1.66) | 0.921 | 1 |
|  | 3 | 0.75 | (95%-CI 0.45 to 1.23) | 0.253 | 1 |
| XL-HDL-CE | 1 | 0.84 | (95%-CI 0.54 to 1.32) | 0.46 | 1 |
|  | 2 | 0.99 | (95%-CI 0.6 to 1.65) | 0.977 | 1 |
|  | 3 | 1.21 | (95%-CI 0.69 to 2.13) | 0.501 | 1 |
| L-HDL-CE | 1 | 0.97 | (95%-CI 0.62 to 1.51) | 0.878 | 1 |
|  | 2 | 1.12 | (95%-CI 0.77 to 1.63) | 0.567 | 1 |
|  | 3 | 0.81 | (95%-CI 0.39 to 1.65) | 0.557 | 1 |
| M-HDL-CE | 1 | 0.7 | (95%-CI 0.44 to 1.14) | 0.152 | 1 |
|  | 2 | 0.77 | (95%-CI 0.44 to 1.34) | 0.356 | 1 |
|  | 3 | 0.56 | (95%-CI 0.3 to 1.02) | 0.057 | 0.796 |
| S-HDL-CE | 1 | 0.8 | (95%-CI 0.56 to 1.14) | 0.217 | 1 |
|  | 2 | 0.58 | (95%-CI 0.38 to 0.89) | 0.013 | 0.179 |
|  | 3 | 0.36 | (95%-CI 0.19 to 0.68) | 0.001 | 0.021* |
|  | | |  |  |  |
|  | | |  |  |  |
|  | | |  |  |  |
|  | | |  |  |  |
|  | | |  |  |  |
|  | | |  |  |  |
| **Free cholesterol in lipoprotein particles** | | |  |  |  |
| **Metabolite** | **Timepoint** | **HR** | **95%-CI** | **Unadjusted p-value** | **Adjusted p-value** |
| XXL-VLDL-FC | 1 | 0.64 | (95%-CI 0.44 to 0.93) | 0.019 | 0.26 |
|  | 2 | 0.79 | (95%-CI 0.54 to 1.15) | 0.219 | 1 |
|  | 3 | 1.01 | (95%-CI 0.58 to 1.75) | 0.984 | 1 |
| XL-VLDL-FC | 1 | 0.94 | (95%-CI 0.59 to 1.51) | 0.804 | 1 |
|  | 2 | 0.76 | (95%-CI 0.48 to 1.19) | 0.226 | 1 |
|  | 3 | 0.97 | (95%-CI 0.58 to 1.63) | 0.905 | 1 |
| L-VLDL-FC | 1 | 0.82 | (95%-CI 0.52 to 1.3) | 0.4 | 1 |
|  | 2 | 0.77 | (95%-CI 0.53 to 1.11) | 0.162 | 1 |
|  | 3 | 0.78 | (95%-CI 0.4 to 1.51) | 0.46 | 1 |
| M-VLDL-FC | 1 | 0.7 | (95%-CI 0.49 to 0.99) | 0.044 | 0.614 |
|  | 2 | 0.81 | (95%-CI 0.55 to 1.18) | 0.265 | 1 |
|  | 3 | 0.86 | (95%-CI 0.53 to 1.42) | 0.564 | 1 |
| S-VLDL-FC | 1 | 0.74 | (95%-CI 0.44 to 1.22) | 0.237 | 1 |
|  | 2 | 0.73 | (95%-CI 0.45 to 1.18) | 0.2 | 1 |
|  | 3 | 0.98 | (95%-CI 0.63 to 1.54) | 0.945 | 1 |
| XS-VLDL-FC | 1 | 0.82 | (95%-CI 0.55 to 1.2) | 0.304 | 1 |
|  | 2 | 0.69 | (95%-CI 0.39 to 1.23) | 0.205 | 1 |
|  | 3 | 0.61 | (95%-CI 0.36 to 1.04) | 0.071 | 0.998 |
| IDL-FC | 1 | 0.77 | (95%-CI 0.52 to 1.15) | 0.199 | 1 |
|  | 2 | 0.72 | (95%-CI 0.39 to 1.32) | 0.282 | 1 |
|  | 3 | 0.52 | (95%-CI 0.27 to 1.01) | 0.053 | 0.739 |
| L-LDL-FC | 1 | 0.7 | (95%-CI 0.46 to 1.06) | 0.095 | 1 |
|  | 2 | 0.77 | (95%-CI 0.38 to 1.53) | 0.449 | 1 |
|  | 3 | 0.39 | (95%-CI 0.19 to 0.81) | 0.011 | 0.153 |
| M-LDL-FC | 1 | 0.82 | (95%-CI 0.52 to 1.28) | 0.385 | 1 |
|  | 2 | 0.7 | (95%-CI 0.32 to 1.5) | 0.358 | 1 |
|  | 3 | 0.62 | (95%-CI 0.3 to 1.3) | 0.209 | 1 |
| S-LDL-FC | 1 | 0.65 | (95%-CI 0.41 to 1.05) | 0.077 | 1 |
|  | 2 | 0.93 | (95%-CI 0.37 to 2.31) | 0.874 | 1 |
|  | 3 | 0.6 | (95%-CI 0.24 to 1.51) | 0.28 | 1 |
| XL-HDL-FC | 1 | 0.83 | (95%-CI 0.54 to 1.3) | 0.422 | 1 |
|  | 2 | 1.06 | (95%-CI 0.7 to 1.63) | 0.773 | 1 |
|  | 3 | 0.91 | (95%-CI 0.56 to 1.5) | 0.72 | 1 |
| L-HDL-FC | 1 | 0.94 | (95%-CI 0.6 to 1.48) | 0.802 | 1 |
|  | 2 | 1.11 | (95%-CI 0.76 to 1.62) | 0.593 | 1 |
|  | 3 | 0.77 | (95%-CI 0.37 to 1.59) | 0.471 | 1 |
| M-HDL-FC | 1 | 0.81 | (95%-CI 0.52 to 1.26) | 0.354 | 1 |
|  | 2 | 0.89 | (95%-CI 0.53 to 1.48) | 0.646 | 1 |
|  | 3 | 0.48 | (95%-CI 0.23 to 1) | 0.05 | 0.697 |
| S-HDL-FC | 1 | 0.94 | (95%-CI 0.6 to 1.49) | 0.807 | 1 |
|  | 2 | 0.87 | (95%-CI 0.54 to 1.39) | 0.563 | 1 |
|  | 3 | 0.85 | (95%-CI 0.52 to 1.38) | 0.513 | 1 |
|  | | |  |  |  |
|  | | |  |  |  |
|  | | |  |  |  |
|  | | |  |  |  |
|  | | |  |  |  |
|  | | |  |  |  |
| **Triglycerides in lipoprotein particles** | | |  |  |  |
| **Metabolite** | **Timepoint** | **HR** | **95%-CI** | **Unadjusted p-value** | **Adjusted p-value** |
| XXL-VLDL-TG | 1 | 0.57 | (95%-CI 0.34 to 0.96) | 0.034 | 0.472 |
|  | 2 | 0.82 | (95%-CI 0.55 to 1.23) | 0.334 | 1 |
|  | 3 | 1.21 | (95%-CI 0.75 to 1.95) | 0.442 | 1 |
| XL-VLDL-TG | 1 | 0.87 | (95%-CI 0.51 to 1.48) | 0.611 | 1 |
|  | 2 | 0.77 | (95%-CI 0.42 to 1.43) | 0.414 | 1 |
|  | 3 | 1.17 | (95%-CI 0.67 to 2.03) | 0.584 | 1 |
| L-VLDL-TG | 1 | 0.87 | (95%-CI 0.52 to 1.44) | 0.577 | 1 |
|  | 2 | 0.8 | (95%-CI 0.53 to 1.21) | 0.295 | 1 |
|  | 3 | 0.94 | (95%-CI 0.54 to 1.64) | 0.826 | 1 |
| M-VLDL-TG | 1 | 0.62 | (95%-CI 0.4 to 0.97) | 0.035 | 0.488 |
|  | 2 | 0.83 | (95%-CI 0.55 to 1.26) | 0.387 | 1 |
|  | 3 | 0.94 | (95%-CI 0.57 to 1.55) | 0.8 | 1 |
| S-VLDL-TG | 1 | 0.66 | (95%-CI 0.4 to 1.1) | 0.114 | 1 |
|  | 2 | 0.77 | (95%-CI 0.5 to 1.19) | 0.235 | 1 |
|  | 3 | 1.05 | (95%-CI 0.64 to 1.7) | 0.853 | 1 |
| XS-VLDL-TG | 1 | 1.03 | (95%-CI 0.7 to 1.52) | 0.866 | 1 |
|  | 2 | 0.81 | (95%-CI 0.53 to 1.23) | 0.319 | 1 |
|  | 3 | 1.13 | (95%-CI 0.72 to 1.79) | 0.592 | 1 |
| IDL-TG | 1 | 1.22 | (95%-CI 0.81 to 1.83) | 0.349 | 1 |
|  | 2 | 1.06 | (95%-CI 0.64 to 1.74) | 0.831 | 1 |
|  | 3 | 1.39 | (95%-CI 0.87 to 2.21) | 0.164 | 1 |
| L-LDL-TG | 1 | 1.08 | (95%-CI 0.72 to 1.62) | 0.704 | 1 |
|  | 2 | 1.21 | (95%-CI 0.73 to 2.02) | 0.457 | 1 |
|  | 3 | 1.77 | (95%-CI 1.05 to 2.97) | 0.032 | 0.442 |
| M-LDL-TG | 1 | 1.34 | (95%-CI 0.79 to 2.26) | 0.278 | 1 |
|  | 2 | 1.25 | (95%-CI 0.58 to 2.69) | 0.568 | 1 |
|  | 3 | 1.84 | (95%-CI 0.85 to 3.98) | 0.123 | 1 |
| S-LDL-TG | 1 | 0.85 | (95%-CI 0.54 to 1.33) | 0.469 | 1 |
|  | 2 | 1.11 | (95%-CI 0.58 to 2.11) | 0.757 | 1 |
|  | 3 | 1.54 | (95%-CI 0.76 to 3.14) | 0.231 | 1 |
| XL-HDL-TG | 1 | 0.93 | (95%-CI 0.64 to 1.34) | 0.692 | 1 |
|  | 2 | 0.98 | (95%-CI 0.71 to 1.35) | 0.913 | 1 |
|  | 3 | 0.93 | (95%-CI 0.65 to 1.33) | 0.675 | 1 |
| L-HDL-TG | 1 | 1.27 | (95%-CI 0.81 to 1.99) | 0.301 | 1 |
|  | 2 | 1.18 | (95%-CI 0.81 to 1.72) | 0.382 | 1 |
|  | 3 | 1 | (95%-CI 0.65 to 1.53) | 0.991 | 1 |
| M-HDL-TG | 1 | 0.91 | (95%-CI 0.6 to 1.36) | 0.631 | 1 |
|  | 2 | 0.73 | (95%-CI 0.47 to 1.13) | 0.159 | 1 |
|  | 3 | 0.78 | (95%-CI 0.45 to 1.34) | 0.366 | 1 |
| S-HDL-TG | 1 | 1.34 | (95%-CI 0.91 to 1.97) | 0.136 | 1 |
|  | 2 | 1 | (95%-CI 0.7 to 1.43) | 0.983 | 1 |
|  | 3 | 1.49 | (95%-CI 0.98 to 2.25) | 0.06 | 0.837 |
|  | | |  |  |  |
|  | | |  |  |  |
|  | | |  |  |  |
|  | | |  |  |  |
|  | | |  |  |  |
|  | | |  |  |  |
| **Diameter of lipoprotein particles** | | |  |  |  |
| **Metabolite** | **Timepoint** | **HR** | **95%-CI** | **Unadjusted p-value** | **Adjusted p-value** |
| VLDL-D | 1 | 0.89 | (95%-CI 0.61 to 1.31) | 0.57 | 1 |
|  | 2 | 0.83 | (95%-CI 0.57 to 1.22) | 0.351 | 1 |
|  | 3 | 1.07 | (95%-CI 0.66 to 1.74) | 0.783 | 1 |
| LDL-D | 1 | 1.59 | (95%-CI 1.01 to 2.5) | 0.047 | 0.655 |
|  | 2 | 1.16 | (95%-CI 0.9 to 1.5) | 0.242 | 1 |
|  | 3 | 1.78 | (95%-CI 1.19 to 2.67) | 0.005 | 0.069 |
| HDL-D | 1 | 0.91 | (95%-CI 0.62 to 1.33) | 0.615 | 1 |
|  | 2 | 1.14 | (95%-CI 0.8 to 1.62) | 0.48 | 1 |
|  | 3 | 0.91 | (95%-CI 0.61 to 1.35) | 0.629 | 1 |
| **Cholesterol** |  |  |  |  |  |
| Serum-C | 1 | 0.67 | (95%-CI 0.45 to 1.01) | 0.054 | 0.75 |
|  | 2 | 0.52 | (95%-CI 0.3 to 0.92) | 0.024 | 0.342 |
|  | 3 | 0.44 | (95%-CI 0.23 to 0.81) | 0.009 | 0.127 |
| VLDL-C | 1 | 0.9 | (95%-CI 0.66 to 1.23) | 0.506 | 1 |
|  | 2 | 0.63 | (95%-CI 0.46 to 0.88) | 0.006 | 0.085 |
|  | 3 | 0.8 | (95%-CI 0.62 to 1.02) | 0.076 | 1 |
| Remnant-C | 1 | 0.71 | (95%-CI 0.48 to 1.05) | 0.086 | 1 |
|  | 2 | 0.5 | (95%-CI 0.29 to 0.87) | 0.014 | 0.19 |
|  | 3 | 0.55 | (95%-CI 0.32 to 0.96) | 0.036 | 0.501 |
| LDL-C | 1 | 0.73 | (95%-CI 0.49 to 1.09) | 0.122 | 1 |
|  | 2 | 0.51 | (95%-CI 0.25 to 1.01) | 0.053 | 0.742 |
|  | 3 | 0.34 | (95%-CI 0.16 to 0.73) | 0.006 | 0.078 |
| HDL-C | 1 | 0.79 | (95%-CI 0.53 to 1.18) | 0.257 | 1 |
|  | 2 | 0.91 | (95%-CI 0.59 to 1.4) | 0.67 | 1 |
|  | 3 | 0.61 | (95%-CI 0.34 to 1.1) | 0.1 | 1 |
| HDL2-C | 1 | 0.82 | (95%-CI 0.54 to 1.22) | 0.326 | 1 |
|  | 2 | 0.96 | (95%-CI 0.62 to 1.47) | 0.844 | 1 |
|  | 3 | 0.63 | (95%-CI 0.36 to 1.12) | 0.118 | 1 |
| HDL3-C | 1 | 0.87 | (95%-CI 0.6 to 1.28) | 0.487 | 1 |
|  | 2 | 0.88 | (95%-CI 0.59 to 1.31) | 0.517 | 1 |
|  | 3 | 0.77 | (95%-CI 0.46 to 1.31) | 0.34 | 1 |
| EstC | 1 | 0.7 | (95%-CI 0.43 to 1.14) | 0.149 | 1 |
|  | 2 | 0.62 | (95%-CI 0.35 to 1.11) | 0.11 | 1 |
|  | 3 | 0.57 | (95%-CI 0.3 to 1.08) | 0.087 | 1 |
| FreeC | 1 | 0.71 | (95%-CI 0.42 to 1.19) | 0.196 | 1 |
|  | 2 | 0.95 | (95%-CI 0.53 to 1.69) | 0.856 | 1 |
|  | 3 | 0.56 | (95%-CI 0.26 to 1.2) | 0.137 | 1 |
|  | | |  |  |  |
|  | | |  |  |  |
|  | | |  |  |  |
|  | | |  |  |  |
|  | | |  |  |  |
|  | | |  |  |  |
|  | | |  |  |  |
|  | | |  |  |  |
|  | | |  |  |  |
|  | | |  |  |  |
|  | | |  |  |  |
| **Glycerides and phospholipids** | | |  |  |  |
| **Metabolite** | **Timepoint** | **HR** | **95%-CI** | **Unadjusted p-value** | **Adjusted p-value** |
| Serum-TG | 1 | 0.88 | (95%-CI 0.6 to 1.29) | 0.506 | 1 |
|  | 2 | 0.74 | (95%-CI 0.5 to 1.09) | 0.126 | 1 |
|  | 3 | 1 | (95%-CI 0.64 to 1.56) | 0.99 | 1 |
| VLDL-TG | 1 | 0.86 | (95%-CI 0.59 to 1.24) | 0.412 | 1 |
|  | 2 | 0.73 | (95%-CI 0.49 to 1.08) | 0.114 | 1 |
|  | 3 | 0.96 | (95%-CI 0.59 to 1.56) | 0.882 | 1 |
| LDL-TG | 1 | 1.13 | (95%-CI 0.76 to 1.68) | 0.548 | 1 |
|  | 2 | 0.84 | (95%-CI 0.59 to 1.22) | 0.365 | 1 |
|  | 3 | 1.19 | (95%-CI 0.78 to 1.81) | 0.419 | 1 |
| HDL-TG | 1 | 0.99 | (95%-CI 0.67 to 1.47) | 0.973 | 1 |
|  | 2 | 0.84 | (95%-CI 0.52 to 1.35) | 0.468 | 1 |
|  | 3 | 1.11 | (95%-CI 0.69 to 1.78) | 0.674 | 1 |
| TotPG | 1 | 0.62 | (95%-CI 0.35 to 1.1) | 0.103 | 1 |
|  | 2 | 1.27 | (95%-CI 0.82 to 1.97) | 0.284 | 1 |
|  | 3 | 0.91 | (95%-CI 0.51 to 1.65) | 0.759 | 1 |
| TG-PG | 1 | 0.65 | (95%-CI 0.38 to 1.09) | 0.102 | 1 |
|  | 2 | 0.92 | (95%-CI 0.68 to 1.25) | 0.596 | 1 |
|  | 3 | 1.11 | (95%-CI 0.59 to 2.09) | 0.756 | 1 |
| PC | 1 | 0.56 | (95%-CI 0.32 to 1) | 0.052 | 0.721 |
|  | 2 | 1.36 | (95%-CI 0.81 to 2.31) | 0.247 | 1 |
|  | 3 | 0.86 | (95%-CI 0.48 to 1.56) | 0.63 | 1 |
| SM | 1 | 0.81 | (95%-CI 0.5 to 1.33) | 0.411 | 1 |
|  | 2 | 1.2 | (95%-CI 0.87 to 1.66) | 0.259 | 1 |
|  | 3 | 0.96 | (95%-CI 0.68 to 1.36) | 0.838 | 1 |
| TotCho | 1 | 0.6 | (95%-CI 0.35 to 1.02) | 0.061 | 0.847 |
|  | 2 | 1.38 | (95%-CI 0.88 to 2.15) | 0.156 | 1 |
|  | 3 | 0.96 | (95%-CI 0.6 to 1.54) | 0.862 | 1 |
| **Apolipoproteins** | |  |  |  |  |
| **Metabolite** | **Timepoint** | **HR** | **95%-CI** | **Unadjusted p-value** | **Adjusted p-value** |
| ApoA1 | 1 | 0.63 | (95%-CI 0.41 to 0.97) | 0.035 | 0.493 |
|  | 2 | 0.68 | (95%-CI 0.43 to 1.08) | 0.098 | 1 |
|  | 3 | 0.56 | (95%-CI 0.34 to 0.92) | 0.022 | 0.311 |
| ApoB | 1 | 0.71 | (95%-CI 0.48 to 1.05) | 0.083 | 1 |
|  | 2 | 0.52 | (95%-CI 0.31 to 0.86) | 0.012 | 0.169 |
|  | 3 | 0.57 | (95%-CI 0.33 to 0.98) | 0.044 | 0.612 |
| ApoB-ApoA1 | 1 | 0.86 | (95%-CI 0.59 to 1.25) | 0.419 | 1 |
|  | 2 | 0.66 | (95%-CI 0.43 to 1.01) | 0.056 | 0.779 |
|  | 3 | 0.83 | (95%-CI 0.52 to 1.31) | 0.421 | 1 |
|  |  |  |  |  |  |
|  |  |  |  |  |  |
|  |  |  |  |  |  |
|  |  |  |  |  |  |
|  |  |  |  |  |  |
|  |  |  |  |  |  |
|  |  |  |  |  |  |
|  |  |  |  |  |  |
|  |  |  |  |  |  |
|  |  |  |  |  |  |
| **Fatty acids** |  |  |  |  |  |
| **Metabolite** | **Timepoint** | **HR** | **95%-CI** | **Unadjusted p-value** | **Adjusted p-value** |
| TotFA | 1 | 0.72 | (95%-CI 0.44 to 1.18) | 0.19 | 1 |
|  | 2 | 0.76 | (95%-CI 0.51 to 1.12) | 0.169 | 1 |
|  | 3 | 1.18 | (95%-CI 0.69 to 2.02) | 0.537 | 1 |
| UnSat | 1 | 0.84 | (95%-CI 0.56 to 1.26) | 0.396 | 1 |
|  | 2 | 1.1 | (95%-CI 0.77 to 1.57) | 0.61 | 1 |
|  | 3 | 0.34 | (95%-CI 0.13 to 0.9) | 0.03 | 0.424 |
| DHA | 1 | 0.93 | (95%-CI 0.55 to 1.57) | 0.79 | 1 |
|  | 2 | 1.05 | (95%-CI 0.69 to 1.6) | 0.823 | 1 |
|  | 3 | 0.77 | (95%-CI 0.41 to 1.46) | 0.429 | 1 |
| LA | 1 | 0.64 | (95%-CI 0.38 to 1.06) | 0.081 | 1 |
|  | 2 | 0.48 | (95%-CI 0.27 to 0.85) | 0.012 | 0.165 |
|  | 3 | 1.13 | (95%-CI 0.58 to 2.22) | 0.716 | 1 |
| FAw3 | 1 | 0.98 | (95%-CI 0.59 to 1.61) | 0.924 | 1 |
|  | 2 | 0.98 | (95%-CI 0.63 to 1.53) | 0.925 | 1 |
|  | 3 | 0.77 | (95%-CI 0.34 to 1.73) | 0.529 | 1 |
| FAw6 | 1 | 0.61 | (95%-CI 0.36 to 1.03) | 0.067 | 0.936 |
|  | 2 | 0.51 | (95%-CI 0.31 to 0.86) | 0.012 | 0.17 |
|  | 3 | 0.99 | (95%-CI 0.51 to 1.9) | 0.973 | 1 |
| PUFA | 1 | 0.64 | (95%-CI 0.38 to 1.08) | 0.095 | 1 |
|  | 2 | 0.59 | (95%-CI 0.36 to 0.97) | 0.037 | 0.522 |
|  | 3 | 0.95 | (95%-CI 0.5 to 1.81) | 0.878 | 1 |
| MUFA | 1 | 0.78 | (95%-CI 0.47 to 1.29) | 0.332 | 1 |
|  | 2 | 0.79 | (95%-CI 0.54 to 1.15) | 0.212 | 1 |
|  | 3 | 1.29 | (95%-CI 0.79 to 2.11) | 0.316 | 1 |
| SFA | 1 | 0.79 | (95%-CI 0.48 to 1.3) | 0.355 | 1 |
|  | 2 | 0.84 | (95%-CI 0.55 to 1.29) | 0.418 | 1 |
|  | 3 | 1.17 | (95%-CI 0.66 to 2.08) | 0.587 | 1 |
| DHA-FA | 1 | 1.1 | (95%-CI 0.67 to 1.8) | 0.701 | 1 |
|  | 2 | 1.12 | (95%-CI 0.73 to 1.7) | 0.608 | 1 |
|  | 3 | 0.68 | (95%-CI 0.39 to 1.18) | 0.171 | 1 |
| LA-FA | 1 | 0.67 | (95%-CI 0.41 to 1.09) | 0.106 | 1 |
|  | 2 | 0.62 | (95%-CI 0.39 to 0.99) | 0.045 | 0.631 |
|  | 3 | 1.11 | (95%-CI 0.55 to 2.24) | 0.779 | 1 |
| FAw3-FA | 1 | 1.04 | (95%-CI 0.66 to 1.65) | 0.858 | 1 |
|  | 2 | 1.56 | (95%-CI 0.9 to 2.71) | 0.114 | 1 |
|  | 3 | 0.51 | (95%-CI 0.18 to 1.43) | 0.199 | 1 |
| FAw6-FA | 1 | 0.77 | (95%-CI 0.49 to 1.19) | 0.238 | 1 |
|  | 2 | 0.73 | (95%-CI 0.48 to 1.11) | 0.136 | 1 |
|  | 3 | 0.8 | (95%-CI 0.42 to 1.52) | 0.486 | 1 |
| PUFA-FA | 1 | 0.79 | (95%-CI 0.52 to 1.21) | 0.284 | 1 |
|  | 2 | 0.81 | (95%-CI 0.54 to 1.21) | 0.303 | 1 |
|  | 3 | 0.7 | (95%-CI 0.36 to 1.37) | 0.298 | 1 |
| MUFA-FA | 1 | 1.16 | (95%-CI 0.73 to 1.86) | 0.534 | 1 |
|  | 2 | 0.93 | (95%-CI 0.65 to 1.33) | 0.678 | 1 |
|  | 3 | 1.48 | (95%-CI 0.84 to 2.6) | 0.175 | 1 |
| SFA-FA | 1 | 1.35 | (95%-CI 0.88 to 2.07) | 0.171 | 1 |
|  | 2 | 1.48 | (95%-CI 1.09 to 2.01) | 0.012 | 0.171 |
|  | 3 | 0.95 | (95%-CI 0.65 to 1.4) | 0.803 | 1 |
| **Glycolysis related** | |  |  |  |  |
| **Metabolite** | **Timepoint** | **HR** | **95%-CI** | **Unadjusted p-value** | **Adjusted p-value** |
| Glc | 1 | 1.62 | (95%-CI 1.1 to 2.39) | 0.016 | 0.218 |
|  | 2 | 1.18 | (95%-CI 0.67 to 2.07) | 0.574 | 1 |
|  | 3 | 1.61 | (95%-CI 0.95 to 2.75) | 0.079 | 1 |
| Lac | 1 | 1.71 | (95%-CI 1.16 to 2.53) | 0.007 | 0.096 |
|  | 2 | 2.25 | (95%-CI 1.53 to 3.3) | <0.001 | <0.001* |
|  | 3 | 2.77 | (95%-CI 1.76 to 4.36) | <0.001 | <0.001* |
| Cit | 1 | 0.91 | (95%-CI 0.59 to 1.43) | 0.691 | 1 |
|  | 2 | 1.28 | (95%-CI 1.06 to 1.54) | 0.01 | 0.144 |
|  | 3 | 1.04 | (95%-CI 0.95 to 1.14) | 0.395 | 1 |
| **Amino acids** |  |  |  |  |  |
| **Metabolite** | **Timepoint** | **HR** | **95%-CI** | **Unadjusted p-value** | **Adjusted p-value** |
| Ala | 1 | 1.33 | (95%-CI 0.93 to 1.89) | 0.118 | 1 |
|  | 2 | 1.13 | (95%-CI 0.79 to 1.6) | 0.511 | 1 |
|  | 3 | 2.43 | (95%-CI 1.56 to 3.78) | <0.001 | 0.001* |
| Gln | 1 | 0.88 | (95%-CI 0.56 to 1.4) | 0.598 | 1 |
|  | 2 | 0.83 | (95%-CI 0.57 to 1.2) | 0.318 | 1 |
|  | 3 | 0.97 | (95%-CI 0.54 to 1.73) | 0.907 | 1 |
| His | 1 | 1.11 | (95%-CI 0.71 to 1.72) | 0.647 | 1 |
|  | 2 | 1.1 | (95%-CI 0.74 to 1.64) | 0.638 | 1 |
|  | 3 | 1.44 | (95%-CI 0.84 to 2.47) | 0.189 | 1 |
| Ile | 1 | 0.9 | (95%-CI 0.59 to 1.38) | 0.641 | 1 |
|  | 2 | 0.84 | (95%-CI 0.74 to 0.96) | 0.01 | 0.138 |
|  | 3 | 1 | (95%-CI 0.65 to 1.52) | 0.989 | 1 |
| Leu | 1 | 0.97 | (95%-CI 0.63 to 1.52) | 0.91 | 1 |
|  | 2 | 0.64 | (95%-CI 0.5 to 0.82) | 0.001 | 0.007* |
|  | 3 | 1.15 | (95%-CI 0.83 to 1.59) | 0.401 | 1 |
| Val | 1 | 1.16 | (95%-CI 0.74 to 1.83) | 0.517 | 1 |
|  | 2 | 0.37 | (95%-CI 0.22 to 0.63) | 0 | 0.003* |
|  | 3 | 1.03 | (95%-CI 0.63 to 1.7) | 0.899 | 1 |
| Phe | 1 | 1.1 | (95%-CI 0.72 to 1.68) | 0.669 | 1 |
|  | 2 | 0.83 | (95%-CI 0.58 to 1.21) | 0.337 | 1 |
|  | 3 | 1.73 | (95%-CI 1.05 to 2.87) | 0.033 | 0.457 |
| Tyr | 1 | 1.02 | (95%-CI 0.69 to 1.52) | 0.913 | 1 |
|  | 2 | 0.82 | (95%-CI 0.61 to 1.11) | 0.207 | 1 |
|  | 3 | 1.38 | (95%-CI 0.96 to 2) | 0.085 | 1 |
| **Ketone bodies** | |  |  |  |  |
| **Metabolite** | **Timepoint** | **HR** | **95%-CI** | **Unadjusted p-value** | **Adjusted p-value** |
| Ace | 1 | 1.09 | (95%-CI 0.76 to 1.58) | 0.628 | 1 |
|  | 2 | 0.91 | (95%-CI 0.48 to 1.73) | 0.781 | 1 |
|  | 3 | 1.09 | (95%-CI 0.6 to 1.98) | 0.776 | 1 |
| AcAce | 1 | 1.03 | (95%-CI 0.71 to 1.5) | 0.874 | 1 |
|  | 2 | 0.8 | (95%-CI 0.58 to 1.1) | 0.165 | 1 |
|  | 3 | 0.88 | (95%-CI 0.77 to 1) | 0.049 | 0.687 |
| bOHBut | 1 | 1.2 | (95%-CI 0.82 to 1.75) | 0.339 | 1 |
|  | 2 | 0.57 | (95%-CI 0.35 to 0.91) | 0.019 | 0.263 |
|  | 3 | 0.85 | (95%-CI 0.47 to 1.52) | 0.573 | 1 |
|  | |  |  |  |  |
|  | |  |  |  |  |
| **Albumin and creatinine** | |  |  |  |  |
| **Metabolite** | **Timepoint** | **HR** | **95%-CI** | **Unadjusted p-value** | **Adjusted p-value** |
| Crea | 1 | 1.9 | (95%-CI 0.89 to 4.08) | 0.098 | 1 |
|  | 2 | 1.73 | (95%-CI 0.92 to 3.28) | 0.09 | 1 |
|  | 3 | 2.18 | (95%-CI 0.96 to 4.94) | 0.063 | 0.885 |
| Alb | 1 | 0.74 | (95%-CI 0.47 to 1.16) | 0.19 | 1 |
|  | 2 | 0.81 | (95%-CI 0.51 to 1.28) | 0.364 | 1 |
|  | 3 | 0.63 | (95%-CI 0.38 to 1.06) | 0.084 | 1 |
| **Inflammation** | |  |  |  |  |
| **Metabolite** | **Timepoint** | **HR** | **95%-CI** | **Unadjusted p-value** | **Adjusted p-value** |
| Gp | 1 | 0.83 | (95%-CI 0.55 to 1.26) | 0.389 | 1 |
|  | 2 | 0.83 | (95%-CI 0.59 to 1.16) | 0.271 | 1 |
|  | 3 | 0.82 | (95%-CI 0.55 to 1.21) | 0.316 | 1 |
