# Supplementary material for: A metabolic profile of xenon and metabolite associations with 6-month mortality after out-of-hospital cardiac arrest: A post-hoc study of the randomised Xe-Hypotheca trial
Source: PLoS One. 2024 Jun 4;19(6):e0304966. doi: 10.1371/journal.pone.0304966 (PMC11149864; doi:10.1371/journal.pone.0304966)
Supplement: S3 Table — (DOCX) [file pone.0304966.s006.docx]

**S3 Table** All metabolite differences in timepoint comparisons, xenon *vs.* control. Data is reported as adjusted mean difference with 95 % confidence interval (95%-CI), unadjusted and adjusted p-value. Based on principal component analysis over 95% of variation in metabolomic data was explained by 14 components in all three time points, thus the p-values have been adjusted by a factor of 14 for multiple testing, an alpha threshold of 0.05 was used.

| **Concentration of lipoprotein particles** | | |  |  |  |
| --- | --- | --- | --- | --- | --- |
| **Metabolite** | **Timepoint** | **Adjusted mean difference of metabolite change from baseline** | **95%-CI** | **Unadjusted p-value** | **Adjusted p-value** |
| XXL-VLDL-P | 1 | -0.13 | (95%-CI -0.53 to 0.27) | 0.527 | 1 |
|  | 2 | -0.25 | (95%-CI -0.6 to 0.1) | 0.166 | 1 |
|  | 3 | -0.2 | (95%-CI -0.59 to 0.19) | 0.311 | 1 |
| XL-VLDL-P | 1 | -0.02 | (95%-CI -0.41 to 0.38) | 0.939 | 1 |
|  | 2 | -0.35 | (95%-CI -0.72 to 0.03) | 0.072 | 1 |
|  | 3 | -0.23 | (95%-CI -0.63 to 0.18) | 0.267 | 1 |
| L-VLDL-P | 1 | -0.02 | (95%-CI -0.41 to 0.38) | 0.938 | 1 |
|  | 2 | -0.19 | (95%-CI -0.55 to 0.16) | 0.286 | 1 |
|  | 3 | -0.09 | (95%-CI -0.49 to 0.31) | 0.669 | 1 |
| M-VLDL-P | 1 | -0.26 | (95%-CI -0.62 to 0.1) | 0.151 | 1 |
|  | 2 | -0.2 | (95%-CI -0.49 to 0.08) | 0.162 | 1 |
|  | 3 | 0.13 | (95%-CI -0.18 to 0.44) | 0.411 | 1 |
| S-VLDL-P | 1 | -0.26 | (95%-CI -0.65 to 0.13) | 0.195 | 1 |
|  | 2 | -0.2 | (95%-CI -0.52 to 0.12) | 0.213 | 1 |
|  | 3 | 0.05 | (95%-CI -0.29 to 0.4) | 0.76 | 1 |
| XS-VLDL-P | 1 | -0.39 | (95%-CI -0.74 to -0.03) | 0.031 | 0.44 |
|  | 2 | 0.07 | (95%-CI -0.28 to 0.41) | 0.707 | 1 |
|  | 3 | 0.21 | (95%-CI -0.16 to 0.58) | 0.256 | 1 |
| IDL-P | 1 | -0.26 | (95%-CI -0.6 to 0.07) | 0.124 | 1 |
|  | 2 | 0.16 | (95%-CI -0.14 to 0.46) | 0.3 | 1 |
|  | 3 | 0.11 | (95%-CI -0.22 to 0.44) | 0.524 | 1 |
| L-LDL-P | 1 | -0.22 | (95%-CI -0.55 to 0.12) | 0.206 | 1 |
|  | 2 | 0.14 | (95%-CI -0.2 to 0.48) | 0.415 | 1 |
|  | 3 | 0.27 | (95%-CI -0.1 to 0.65) | 0.154 | 1 |
| M-LDL-P | 1 | -0.31 | (95%-CI -0.7 to 0.07) | 0.11 | 1 |
|  | 2 | 0.02 | (95%-CI -0.41 to 0.45) | 0.937 | 1 |
|  | 3 | 0.34 | (95%-CI -0.11 to 0.8) | 0.137 | 1 |
| S-LDL-P | 1 | -0.22 | (95%-CI -0.56 to 0.13) | 0.221 | 1 |
|  | 2 | 0.03 | (95%-CI -0.34 to 0.4) | 0.874 | 1 |
|  | 3 | 0.18 | (95%-CI -0.21 to 0.57) | 0.357 | 1 |
| XL-HDL-P | 1 | 0.16 | (95%-CI -0.18 to 0.49) | 0.362 | 1 |
|  | 2 | -0.18 | (95%-CI -0.45 to 0.09) | 0.185 | 1 |
|  | 3 | -0.23 | (95%-CI -0.52 to 0.07) | 0.13 | 1 |
| L-HDL-P | 1 | 0.33 | (95%-CI -0.01 to 0.68) | 0.06 | 0.836 |
|  | 2 | -0.24 | (95%-CI -0.56 to 0.08) | 0.138 | 1 |
|  | 3 | -0.28 | (95%-CI -0.65 to 0.08) | 0.13 | 1 |
| M-HDL-P | 1 | 0.23 | (95%-CI -0.07 to 0.54) | 0.127 | 1 |
|  | 2 | -0.2 | (95%-CI -0.5 to 0.1) | 0.193 | 1 |
|  | 3 | -0.12 | (95%-CI -0.48 to 0.24) | 0.516 | 1 |
| S-HDL-P | 1 | 0.21 | (95%-CI -0.11 to 0.53) | 0.198 | 1 |
|  | 2 | -0.15 | (95%-CI -0.48 to 0.18) | 0.376 | 1 |
|  | 3 | -0.02 | (95%-CI -0.38 to 0.34) | 0.919 | 1 |
|  | | |  |  |  |
|  | | |  |  |  |
|  | | |  |  |  |
| **Total lipids in lipoprotein particles** | | |  |  |  |
| **Metabolite** | **Timepoint** | **Adjusted mean difference of metabolite change from baseline** | **95%-CI** | **Unadjusted p-value** | **Adjusted p-value** |
| XXL-VLDL-L | 1 | -0.13 | (95%-CI -0.53 to 0.26) | 0.506 | 1 |
|  | 2 | -0.25 | (95%-CI -0.6 to 0.11) | 0.172 | 1 |
|  | 3 | -0.19 | (95%-CI -0.58 to 0.2) | 0.339 | 1 |
| XL-VLDL-L | 1 | -0.03 | (95%-CI -0.43 to 0.37) | 0.89 | 1 |
|  | 2 | -0.35 | (95%-CI -0.73 to 0.03) | 0.074 | 1 |
|  | 3 | -0.23 | (95%-CI -0.63 to 0.18) | 0.277 | 1 |
| L-VLDL-L | 1 | -0.02 | (95%-CI -0.42 to 0.37) | 0.913 | 1 |
|  | 2 | -0.19 | (95%-CI -0.55 to 0.17) | 0.291 | 1 |
|  | 3 | -0.08 | (95%-CI -0.48 to 0.32) | 0.685 | 1 |
| M-VLDL-L | 1 | -0.27 | (95%-CI -0.62 to 0.09) | 0.146 | 1 |
|  | 2 | -0.21 | (95%-CI -0.49 to 0.08) | 0.152 | 1 |
|  | 3 | 0.13 | (95%-CI -0.18 to 0.45) | 0.406 | 1 |
| S-VLDL-L | 1 | -0.26 | (95%-CI -0.66 to 0.13) | 0.19 | 1 |
|  | 2 | -0.2 | (95%-CI -0.52 to 0.12) | 0.226 | 1 |
|  | 3 | 0.07 | (95%-CI -0.28 to 0.43) | 0.68 | 1 |
| XS-VLDL-L | 1 | -0.39 | (95%-CI -0.74 to -0.04) | 0.028 | 0.39 |
|  | 2 | 0.1 | (95%-CI -0.25 to 0.44) | 0.584 | 1 |
|  | 3 | 0.23 | (95%-CI -0.14 to 0.6) | 0.217 | 1 |
| IDL-L | 1 | -0.26 | (95%-CI -0.6 to 0.07) | 0.123 | 1 |
|  | 2 | 0.17 | (95%-CI -0.13 to 0.47) | 0.262 | 1 |
|  | 3 | 0.11 | (95%-CI -0.21 to 0.44) | 0.5 | 1 |
| L-LDL-L | 1 | -0.22 | (95%-CI -0.55 to 0.12) | 0.202 | 1 |
|  | 2 | 0.16 | (95%-CI -0.19 to 0.5) | 0.367 | 1 |
|  | 3 | 0.28 | (95%-CI -0.1 to 0.65) | 0.144 | 1 |
| M-LDL-L | 1 | -0.32 | (95%-CI -0.7 to 0.07) | 0.105 | 1 |
|  | 2 | 0.04 | (95%-CI -0.39 to 0.47) | 0.859 | 1 |
|  | 3 | 0.36 | (95%-CI -0.1 to 0.82) | 0.124 | 1 |
| S-LDL-L | 1 | -0.22 | (95%-CI -0.57 to 0.13) | 0.211 | 1 |
|  | 2 | 0.05 | (95%-CI -0.32 to 0.42) | 0.803 | 1 |
|  | 3 | 0.2 | (95%-CI -0.19 to 0.59) | 0.32 | 1 |
| XL-HDL-L | 1 | 0.15 | (95%-CI -0.19 to 0.49) | 0.378 | 1 |
|  | 2 | -0.17 | (95%-CI -0.44 to 0.1) | 0.209 | 1 |
|  | 3 | -0.22 | (95%-CI -0.51 to 0.07) | 0.133 | 1 |
| L-HDL-L | 1 | 0.33 | (95%-CI -0.02 to 0.68) | 0.062 | 0.867 |
|  | 2 | -0.23 | (95%-CI -0.55 to 0.08) | 0.149 | 1 |
|  | 3 | -0.28 | (95%-CI -0.65 to 0.08) | 0.13 | 1 |
| M-HDL-L | 1 | 0.23 | (95%-CI -0.07 to 0.54) | 0.128 | 1 |
|  | 2 | -0.19 | (95%-CI -0.49 to 0.12) | 0.224 | 1 |
|  | 3 | -0.11 | (95%-CI -0.47 to 0.25) | 0.547 | 1 |
| S-HDL-L | 1 | 0.2 | (95%-CI -0.12 to 0.52) | 0.213 | 1 |
|  | 2 | -0.12 | (95%-CI -0.46 to 0.21) | 0.457 | 1 |
|  | 3 | 0 | (95%-CI -0.36 to 0.36) | 0.998 | 1 |
|  |  |  |  |  |  |
|  | | |  |  |  |
|  | | |  |  |  |
|  | | |  |  |  |
|  | | |  |  |  |
|  | | |  |  |  |
| **Phospholipids in lipoprotein particles** | | |  |  |  |
| **Metabolite** | **Timepoint** | **Adjusted mean difference of metabolite change from baseline** | **95%-CI** | **Unadjusted p-value** | **Adjusted p-value** |
| XXL-VLDL-PL | 1 | -0.06 | (95%-CI -0.47 to 0.35) | 0.782 | 1 |
|  | 2 | -0.3 | (95%-CI -0.66 to 0.07) | 0.108 | 1 |
|  | 3 | -0.13 | (95%-CI -0.53 to 0.27) | 0.524 | 1 |
| XL-VLDL-PL | 1 | -0.02 | (95%-CI -0.43 to 0.4) | 0.94 | 1 |
|  | 2 | -0.4 | (95%-CI -0.81 to 0.01) | 0.058 | 0.815 |
|  | 3 | -0.23 | (95%-CI -0.67 to 0.22) | 0.313 | 1 |
| L-VLDL-PL | 1 | -0.04 | (95%-CI -0.44 to 0.37) | 0.856 | 1 |
|  | 2 | -0.17 | (95%-CI -0.54 to 0.2) | 0.355 | 1 |
|  | 3 | -0.06 | (95%-CI -0.47 to 0.35) | 0.78 | 1 |
| M-VLDL-PL | 1 | -0.27 | (95%-CI -0.64 to 0.09) | 0.138 | 1 |
|  | 2 | -0.22 | (95%-CI -0.5 to 0.07) | 0.139 | 1 |
|  | 3 | 0.15 | (95%-CI -0.17 to 0.46) | 0.358 | 1 |
| S-VLDL-PL | 1 | -0.19 | (95%-CI -0.6 to 0.21) | 0.347 | 1 |
|  | 2 | -0.26 | (95%-CI -0.59 to 0.06) | 0.114 | 1 |
|  | 3 | 0.01 | (95%-CI -0.35 to 0.37) | 0.969 | 1 |
| XS-VLDL-PL | 1 | -0.33 | (95%-CI -0.67 to 0.02) | 0.062 | 0.874 |
|  | 2 | 0.12 | (95%-CI -0.22 to 0.45) | 0.485 | 1 |
|  | 3 | 0.19 | (95%-CI -0.17 to 0.56) | 0.291 | 1 |
| IDL-PL | 1 | -0.3 | (95%-CI -0.63 to 0.03) | 0.073 | 1 |
|  | 2 | 0.18 | (95%-CI -0.14 to 0.5) | 0.275 | 1 |
|  | 3 | 0.31 | (95%-CI -0.04 to 0.66) | 0.082 | 1 |
| L-LDL-PL | 1 | -0.21 | (95%-CI -0.55 to 0.12) | 0.214 | 1 |
|  | 2 | 0.16 | (95%-CI -0.19 to 0.5) | 0.37 | 1 |
|  | 3 | 0.28 | (95%-CI -0.09 to 0.65) | 0.141 | 1 |
| M-LDL-PL | 1 | -0.31 | (95%-CI -0.67 to 0.05) | 0.087 | 1 |
|  | 2 | 0.05 | (95%-CI -0.35 to 0.46) | 0.792 | 1 |
|  | 3 | 0.24 | (95%-CI -0.19 to 0.67) | 0.27 | 1 |
| S-LDL-PL | 1 | -0.13 | (95%-CI -0.48 to 0.22) | 0.452 | 1 |
|  | 2 | -0.07 | (95%-CI -0.45 to 0.31) | 0.723 | 1 |
|  | 3 | 0.09 | (95%-CI -0.31 to 0.49) | 0.642 | 1 |
| XL-HDL-PL | 1 | 0.22 | (95%-CI -0.12 to 0.56) | 0.212 | 1 |
|  | 2 | -0.12 | (95%-CI -0.38 to 0.13) | 0.348 | 1 |
|  | 3 | -0.19 | (95%-CI -0.48 to 0.09) | 0.178 | 1 |
| L-HDL-PL | 1 | 0.33 | (95%-CI -0.01 to 0.68) | 0.057 | 0.797 |
|  | 2 | -0.23 | (95%-CI -0.56 to 0.09) | 0.16 | 1 |
|  | 3 | -0.27 | (95%-CI -0.64 to 0.1) | 0.151 | 1 |
| M-HDL-PL | 1 | 0.25 | (95%-CI -0.04 to 0.55) | 0.094 | 1 |
|  | 2 | -0.23 | (95%-CI -0.52 to 0.06) | 0.125 | 1 |
|  | 3 | -0.17 | (95%-CI -0.52 to 0.19) | 0.355 | 1 |
| S-HDL-PL | 1 | 0.33 | (95%-CI 0.02 to 0.64) | 0.04 | 0.553 |
|  | 2 | -0.25 | (95%-CI -0.53 to 0.02) | 0.07 | 0.986 |
|  | 3 | -0.12 | (95%-CI -0.42 to 0.17) | 0.415 | 1 |
|  | | |  |  |  |
|  | | |  |  |  |
|  | | |  |  |  |
|  | | |  |  |  |
|  | | |  |  |  |
|  | | |  |  |  |
| **Total cholesterol in lipoprotein particles** | | |  |  |  |
| **Metabolite** | **Timepoint** | **Adjusted mean difference of metabolite change from baseline** | **95%-CI** | **Unadjusted p-value** | **Adjusted p-value** |
| XXL-VLDL-C | 1 | -0.28 | (95%-CI -0.67 to 0.11) | 0.157 | 1 |
|  | 2 | -0.16 | (95%-CI -0.53 to 0.22) | 0.409 | 1 |
|  | 3 | -0.03 | (95%-CI -0.44 to 0.39) | 0.904 | 1 |
| XL-VLDL-C | 1 | -0.19 | (95%-CI -0.6 to 0.22) | 0.36 | 1 |
|  | 2 | -0.31 | (95%-CI -0.71 to 0.09) | 0.132 | 1 |
|  | 3 | -0.17 | (95%-CI -0.61 to 0.26) | 0.427 | 1 |
| L-VLDL-C | 1 | -0.12 | (95%-CI -0.52 to 0.29) | 0.566 | 1 |
|  | 2 | -0.18 | (95%-CI -0.54 to 0.19) | 0.343 | 1 |
|  | 3 | -0.04 | (95%-CI -0.45 to 0.37) | 0.845 | 1 |
| M-VLDL-C | 1 | -0.33 | (95%-CI -0.73 to 0.06) | 0.095 | 1 |
|  | 2 | -0.28 | (95%-CI -0.62 to 0.06) | 0.109 | 1 |
|  | 3 | 0.14 | (95%-CI -0.23 to 0.52) | 0.458 | 1 |
| S-VLDL-C | 1 | -0.35 | (95%-CI -0.72 to 0.02) | 0.064 | 0.9 |
|  | 2 | -0.02 | (95%-CI -0.37 to 0.33) | 0.895 | 1 |
|  | 3 | 0.22 | (95%-CI -0.16 to 0.61) | 0.248 | 1 |
| XS-VLDL-C | 1 | -0.43 | (95%-CI -0.77 to -0.09) | 0.014 | 0.199 |
|  | 2 | 0.2 | (95%-CI -0.14 to 0.55) | 0.247 | 1 |
|  | 3 | 0.29 | (95%-CI -0.08 to 0.66) | 0.12 | 1 |
| IDL-C | 1 | -0.32 | (95%-CI -0.65 to 0.01) | 0.06 | 0.836 |
|  | 2 | 0.16 | (95%-CI -0.16 to 0.49) | 0.322 | 1 |
|  | 3 | 0.32 | (95%-CI -0.04 to 0.67) | 0.077 | 1 |
| L-LDL-C | 1 | -0.23 | (95%-CI -0.57 to 0.1) | 0.175 | 1 |
|  | 2 | 0.2 | (95%-CI -0.15 to 0.54) | 0.261 | 1 |
|  | 3 | 0.3 | (95%-CI -0.08 to 0.67) | 0.121 | 1 |
| M-LDL-C | 1 | -0.34 | (95%-CI -0.74 to 0.06) | 0.092 | 1 |
|  | 2 | 0.13 | (95%-CI -0.33 to 0.59) | 0.573 | 1 |
|  | 3 | 0.45 | (95%-CI -0.04 to 0.93) | 0.07 | 0.978 |
| S-LDL-C | 1 | -0.19 | (95%-CI -0.59 to 0.21) | 0.345 | 1 |
|  | 2 | 0.04 | (95%-CI -0.4 to 0.48) | 0.866 | 1 |
|  | 3 | 0.3 | (95%-CI -0.16 to 0.76) | 0.2 | 1 |
| XL-HDL-C | 1 | 0.08 | (95%-CI -0.25 to 0.41) | 0.626 | 1 |
|  | 2 | -0.18 | (95%-CI -0.46 to 0.1) | 0.203 | 1 |
|  | 3 | -0.23 | (95%-CI -0.54 to 0.07) | 0.13 | 1 |
| L-HDL-C | 1 | 0.33 | (95%-CI -0.03 to 0.68) | 0.07 | 0.986 |
|  | 2 | -0.21 | (95%-CI -0.52 to 0.1) | 0.181 | 1 |
|  | 3 | -0.28 | (95%-CI -0.64 to 0.07) | 0.117 | 1 |
| M-HDL-C | 1 | 0.22 | (95%-CI -0.09 to 0.53) | 0.16 | 1 |
|  | 2 | -0.11 | (95%-CI -0.42 to 0.2) | 0.487 | 1 |
|  | 3 | -0.05 | (95%-CI -0.42 to 0.33) | 0.804 | 1 |
| S-HDL-C | 1 | -0.03 | (95%-CI -0.36 to 0.3) | 0.851 | 1 |
|  | 2 | 0.14 | (95%-CI -0.19 to 0.48) | 0.404 | 1 |
|  | 3 | 0.17 | (95%-CI -0.2 to 0.54) | 0.359 | 1 |
|  | | | |  |  |
|  | | | |  |  |
|  | | | |  |  |
|  | | | |  |  |
|  | | | |  |  |
|  | | | |  |  |
| **Cholesterol esters in lipoprotein particles** | | | |  |  |
| **Metabolite** | **Timepoint** | **Adjusted mean difference of metabolite change from baseline** | **95%-CI** | **Unadjusted p-value** | **Adjusted p-value** |
| XXL-VLDL-CE | 1 | -0.39 | (95%-CI -0.78 to 0.01) | 0.053 | 0.749 |
|  | 2 | -0.12 | (95%-CI -0.51 to 0.27) | 0.541 | 1 |
|  | 3 | -0.07 | (95%-CI -0.49 to 0.36) | 0.758 | 1 |
| XL-VLDL-CE | 1 | -0.24 | (95%-CI -0.62 to 0.15) | 0.23 | 1 |
|  | 2 | -0.22 | (95%-CI -0.61 to 0.16) | 0.256 | 1 |
|  | 3 | -0.13 | (95%-CI -0.54 to 0.29) | 0.538 | 1 |
| L-VLDL-CE | 1 | -0.21 | (95%-CI -0.6 to 0.19) | 0.306 | 1 |
|  | 2 | -0.13 | (95%-CI -0.48 to 0.22) | 0.463 | 1 |
|  | 3 | -0.02 | (95%-CI -0.41 to 0.36) | 0.902 | 1 |
| M-VLDL-CE | 1 | -0.45 | (95%-CI -0.87 to -0.03) | 0.035 | 0.493 |
|  | 2 | -0.18 | (95%-CI -0.57 to 0.21) | 0.358 | 1 |
|  | 3 | 0.12 | (95%-CI -0.3 to 0.55) | 0.571 | 1 |
| S-VLDL-CE | 1 | -0.38 | (95%-CI -0.74 to -0.02) | 0.04 | 0.558 |
|  | 2 | 0.11 | (95%-CI -0.23 to 0.46) | 0.52 | 1 |
|  | 3 | 0.32 | (95%-CI -0.05 to 0.7) | 0.093 | 1 |
| XS-VLDL-CE | 1 | -0.44 | (95%-CI -0.78 to -0.11) | 0.01 | 0.139 |
|  | 2 | 0.22 | (95%-CI -0.12 to 0.56) | 0.204 | 1 |
|  | 3 | 0.28 | (95%-CI -0.08 to 0.65) | 0.131 | 1 |
| IDL-CE | 1 | -0.33 | (95%-CI -0.66 to 0) | 0.05 | 0.694 |
|  | 2 | 0.15 | (95%-CI -0.17 to 0.48) | 0.354 | 1 |
|  | 3 | 0.32 | (95%-CI -0.04 to 0.67) | 0.078 | 1 |
| L-LDL-CE | 1 | -0.25 | (95%-CI -0.58 to 0.09) | 0.15 | 1 |
|  | 2 | 0.19 | (95%-CI -0.15 to 0.54) | 0.265 | 1 |
|  | 3 | 0.3 | (95%-CI -0.08 to 0.68) | 0.116 | 1 |
| M-LDL-CE | 1 | -0.37 | (95%-CI -0.76 to 0.02) | 0.06 | 0.844 |
|  | 2 | 0.19 | (95%-CI -0.23 to 0.6) | 0.373 | 1 |
|  | 3 | 0.45 | (95%-CI 0.01 to 0.89) | 0.044 | 0.611 |
| S-LDL-CE | 1 | -0.12 | (95%-CI -0.54 to 0.3) | 0.56 | 1 |
|  | 2 | 0.06 | (95%-CI -0.43 to 0.55) | 0.819 | 1 |
|  | 3 | 0.33 | (95%-CI -0.18 to 0.84) | 0.206 | 1 |
| XL-HDL-CE | 1 | 0.09 | (95%-CI -0.24 to 0.41) | 0.6 | 1 |
|  | 2 | -0.23 | (95%-CI -0.52 to 0.05) | 0.11 | 1 |
|  | 3 | -0.26 | (95%-CI -0.58 to 0.05) | 0.101 | 1 |
| L-HDL-CE | 1 | 0.34 | (95%-CI -0.01 to 0.69) | 0.059 | 0.827 |
|  | 2 | -0.23 | (95%-CI -0.54 to 0.08) | 0.15 | 1 |
|  | 3 | -0.29 | (95%-CI -0.65 to 0.06) | 0.105 | 1 |
| M-HDL-CE | 1 | 0.22 | (95%-CI -0.09 to 0.54) | 0.16 | 1 |
|  | 2 | -0.1 | (95%-CI -0.42 to 0.21) | 0.511 | 1 |
|  | 3 | -0.04 | (95%-CI -0.42 to 0.34) | 0.83 | 1 |
| S-HDL-CE | 1 | -0.1 | (95%-CI -0.43 to 0.23) | 0.544 | 1 |
|  | 2 | 0.2 | (95%-CI -0.12 to 0.52) | 0.225 | 1 |
|  | 3 | 0.19 | (95%-CI -0.16 to 0.54) | 0.297 | 1 |
|  | | |  |  |  |
|  | | |  |  |  |
|  | | |  |  |  |
|  | | |  |  |  |
|  | | |  |  |  |
|  | | |  |  |  |
| **Free cholesterol in lipoprotein particles** | | |  |  |  |
| **Metabolite** | **Timepoint** | **Adjusted mean difference of metabolite change from baseline** | **95%-CI** | **Unadjusted p-value** | **Adjusted p-value** |
| XXL-VLDL-FC | 1 | -0.14 | (95%-CI -0.53 to 0.26) | 0.502 | 1 |
|  | 2 | -0.24 | (95%-CI -0.62 to 0.14) | 0.21 | 1 |
|  | 3 | -0.01 | (95%-CI -0.43 to 0.4) | 0.95 | 1 |
| XL-VLDL-FC | 1 | -0.08 | (95%-CI -0.53 to 0.37) | 0.725 | 1 |
|  | 2 | -0.45 | (95%-CI -0.92 to 0.03) | 0.063 | 0.886 |
|  | 3 | -0.21 | (95%-CI -0.72 to 0.29) | 0.401 | 1 |
| L-VLDL-FC | 1 | 0.02 | (95%-CI -0.38 to 0.43) | 0.903 | 1 |
|  | 2 | -0.2 | (95%-CI -0.61 to 0.21) | 0.347 | 1 |
|  | 3 | -0.03 | (95%-CI -0.49 to 0.42) | 0.892 | 1 |
| M-VLDL-FC | 1 | -0.24 | (95%-CI -0.61 to 0.13) | 0.208 | 1 |
|  | 2 | -0.33 | (95%-CI -0.64 to -0.01) | 0.041 | 0.568 |
|  | 3 | 0.13 | (95%-CI -0.22 to 0.47) | 0.462 | 1 |
| S-VLDL-FC | 1 | -0.23 | (95%-CI -0.62 to 0.16) | 0.239 | 1 |
|  | 2 | -0.24 | (95%-CI -0.59 to 0.11) | 0.177 | 1 |
|  | 3 | 0.02 | (95%-CI -0.37 to 0.4) | 0.925 | 1 |
| XS-VLDL-FC | 1 | -0.35 | (95%-CI -0.69 to -0.01) | 0.045 | 0.637 |
|  | 2 | 0.14 | (95%-CI -0.21 to 0.5) | 0.42 | 1 |
|  | 3 | 0.29 | (95%-CI -0.09 to 0.67) | 0.13 | 1 |
| IDL-FC | 1 | -0.26 | (95%-CI -0.59 to 0.07) | 0.117 | 1 |
|  | 2 | 0.18 | (95%-CI -0.15 to 0.5) | 0.278 | 1 |
|  | 3 | 0.3 | (95%-CI -0.05 to 0.65) | 0.096 | 1 |
| L-LDL-FC | 1 | -0.18 | (95%-CI -0.52 to 0.15) | 0.289 | 1 |
|  | 2 | 0.19 | (95%-CI -0.14 to 0.53) | 0.257 | 1 |
|  | 3 | 0.27 | (95%-CI -0.1 to 0.64) | 0.147 | 1 |
| M-LDL-FC | 1 | -0.29 | (95%-CI -0.68 to 0.09) | 0.134 | 1 |
|  | 2 | 0.08 | (95%-CI -0.36 to 0.52) | 0.729 | 1 |
|  | 3 | 0.33 | (95%-CI -0.14 to 0.79) | 0.169 | 1 |
| S-LDL-FC | 1 | -0.17 | (95%-CI -0.52 to 0.18) | 0.331 | 1 |
|  | 2 | 0.02 | (95%-CI -0.36 to 0.39) | 0.935 | 1 |
|  | 3 | 0.18 | (95%-CI -0.22 to 0.57) | 0.376 | 1 |
| XL-HDL-FC | 1 | 0.06 | (95%-CI -0.27 to 0.4) | 0.72 | 1 |
|  | 2 | -0.02 | (95%-CI -0.29 to 0.25) | 0.876 | 1 |
|  | 3 | -0.14 | (95%-CI -0.44 to 0.16) | 0.347 | 1 |
| L-HDL-FC | 1 | 0.28 | (95%-CI -0.07 to 0.63) | 0.121 | 1 |
|  | 2 | -0.15 | (95%-CI -0.45 to 0.15) | 0.32 | 1 |
|  | 3 | -0.24 | (95%-CI -0.59 to 0.1) | 0.165 | 1 |
| M-HDL-FC | 1 | 0.21 | (95%-CI -0.1 to 0.52) | 0.177 | 1 |
|  | 2 | -0.13 | (95%-CI -0.44 to 0.18) | 0.42 | 1 |
|  | 3 | -0.07 | (95%-CI -0.44 to 0.3) | 0.715 | 1 |
| S-HDL-FC | 1 | 0.3 | (95%-CI -0.02 to 0.62) | 0.066 | 0.926 |
|  | 2 | -0.2 | (95%-CI -0.52 to 0.11) | 0.209 | 1 |
|  | 3 | -0.03 | (95%-CI -0.38 to 0.31) | 0.842 | 1 |
|  | | |  |  |  |
|  | | |  |  |  |
|  | | |  |  |  |
|  | | |  |  |  |
|  | | |  |  |  |
|  | | |  |  |  |
| **Triglycerides in lipoprotein particles** | | |  |  |  |
| **Metabolite** | **Timepoint** | **Adjusted mean difference of metabolite change from baseline** | **95%-CI** | **Unadjusted p-value** | **Adjusted p-value** |
| XXL-VLDL-TG | 1 | -0.11 | (95%-CI -0.5 to 0.29) | 0.594 | 1 |
|  | 2 | -0.26 | (95%-CI -0.61 to 0.1) | 0.153 | 1 |
|  | 3 | -0.23 | (95%-CI -0.61 to 0.16) | 0.251 | 1 |
| XL-VLDL-TG | 1 | 0.02 | (95%-CI -0.37 to 0.42) | 0.902 | 1 |
|  | 2 | -0.35 | (95%-CI -0.71 to 0.02) | 0.065 | 0.911 |
|  | 3 | -0.24 | (95%-CI -0.63 to 0.16) | 0.237 | 1 |
| L-VLDL-TG | 1 | 0.02 | (95%-CI -0.37 to 0.41) | 0.936 | 1 |
|  | 2 | -0.2 | (95%-CI -0.56 to 0.15) | 0.262 | 1 |
|  | 3 | -0.1 | (95%-CI -0.5 to 0.3) | 0.617 | 1 |
| M-VLDL-TG | 1 | -0.23 | (95%-CI -0.58 to 0.12) | 0.193 | 1 |
|  | 2 | -0.19 | (95%-CI -0.47 to 0.09) | 0.187 | 1 |
|  | 3 | 0.12 | (95%-CI -0.19 to 0.43) | 0.457 | 1 |
| S-VLDL-TG | 1 | -0.21 | (95%-CI -0.59 to 0.16) | 0.266 | 1 |
|  | 2 | -0.21 | (95%-CI -0.52 to 0.1) | 0.187 | 1 |
|  | 3 | -0.02 | (95%-CI -0.36 to 0.32) | 0.896 | 1 |
| XS-VLDL-TG | 1 | -0.17 | (95%-CI -0.54 to 0.19) | 0.352 | 1 |
|  | 2 | -0.26 | (95%-CI -0.58 to 0.07) | 0.126 | 1 |
|  | 3 | 0.04 | (95%-CI -0.31 to 0.4) | 0.816 | 1 |
| IDL-TG | 1 | -0.09 | (95%-CI -0.48 to 0.29) | 0.625 | 1 |
|  | 2 | -0.26 | (95%-CI -0.66 to 0.15) | 0.212 | 1 |
|  | 3 | 0.02 | (95%-CI -0.41 to 0.46) | 0.916 | 1 |
| L-LDL-TG | 1 | -0.01 | (95%-CI -0.4 to 0.38) | 0.95 | 1 |
|  | 2 | -0.38 | (95%-CI -0.82 to 0.05) | 0.084 | 1 |
|  | 3 | 0.04 | (95%-CI -0.44 to 0.51) | 0.874 | 1 |
| M-LDL-TG | 1 | 0 | (95%-CI -0.38 to 0.38) | 0.999 | 1 |
|  | 2 | -0.45 | (95%-CI -0.87 to -0.03) | 0.036 | 0.502 |
|  | 3 | -0.22 | (95%-CI -0.66 to 0.23) | 0.335 | 1 |
| S-LDL-TG | 1 | 0.02 | (95%-CI -0.37 to 0.41) | 0.921 | 1 |
|  | 2 | -0.51 | (95%-CI -0.89 to -0.14) | 0.007 | 0.103 |
|  | 3 | -0.25 | (95%-CI -0.64 to 0.14) | 0.211 | 1 |
| XL-HDL-TG | 1 | -0.11 | (95%-CI -0.52 to 0.3) | 0.605 | 1 |
|  | 2 | -0.5 | (95%-CI -0.92 to -0.08) | 0.019 | 0.27 |
|  | 3 | -0.17 | (95%-CI -0.63 to 0.28) | 0.46 | 1 |
| L-HDL-TG | 1 | 0.22 | (95%-CI -0.2 to 0.63) | 0.307 | 1 |
|  | 2 | -0.65 | (95%-CI -1.14 to -0.17) | 0.009 | 0.119 |
|  | 3 | -0.09 | (95%-CI -0.64 to 0.46) | 0.759 | 1 |
| M-HDL-TG | 1 | 0.04 | (95%-CI -0.33 to 0.41) | 0.833 | 1 |
|  | 2 | -0.43 | (95%-CI -0.75 to -0.11) | 0.009 | 0.127 |
|  | 3 | -0.23 | (95%-CI -0.62 to 0.16) | 0.241 | 1 |
| S-HDL-TG | 1 | 0.02 | (95%-CI -0.36 to 0.4) | 0.92 | 1 |
|  | 2 | -0.43 | (95%-CI -0.79 to -0.08) | 0.016 | 0.224 |
|  | 3 | -0.23 | (95%-CI -0.61 to 0.15) | 0.236 | 1 |
|  | | |  |  |  |
|  | | |  |  |  |
|  | | |  |  |  |
|  | | |  |  |  |
|  | | |  |  |  |
|  | | |  |  |  |
| **Diameter of lipoprotein particles** | | |  |  |  |
| **Metabolite** | **Timepoint** | **Adjusted mean difference of metabolite change from baseline** | **95%-CI** | **Unadjusted p-value** | **Adjusted p-value** |
| VLDL-D | 1 | -0.06 | (95%-CI -0.41 to 0.3) | 0.755 | 1 |
|  | 2 | -0.21 | (95%-CI -0.5 to 0.08) | 0.153 | 1 |
|  | 3 | 0.02 | (95%-CI -0.29 to 0.34) | 0.879 | 1 |
| LDL-D | 1 | 0.09 | (95%-CI -0.53 to 0.72) | 0.774 | 1 |
|  | 2 | 0.33 | (95%-CI -0.33 to 0.99) | 0.319 | 1 |
|  | 3 | -0.07 | (95%-CI -0.78 to 0.65) | 0.855 | 1 |
| HDL-D | 1 | 0.25 | (95%-CI -0.12 to 0.62) | 0.178 | 1 |
|  | 2 | -0.13 | (95%-CI -0.43 to 0.17) | 0.388 | 1 |
|  | 3 | -0.09 | (95%-CI -0.41 to 0.23) | 0.575 | 1 |
| **Cholesterol** |  |  |  |  |  |
| Serum-C | 1 | -0.2 | (95%-CI -0.53 to 0.12) | 0.225 | 1 |
|  | 2 | 0.1 | (95%-CI -0.2 to 0.4) | 0.518 | 1 |
|  | 3 | 0.12 | (95%-CI -0.21 to 0.45) | 0.474 | 1 |
| VLDL-C | 1 | -0.32 | (95%-CI -0.75 to 0.11) | 0.146 | 1 |
|  | 2 | -0.19 | (95%-CI -0.61 to 0.22) | 0.355 | 1 |
|  | 3 | 0.14 | (95%-CI -0.31 to 0.59) | 0.543 | 1 |
| Remnant-C | 1 | -0.39 | (95%-CI -0.73 to -0.05) | 0.024 | 0.33 |
|  | 2 | 0.07 | (95%-CI -0.23 to 0.37) | 0.654 | 1 |
|  | 3 | 0.14 | (95%-CI -0.19 to 0.47) | 0.412 | 1 |
| LDL-C | 1 | -0.29 | (95%-CI -0.61 to 0.02) | 0.07 | 0.974 |
|  | 2 | 0.22 | (95%-CI -0.06 to 0.5) | 0.124 | 1 |
|  | 3 | 0.2 | (95%-CI -0.1 to 0.51) | 0.193 | 1 |
| HDL-C | 1 | 0.33 | (95%-CI 0.01 to 0.64) | 0.04 | 0.565 |
|  | 2 | -0.13 | (95%-CI -0.39 to 0.13) | 0.329 | 1 |
|  | 3 | -0.14 | (95%-CI -0.43 to 0.14) | 0.324 | 1 |
| HDL2-C | 1 | 0.28 | (95%-CI -0.04 to 0.61) | 0.088 | 1 |
|  | 2 | -0.08 | (95%-CI -0.36 to 0.19) | 0.556 | 1 |
|  | 3 | -0.09 | (95%-CI -0.39 to 0.22) | 0.573 | 1 |
| HDL3-C | 1 | 0.23 | (95%-CI -0.12 to 0.58) | 0.194 | 1 |
|  | 2 | -0.27 | (95%-CI -0.58 to 0.04) | 0.089 | 1 |
|  | 3 | -0.13 | (95%-CI -0.47 to 0.21) | 0.458 | 1 |
| EstC | 1 | -0.02 | (95%-CI -0.39 to 0.35) | 0.923 | 1 |
|  | 2 | -0.05 | (95%-CI -0.44 to 0.34) | 0.799 | 1 |
|  | 3 | 0.04 | (95%-CI -0.37 to 0.45) | 0.849 | 1 |
| FreeC | 1 | -0.07 | (95%-CI -0.44 to 0.29) | 0.684 | 1 |
|  | 2 | -0.15 | (95%-CI -0.51 to 0.21) | 0.413 | 1 |
|  | 3 | 0.07 | (95%-CI -0.31 to 0.45) | 0.713 | 1 |
|  | | |  |  |  |
|  | | |  |  |  |
|  | | |  |  |  |
|  | | |  |  |  |
|  | | |  |  |  |
|  | | |  |  |  |
|  | | |  |  |  |
|  | | |  |  |  |
|  | | |  |  |  |
|  | | |  |  |  |
|  | | |  |  |  |
| **Glycerides and phospholipids** | | |  |  |  |
| **Metabolite** | **Timepoint** | **Adjusted mean difference of metabolite change from baseline** | **95%-CI** | **Unadjusted p-value** | **Adjusted p-value** |
| Serum-TG | 1 | -0.17 | (95%-CI -0.54 to 0.2) | 0.355 | 1 |
|  | 2 | -0.3 | (95%-CI -0.57 to -0.02) | 0.036 | 0.507 |
|  | 3 | 0 | (95%-CI -0.31 to 0.3) | 0.983 | 1 |
| VLDL-TG | 1 | -0.18 | (95%-CI -0.54 to 0.17) | 0.308 | 1 |
|  | 2 | -0.22 | (95%-CI -0.49 to 0.05) | 0.116 | 1 |
|  | 3 | 0.07 | (95%-CI -0.22 to 0.37) | 0.633 | 1 |
| LDL-TG | 1 | -0.06 | (95%-CI -0.46 to 0.33) | 0.748 | 1 |
|  | 2 | -0.39 | (95%-CI -0.8 to 0.01) | 0.059 | 0.82 |
|  | 3 | -0.21 | (95%-CI -0.65 to 0.23) | 0.344 | 1 |
| HDL-TG | 1 | -0.01 | (95%-CI -0.36 to 0.34) | 0.953 | 1 |
|  | 2 | -0.42 | (95%-CI -0.75 to -0.08) | 0.014 | 0.2 |
|  | 3 | -0.13 | (95%-CI -0.5 to 0.23) | 0.465 | 1 |
| TotPG | 1 | -0.15 | (95%-CI -0.6 to 0.29) | 0.504 | 1 |
|  | 2 | -0.31 | (95%-CI -0.83 to 0.21) | 0.246 | 1 |
|  | 3 | 0.09 | (95%-CI -0.46 to 0.63) | 0.756 | 1 |
| TG-PG | 1 | -0.11 | (95%-CI -0.55 to 0.33) | 0.625 | 1 |
|  | 2 | -0.24 | (95%-CI -0.7 to 0.21) | 0.292 | 1 |
|  | 3 | 0.01 | (95%-CI -0.47 to 0.5) | 0.952 | 1 |
| PC | 1 | -0.11 | (95%-CI -0.54 to 0.32) | 0.605 | 1 |
|  | 2 | -0.29 | (95%-CI -0.79 to 0.21) | 0.253 | 1 |
|  | 3 | 0.05 | (95%-CI -0.47 to 0.57) | 0.852 | 1 |
| SM | 1 | -0.2 | (95%-CI -0.75 to 0.36) | 0.486 | 1 |
|  | 2 | -0.19 | (95%-CI -0.74 to 0.37) | 0.501 | 1 |
|  | 3 | 0.19 | (95%-CI -0.39 to 0.78) | 0.511 | 1 |
| TotCho | 1 | -0.18 | (95%-CI -0.66 to 0.29) | 0.448 | 1 |
|  | 2 | -0.27 | (95%-CI -0.82 to 0.28) | 0.329 | 1 |
|  | 3 | 0.18 | (95%-CI -0.4 to 0.75) | 0.54 | 1 |
| **Apolipoproteins** | |  |  |  |  |
| **Metabolite** | **Timepoint** | **Adjusted mean difference of metabolite change from baseline** | **95%-CI** | **Unadjusted p-value** | **Adjusted p-value** |
| ApoA1 | 1 | 0.17 | (95%-CI -0.15 to 0.49) | 0.299 | 1 |
|  | 2 | -0.15 | (95%-CI -0.46 to 0.17) | 0.362 | 1 |
|  | 3 | -0.05 | (95%-CI -0.39 to 0.29) | 0.769 | 1 |
| ApoB | 1 | -0.38 | (95%-CI -0.73 to -0.04) | 0.03 | 0.415 |
|  | 2 | 0.01 | (95%-CI -0.29 to 0.3) | 0.962 | 1 |
|  | 3 | 0.09 | (95%-CI -0.23 to 0.41) | 0.565 | 1 |
| ApoB-ApoA1 | 1 | -0.51 | (95%-CI -0.87 to -0.15) | 0.006 | 0.083 |
|  | 2 | 0.05 | (95%-CI -0.2 to 0.31) | 0.692 | 1 |
|  | 3 | 0.03 | (95%-CI -0.25 to 0.31) | 0.831 | 1 |
|  |  |  |  |  |  |
|  |  |  |  |  |  |
|  |  |  |  |  |  |
|  |  |  |  |  |  |
|  |  |  |  |  |  |
|  |  |  |  |  |  |
|  |  |  |  |  |  |
|  |  |  |  |  |  |
|  |  |  |  |  |  |
|  |  |  |  |  |  |
| **Fatty acids** |  |  |  |  |  |
| **Metabolite** | **Timepoint** | **Adjusted mean difference of metabolite change from baseline** | **95%-CI** | **Unadjusted p-value** | **Adjusted p-value** |
| TotFA | 1 | -0.17 | (95%-CI -0.64 to 0.3) | 0.477 | 1 |
|  | 2 | -0.31 | (95%-CI -0.81 to 0.19) | 0.224 | 1 |
|  | 3 | -0.26 | (95%-CI -0.85 to 0.32) | 0.368 | 1 |
| UnSat | 1 | -0.04 | (95%-CI -0.54 to 0.47) | 0.891 | 1 |
|  | 2 | 0.01 | (95%-CI -0.49 to 0.5) | 0.984 | 1 |
|  | 3 | 0.32 | (95%-CI -0.25 to 0.89) | 0.272 | 1 |
| DHA | 1 | -0.37 | (95%-CI -0.84 to 0.1) | 0.124 | 1 |
|  | 2 | -0.16 | (95%-CI -0.58 to 0.26) | 0.456 | 1 |
|  | 3 | 0.18 | (95%-CI -0.31 to 0.68) | 0.465 | 1 |
| LA | 1 | 0.11 | (95%-CI -0.3 to 0.51) | 0.6 | 1 |
|  | 2 | -0.32 | (95%-CI -0.76 to 0.11) | 0.146 | 1 |
|  | 3 | -0.29 | (95%-CI -0.8 to 0.22) | 0.267 | 1 |
| FAw3 | 1 | -0.35 | (95%-CI -0.78 to 0.08) | 0.106 | 1 |
|  | 2 | -0.09 | (95%-CI -0.5 to 0.33) | 0.683 | 1 |
|  | 3 | 0.07 | (95%-CI -0.42 to 0.56) | 0.772 | 1 |
| FAw6 | 1 | -0.04 | (95%-CI -0.46 to 0.38) | 0.856 | 1 |
|  | 2 | -0.26 | (95%-CI -0.74 to 0.21) | 0.273 | 1 |
|  | 3 | -0.22 | (95%-CI -0.77 to 0.33) | 0.432 | 1 |
| PUFA | 1 | -0.12 | (95%-CI -0.55 to 0.32) | 0.596 | 1 |
|  | 2 | -0.24 | (95%-CI -0.72 to 0.25) | 0.336 | 1 |
|  | 3 | -0.17 | (95%-CI -0.74 to 0.39) | 0.541 | 1 |
| MUFA | 1 | -0.14 | (95%-CI -0.63 to 0.36) | 0.587 | 1 |
|  | 2 | -0.43 | (95%-CI -0.95 to 0.08) | 0.1 | 1 |
|  | 3 | -0.32 | (95%-CI -0.92 to 0.28) | 0.289 | 1 |
| SFA | 1 | -0.21 | (95%-CI -0.66 to 0.24) | 0.357 | 1 |
|  | 2 | -0.26 | (95%-CI -0.73 to 0.2) | 0.259 | 1 |
|  | 3 | -0.28 | (95%-CI -0.81 to 0.26) | 0.311 | 1 |
| DHA-FA | 1 | -0.14 | (95%-CI -0.65 to 0.36) | 0.571 | 1 |
|  | 2 | 0.02 | (95%-CI -0.48 to 0.51) | 0.944 | 1 |
|  | 3 | 0.23 | (95%-CI -0.35 to 0.81) | 0.425 | 1 |
| LA-FA | 1 | 0.3 | (95%-CI -0.13 to 0.73) | 0.166 | 1 |
|  | 2 | -0.03 | (95%-CI -0.43 to 0.36) | 0.862 | 1 |
|  | 3 | -0.1 | (95%-CI -0.57 to 0.36) | 0.666 | 1 |
| FAw3-FA | 1 | -0.32 | (95%-CI -0.72 to 0.07) | 0.106 | 1 |
|  | 2 | 0.22 | (95%-CI -0.13 to 0.57) | 0.225 | 1 |
|  | 3 | 0.35 | (95%-CI -0.06 to 0.76) | 0.091 | 1 |
| FAw6-FA | 1 | 0.25 | (95%-CI -0.18 to 0.67) | 0.258 | 1 |
|  | 2 | 0.16 | (95%-CI -0.22 to 0.54) | 0.406 | 1 |
|  | 3 | 0.1 | (95%-CI -0.34 to 0.55) | 0.643 | 1 |
| PUFA-FA | 1 | 0.14 | (95%-CI -0.29 to 0.56) | 0.53 | 1 |
|  | 2 | 0.23 | (95%-CI -0.18 to 0.65) | 0.269 | 1 |
|  | 3 | 0.23 | (95%-CI -0.26 to 0.72) | 0.348 | 1 |
| MUFA-FA | 1 | 0 | (95%-CI -0.49 to 0.49) | 0.989 | 1 |
|  | 2 | -0.46 | (95%-CI -0.97 to 0.04) | 0.072 | 1 |
|  | 3 | -0.25 | (95%-CI -0.84 to 0.34) | 0.402 | 1 |
| SFA-FA | 1 | -0.24 | (95%-CI -0.81 to 0.33) | 0.398 | 1 |
|  | 2 | 0.18 | (95%-CI -0.5 to 0.86) | 0.608 | 1 |
|  | 3 | -0.17 | (95%-CI -0.95 to 0.62) | 0.677 | 1 |
| **Glycolysis related** | |  |  |  |  |
| **Metabolite** | **Timepoint** | **Adjusted mean difference of metabolite change from baseline** | **95%-CI** | **Unadjusted p-value** | **Adjusted p-value** |
| Glc | 1 | -0.19 | (95%-CI -0.5 to 0.13) | 0.25 | 1 |
|  | 2 | 0 | (95%-CI -0.39 to 0.39) | 0.982 | 1 |
|  | 3 | 0.3 | (95%-CI -0.12 to 0.72) | 0.165 | 1 |
| Lac | 1 | 0.17 | (95%-CI -0.2 to 0.55) | 0.369 | 1 |
|  | 2 | 0.18 | (95%-CI -0.21 to 0.58) | 0.356 | 1 |
|  | 3 | -0.07 | (95%-CI -0.5 to 0.35) | 0.736 | 1 |
| Cit | 1 | 0.2 | (95%-CI -0.71 to 1.12) | 0.664 | 1 |
|  | 2 | -0.11 | (95%-CI -1.36 to 1.15) | 0.868 | 1 |
|  | 3 | -0.37 | (95%-CI -1.71 to 0.98) | 0.591 | 1 |
| **Amino acids** |  |  |  |  |  |
| **Metabolite** | **Timepoint** | **Adjusted mean difference of metabolite change from baseline** | **95%-CI** | **Unadjusted p-value** | **Adjusted p-value** |
| Ala | 1 | 0.03 | (95%-CI -0.35 to 0.42) | 0.863 | 1 |
|  | 2 | 0.09 | (95%-CI -0.34 to 0.52) | 0.692 | 1 |
|  | 3 | -0.11 | (95%-CI -0.58 to 0.36) | 0.639 | 1 |
| Gln | 1 | 0.22 | (95%-CI -0.15 to 0.59) | 0.247 | 1 |
|  | 2 | -0.35 | (95%-CI -0.8 to 0.1) | 0.125 | 1 |
|  | 3 | 0.02 | (95%-CI -0.46 to 0.5) | 0.935 | 1 |
| His | 1 | 0.35 | (95%-CI -0.01 to 0.72) | 0.056 | 0.783 |
|  | 2 | -0.12 | (95%-CI -0.55 to 0.3) | 0.57 | 1 |
|  | 3 | -0.16 | (95%-CI -0.63 to 0.32) | 0.513 | 1 |
| Ile | 1 | 0.13 | (95%-CI -0.37 to 0.64) | 0.603 | 1 |
|  | 2 | -0.38 | (95%-CI -1.01 to 0.25) | 0.231 | 1 |
|  | 3 | 0.1 | (95%-CI -0.58 to 0.78) | 0.776 | 1 |
| Leu | 1 | 0.34 | (95%-CI -0.13 to 0.82) | 0.154 | 1 |
|  | 2 | -0.31 | (95%-CI -0.87 to 0.25) | 0.281 | 1 |
|  | 3 | -0.13 | (95%-CI -0.73 to 0.47) | 0.671 | 1 |
| Val | 1 | 0.49 | (95%-CI 0.16 to 0.83) | 0.004 | 0.058 |
|  | 2 | -0.27 | (95%-CI -0.7 to 0.16) | 0.222 | 1 |
|  | 3 | -0.35 | (95%-CI -0.82 to 0.11) | 0.137 | 1 |
| Phe | 1 | 0.25 | (95%-CI -0.16 to 0.66) | 0.225 | 1 |
|  | 2 | -0.24 | (95%-CI -0.78 to 0.3) | 0.382 | 1 |
|  | 3 | -0.08 | (95%-CI -0.67 to 0.5) | 0.777 | 1 |
| Tyr | 1 | 0.09 | (95%-CI -0.38 to 0.56) | 0.704 | 1 |
|  | 2 | 0.17 | (95%-CI -0.41 to 0.75) | 0.566 | 1 |
|  | 3 | -0.39 | (95%-CI -1.03 to 0.24) | 0.225 | 1 |
| **Ketone bodies** | |  |  |  |  |
| **Metabolite** | **Timepoint** | **Adjusted mean difference of metabolite change from baseline** | **95%-CI** | **Unadjusted p-value** | **Adjusted p-value** |
| Ace | 1 | 0.28 | (95%-CI -0.05 to 0.62) | 0.1 | 1 |
|  | 2 | -0.13 | (95%-CI -0.6 to 0.35) | 0.599 | 1 |
|  | 3 | -0.4 | (95%-CI -0.91 to 0.1) | 0.114 | 1 |
| AcAce | 1 | 0.43 | (95%-CI -0.2 to 1.05) | 0.18 | 1 |
|  | 2 | -0.07 | (95%-CI -0.94 to 0.8) | 0.873 | 1 |
|  | 3 | 0.27 | (95%-CI -0.66 to 1.2) | 0.571 | 1 |
| bOHBut | 1 | 0.26 | (95%-CI -0.1 to 0.62) | 0.155 | 1 |
|  | 2 | -0.24 | (95%-CI -0.7 to 0.22) | 0.301 | 1 |
|  | 3 | 0.14 | (95%-CI -0.36 to 0.64) | 0.576 | 1 |
|  | |  |  |  |  |
|  | |  |  |  |  |
| **Albumin and creatinine** | |  |  |  |  |
| **Metabolite** | **Timepoint** | **Adjusted mean difference of metabolite change from baseline** | **95%-CI** | **Unadjusted p-value** | **Adjusted p-value** |
| Crea | 1 | 0.16 | (95%-CI -0.15 to 0.47) | 0.306 | 1 |
|  | 2 | -0.2 | (95%-CI -0.56 to 0.15) | 0.258 | 1 |
|  | 3 | -0.38 | (95%-CI -0.77 to 0.01) | 0.057 | 0.797 |
| Alb | 1 | 0.26 | (95%-CI -0.06 to 0.57) | 0.109 | 1 |
|  | 2 | -0.3 | (95%-CI -0.67 to 0.08) | 0.117 | 1 |
|  | 3 | -0.09 | (95%-CI -0.49 to 0.31) | 0.656 | 1 |
| **Inflammation** | |  |  |  |  |
| **Metabolite** | **Timepoint** | **Adjusted mean difference of metabolite change from baseline** | **95%-CI** | **Unadjusted p-value** | **Adjusted p-value** |
| Gp | 1 | -0.13 | (95%-CI -0.53 to 0.28) | 0.534 | 1 |
|  | 2 | -0.42 | (95%-CI -0.81 to -0.04) | 0.031 | 0.436 |
|  | 3 | 0.12 | (95%-CI -0.3 to 0.55) | 0.573 | 1 |
